# Simulated distributions from negative experiments highlight the importance of the body mass index distribution in explaining depression–body mass index genetic risk score interactions

**DOI:** 10.1093/ije/dyac052

**Published:** 2022-04-07

**Authors:** Francesco Casanova, Jessica O’Loughlin, Cathryn Lewis, Timothy M Frayling, Andrew R Wood, Jessica Tyrrell

**Affiliations:** Genetics of Complex Traits, Institute of Biomedical and Clinical Science, University of Exeter Medical School, Exeter, UK; Genetics of Complex Traits, Institute of Biomedical and Clinical Science, University of Exeter Medical School, Exeter, UK; Social, Genetic and Developmental Psychiatry Centre, Institute of Psychiatry, Psychology & Neuroscience, King’s College London, London, UK; Department of Medical & Molecular Genetics, Faculty of Life Sciences and Medicine, King’s College London, London, UK; Genetics of Complex Traits, Institute of Biomedical and Clinical Science, University of Exeter Medical School, Exeter, UK; Genetics of Complex Traits, Institute of Biomedical and Clinical Science, University of Exeter Medical School, Exeter, UK; Genetics of Complex Traits, Institute of Biomedical and Clinical Science, University of Exeter Medical School, Exeter, UK

**Keywords:** Depression, obesity, gene–environment interaction, UK Biobank

## Abstract

**Background:**

Depression and obesity are complex global health problems. Recent studies suggest that a genetic predisposition to obesity might be accentuated in people with depression, but these analyses are prone to bias. Here, we tested the hypothesis that depression accentuates genetic susceptibility to obesity and applied negative control experiments to test whether any observed interactions were real or driven by confounding and statistical biases.

**Methods:**

We used data from up to 378 000 Europeans in UK Biobank, a 73 variant body mass index (BMI) genetic risk score, two depression measures [depression symptoms (DS), major depression (MD)] and an antidepressant usage variable available. We tested whether (i) depression and (ii) antidepressant treatment accentuated genetic susceptibility to obesity. Finally, we performed negative control experiments by sampling individuals at random so that they had BMI distributions identical to depression cases and controls.

**Results:**

Depression was associated with an accentuation of an individual’s genetic risk of obesity with evidence of interactions for both DS and MD (*P_interaction_ *=* *7* *×* *10^–4^ and 7* *×* *10^–5^ respectively). Antidepressant usage within DS cases accentuated genetic obesity risk (*P_interaction_ *=* *9* *×* *10^–4^), but not for MD (*P_interaction_ *=* *0.13). Negative control experiments suggested that the observed interactions for MD (empirical-*P *=* *0.067) may be driven by statistical biases or confounding factors but were not possible with the larger DS groups. Antidepressant usage interaction also appears to be driven by statistical artefacts (empirical-*P* = 0.510 using MD and 0.162 using DS).

**Conclusion:**

We have highlighted the importance of running negative experiments to confirm putative interactions in gene–environment studies. We provide some tentative evidence that depression accentuates an individual’s genetic susceptibility to higher BMI but demonstrated that the BMI distributions within cases and controls might drive these interactions.

Key Messages

- This study provides evidence suggesting that depression and depression severity accentuate an individual’s genetic susceptibility to higher body mass index (BMI).
- These types of interaction, known as gene–environment interactions, are prone to statistical bias so we performed negative experiments to confirm the observed interaction.
- Our negative test results demonstrate that observed interactions may be driven by BMI distributions and therefore our study highlights the importance of testing putative gene–environment interaction using negative experiments.

## Background

Depression and obesity are global health problems that severely impact health services and cost billions annually. Recent studies have highlighted additional disease burden when obesity and depression are co-morbid.^1^ The relationship between these two diseases is complex, involving genetic and environmental factors, with twin studies suggesting 12% shared genetics.^2^ The genetic approach of Mendelian randomization^3^ has provided evidence that bidirectional associations exist and are, at least partially, causal.^4^^,^^5^

Further evidence demonstrating the complexity of the relationship between obesity and depression comes from recent studies suggesting genetic variants associated with body mass index (BMI) and a BMI genetic risk score (BMI–GRS) may have stronger effects on BMI in people with depression.^5^^,^^6^ For example, Mulugeta *et al.* report a BMI–GRS by depression status interaction as well as rs6567160 in the *MC4R* gene having a stronger effect on BMI in depression cases than controls (β = 0.166 vs 0.100).^5^ Moreover, a single-nucleotide polymorphism (SNP) in the *FTO* gene (rs9939609) was associated with log_10_BMI in depression cases (β = 0.12) but not in controls (β = 0.02).^6^ It is currently unknown whether other SNPs associated with higher BMI show similar interactions.

The different effects of the BMI–GRS and BMI SNPs on BMI in depressed individuals vs controls provide evidence of an interaction between depression status and BMI genetics to accentuate an individual’s genetic risk of obesity. However, these interactions may not be specific to depression, but a feature of selecting groups of individuals with a higher BMI (i.e. individuals with depression) and comparing them to groups of individuals of lower BMI (i.e. individuals without depression).^7^ The effect of having different BMI distributions can be tested by performing negative control experiments in which individuals are randomly sampled from a cohort to recreate two distributions, e.g. BMI distributions, identical to those seen for depression cases and controls. Previous work demonstrated that deprivation as measured by the Townsend deprivation index (TDI) interacted with BMI genetics to accentuate the genetic risk of obesity in deprived individuals.^7^ Recreating these BMI distributions randomly regardless of TDI 100 times did not provide the same evidence of interaction suggesting that TDI really does accentuate an individual’s genetic risk for obesity. To the best of our knowledge no studies have used negative control experiments to test whether depression truly interacts with BMI genetics and therefore it remains unclear whether any of the reported interactions are driven by confounding or statistical artefacts.

The obesity and depression relationship is further complicated by the heterogeneity of definitions of depression that can be used in different studies^8^ and the effect of antidepressant treatment on weight changes, with most classes of antidepressants having some association with weight gain.^9^^,^^10^ No studies have tested whether antidepressants accentuate an individual’s genetic risk for obesity.

Here, we used the UK Biobank study to replicate previous findings in a larger data set of cases and controls and also strengthen previous published work by using depression measures from validated questionnaires. Second, within depression cases we test the role of antidepressant usage in accentuating an individual’s genetic risk for obesity. Finally, for the first time we perform negative control experiments to test whether any observed interactions are real or a consequence of the higher mean and SD in depression cases vs controls.

## Methods

### UK Biobank participants

UK Biobank recruited >500 000 participants from across the UK between 2006 and 2010 (https://www.ukbiobank.ac.uk/) and is described elsewhere.^11^ Briefly, participants provided detailed phenotypic data, blood and urine samples, and agreed to have their health followed over time. Genetic data were available for all participants and we defined 451 025 participants of European ancestry using principal component analysis as previously described. We also defined a subset of 378 214 unrelated individuals using a KING Kinship matrix excluding one individual for each pair of related individuals up to (and inclusive of) third-degree relatives.^12^

### Phenotypes

#### Depression

Depression was defined in two ways. The depression symptoms (DS; 41 389 cases and 246 065 controls) variable was defined from self-report and Hospital Episode Statistics using the whole UK Biobank baseline interview data as previously described.^4^ Second, major depression (MD) was defined in a subset of unrelated individuals (*N* = 123 923) who completed the mental health questionnaire (MHQ; 29 488 cases of MD and 94 363 controls) using the definition proposed by Davis *et al*.^13^ Details of these variables can be found in the Supplementary material (available as Supplementary data at *IJE* online). The MHQ is based on the Composite International Diagnostic Interview Short Form (CIDI-SF)^14^ and was used to also derive a continuous measure of severity of depression and, as such, should help to limit spurious findings from the interaction analyses.^7^

#### Antidepressant usage

A binary antidepressant treatment at baseline (not lifetime) variable was derived using the relevant treatment codes in UK Biobank (field 20003, http://biobank.ndph.ox.ac.uk/showcase/coding.cgi?id=4&nl=1). Briefly, we extracted 82 relevant codes across seven classes of antidepressant (Supplementary Table S1, available as Supplementary data at *IJE* online). The number of cases on each class of antidepressant can be found in Supplementary Table S2 (available as Supplementary data at *IJE* online).

#### BMI

Weight and height were measured for all participants and BMI calculated. BMI was inverse normalized prior to analyses to limit potential biases as a result of a skewed distribution, including issues with heteroscedasticity.^7^

#### TDI

The TDI is a composite measure of deprivation based on unemployment, non-home ownership, household overcrowding and non-car ownership, with negative scores representing low deprivation.

#### Genetic variants for BMI

As previously described,^4^ we selected 73/76 (Supplementary Table S3, available as Supplementary data at *IJE* online) SNPs associated with BMI at genome-wide significance in Locke *et al*.^15^ Three SNPs were excluded because they were known to be highly pleiotropic with multiple traits. We used SNPs published in Locke *et al*. to avoid winner’s curse.^16^ In Locke *et al*., the *FTO* variant is rs1558902 whereas in Rivera *et al*. it is rs9939609. rs1558902 is in strong linkage disequilibrium with rs9939609 (D'* *=* *1, *R*^2^* *=* *0.9335).

The 73 variants were extracted from imputation data and a BMI–GRS for each participant calculated. Each variant was recoded to represent the number of BMI-increasing alleles. A weighted score was then calculated (Equation 1) in which SNPn is the dosage and βn is the beta-value from Locke *et al*. prior to rescaling to reflect the number of BMI-increasing alleles (Equation 2):

(1) Weighted score =b1 × SNP1 +b2 × SNP2 + …bn × SNPn.

 

(2) $$\;\mathrm{GRS}=\;\frac{\mathrm{Weighted}\;\mathrm{score}\;}{\sum \beta }$$

### Negative control experiments

We tested whether putative interactions found in this study were specific to depression or an artefact of the different BMI distributions in depressed compared with non-depressed individuals. We used a computational optimization genetic algorithm for group (GAG) selection (url: https://github.com/drarwood/gags),^12^ which repeatedly sampled individuals to derive groups of the same number, matched to the BMI distribution of cases and controls but randomized to depression status. There was no overlap between individuals selected for the two groups. We repeated this random sampling 1000 times and the interaction *P*-values (described below) were calculated each time. We report the median analysis based on the interaction *P*-value.

### Statistical analysis

The mean and SD of BMI were calculated in depression cases and controls and within cases stratified by antidepressant usage.

For both depression measures, we calculated the association between the BMI–GRS and BMI separately in cases and controls using linear regression models. The models were adjusted for age, sex, assessment centre, TDI, five ancestry principal components and genotyping platform (two were used).

Interactions between the BMI–GRS and depression on BMI were tested by including the respective interaction terms in the models (i.e. interaction term* *=* *BMI–GRS* *×* *depression status). This was repeated excluding depression cases reporting antidepressant usage.

Interactions were also calculated between the BMI–GRS and antidepressant use by including the interaction terms BMI–GRS* *×* *antidepressant usage status in cases only. Interaction analyses were then repeated using the 73 BMI variants individually to determine whether interactions are driven by a subset of variants.

All interaction models were adjusted as specified above. To control for potential confounders, we performed a sensitivity analysis as suggested by Keller,^17^ which includes all covariate-by-depression and covariate-by-BMI–GRS interaction terms in the models.

All interaction models and negative experiments were run for all individuals and separately for males and females. All analyses were performed in Stata (version 16) or R (version 3.5.2).

## Results

Individuals with DS or MD had a higher BMI compared with controls with cases having 0.7 and 0.6 kg/m^2^ higher BMI, respectively (Table 1). This was the same for males and females (males: +0.6 kg/m^2^ for MD and +0.5 kg/m^2^ for DS; females +0.8 kg/m^2^ for MD and +0.7 kg/m^2^ for DS; Table 1).

**Table 1:** Comparison of body mass index between different groups in all participants (all) and stratified by sex: cases vs controls, cases on treatment vs cases not on treatment and severity score above median vs below median

Trait	Strata	*N*	*N* females (%)	**BMI [mean (SD)] All**	**BMI [mean (SD)] Females**	**BMI [mean (SD)] Males**	Effect size (95% CI)^a^	*P*-value
Major depression (MD)	Controls	94 363	49 248 (52.2)	26.6 (4.4)	26.1 (4.7)	27.2 (3.9)	Reference	
	Cases	29 488	20 290 (68.8)	27.2 (5.1)	26.9 (5.3)	27.8 (4.4)	0.79 (0.73; 0.85)	<2E-16
Major depression (MD)	Cases not on treatment	24 912	16 939 (68.0)	27.0 (4.9)	26.7 (5.1)	27.7 (4.4)	Reference	
	Cases on treatment	4576	3351 (73.2)	28.3 (5.7)	28.1 (6.0)	28.8 (4.7)	1.31 (1.16; 1.47)	<2E-16
Depression symptoms (DS)	Controls	246 065	118 374 (48.1)	27.2 (4.6)	26.7 (4.9)	27.8 (4.1)	Reference	
	Cases	41 389	26 217 (63.3)	27.9 (5.4)	27.8 (5.7)	28.2 (4.7)	0.82 (0.77; 0.87)	<2E-16
Depression symptoms (DS)	Cases not on treatment	30 835	19 044 (61.7)	27.6 (5.1)	27.3 (5.4)	28.0 (4.5)	Reference	
	Cases on treatment	10 554	7173 (68.0)	29.0 (6.0)	28.9 (6.3)	29.1 (5.1)	1.37 (1.25; 1.49)	<2E-16
Depression severity	Below median score (<3)	60 526	28 079 (46.4)	26.6 (4.2)	25.9 (4.5)	27.2 (3.8)	Reference	
	Above median score (≥3)	63 397	41 498 (65.5)	26.9 (4.8)	26.6 (5.1)	27.6 (4.2)	0.58 (0.52; 0.63)	<2E-16

a Effect size represents change in BMI (kg/m^2^) between the two groups. BMI, body mass index.

### Individuals with depression and those with more severe depression were associated with an accentuated risk of high BMI

**Figure 1:**
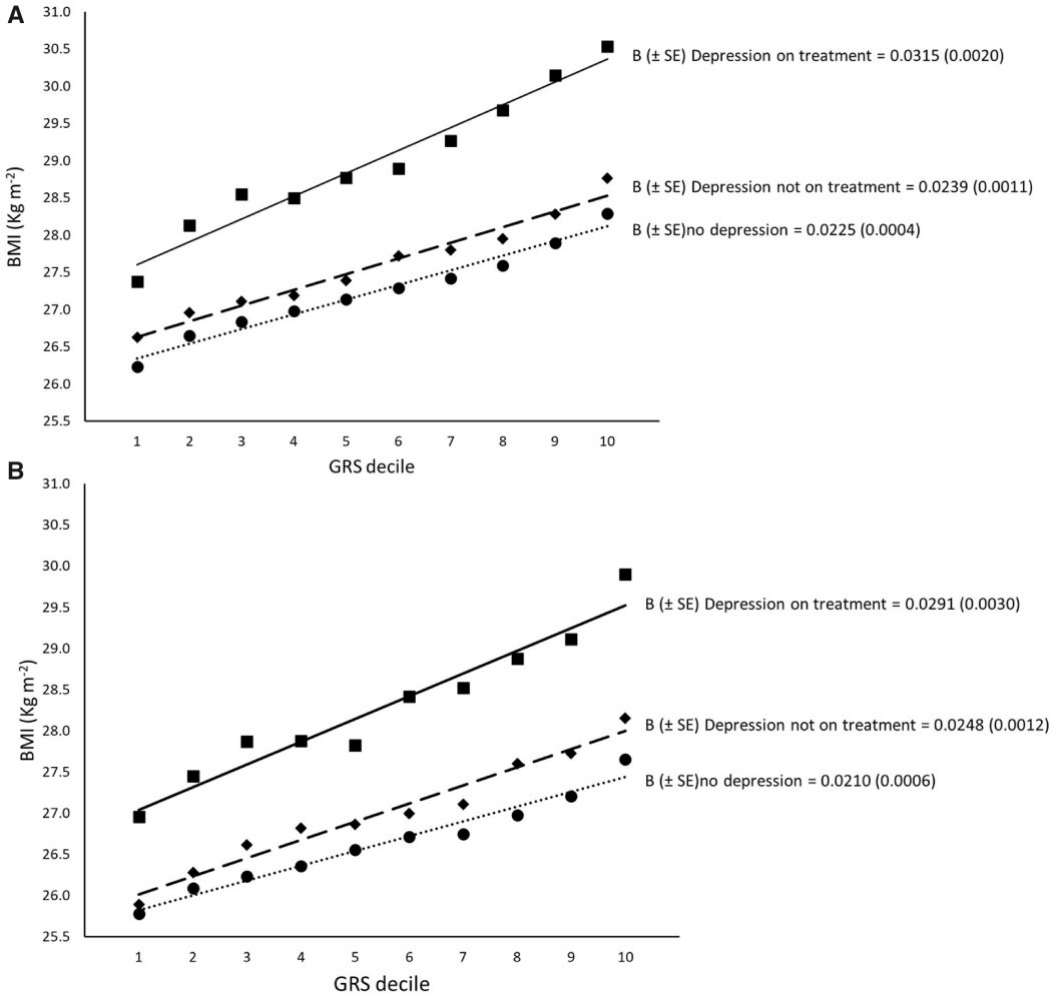
Association between the BMI–GRS (by decile) and BMI in participants without depression (black circlers and dotted line), participants with depression not on treatment (diamonds and dashed line) and participants with depression on treatment (squares and solid line) according to depression symptoms (DS, panel A) and major depression (MD, panel B) definitions BMI, body mass index; GRS, genetic risk score; B, beta-coefficient; kg, kilograms; m, metres.

We observed interactions between depression status and genetic susceptibility to high BMI using both depression definitions (Table 2 and Figure 1). This apparent gene-by-depression interaction meant that, compared with non-depressed individuals, individuals with DS had a 0.91-kg/m^2^ higher BMI if they had the highest BMI genetic risk (top decile) but a 0.58-kg/m^2^ higher BMI if they had the lowest BMI genetic risk (bottom decile, Table 2). Similarly, MD was associated with a 0.78-kg/m^2^ higher BMI in people with the highest genetic risk (top decile) but only a 0.27-kg/m^2^ higher BMI in people with the lowest genetic risk (bottom decile, Table 2). Another way of expressing this interaction is that carrying 10 additional BMI-raising alleles (weighted by effect size) was associated with 3.7 kg extra weight in the MD group and 3.0 kg in the non-depressed group, for someone 1.73 m tall.

**Table 2:** Differences in body mass index by BMI–GRS decile, association and interaction for the depression measures and stratified by treatment status

Trait	Strata	BMI difference in 10% lowest genetic risk	BMI difference in 10% highest genetic risk	Per allele beta	SE	*P*-value association	*P*-value interaction against reference	*N* negative tests below observed *P*
Major depression (MD)	Controls	Reference	Reference	0.0210	0.0006	<2E-16	Reference	
	Cases	+ 0.27 kg/m^2^	+ 0.78 kg/m^2^	0.0255	0.0011	<2E-16	6.98E-05	67/1000
	Cases not on treatment	+ 0.15 kg/m^2^	+ 0.53 kg/m^2^	0.0248	0.0012	<2E-16	1.32E-03	NA
	Case on treatment	+ 1.21 kg/m^2^	+ 2.28 kg/m^2^	0.0291	0.0030	<2E-16	1.36E-03	NA
Major depression (MD)	Cases not on treatment	Reference	Reference	0.0248	0.0012	<2E-16	Reference	
	Cases on treatment	+ 1.06 kg/m^2^	+ 1.75 kg/m^2^	0.0291	0.003	<2E-16	1.35E-01	510/1000
Depression symptoms (DS)	Controls	Reference	Reference	0.0225	0.0004	<2E-16	Reference	
	Cases	+ 0.58 kg/m^2^	+ 0.91 kg/m^2^	0.0257	0.0010	<2E-16	7.27E-04	Unable to do
	Cases not on treatment	+ 0.42 kg/m^2^	+ 0.49 kg/m^2^	0.0239	0.0011	<2E-16	1.58E-01	NA
	Case on treatment	+ 1.16 kg/m^2^	+ 2.27 kg/m^2^	0.0315	0.0020	<2E-16	1.40E-06	NA
Depression symptoms (DS)	Cases not on treatment	Reference	Reference	0.0239	0.0011	<2E-16	Reference	
	Cases on treatment	+ 0.74 kg/m^2^	+ 1.78 kg/m^2^	0.0315	0.0020	<2E-16	9.24E-04	162/1000
Depression severity	Below median score (<3)	Reference	Reference	0.0209	0.0007	<2E-16	Reference	
	Above median score (≥3)	+ 0.32 kg/m^2^	+ 0.59 kg/m^2^	0.0231	0.0007	<2E-16	4.18E-04	NA

For differences in BMI, all values calculated from reference. For regression, ‘per allele beta’ represents BMI increase (inverse normalized scale) per allele within each group. *N* negative tests below observed *P* is the number of negative experiments resulting in an interaction *P* smaller than the observed 1 out of 1000 negative experiments. BMI, body mass index; GRS, genetic risk score; SE, standard error.

There was strong evidence of an interaction using depression severity (*P_interaction_ *=* *4* *×* *10^–4^; Table 2 and Supplementary Figure S1, available as Supplementary data at *IJE* online). Individuals above the median score compared with individuals below the median score for depression severity had a 0.59-kg/m^2^ higher BMI in people with the highest genetic risk (top decile) but a 0.32-kg/m^2^ higher BMI in people with the lowest genetic risk (bottom decile, Table 2).

### Sensitivity analyses

Results were generally consistent when using the Keller method (Supplementary Table S4, available as Supplementary data at *IJE* online) and stratifying by sex (Table 3). The interaction observed for DS appears to be mostly driven by females whereas the depression severity interaction was driven by males (Table 3).

**Table 3:** Differences in body mass index by BMI–GRS decile, association and interaction for the depression measures and stratified by treatment status in females and males separately

Trait	Strata	FEMALES ONLY							MALES ONLY						
		**BMI difference in 10% lowest genetic risk**	**BMI difference in 10% highest genetic risk**	**Per allele beta**	**SE**	***P* association**	***P* interaction against reference**	***N* negative tests below observed *P***	**BMI difference in 10% lowest genetic risk**	**BMI difference in 10% highest genetic risk**	**Per allele beta**	**SE**	***P* association**	***P* interaction against reference**	***N* negative tests below observed *P***
Major depression (MD)	Controls	Reference	Reference	0.0216	0.0009	<2E-16	Reference		Reference	Reference	0.0200	0.0007	<2E-16	Reference	
	Cases	+ 0.56 kg/m^2^	+ 1.01 kg/m^2^	0.0255	0.0014	<2E-16	1.20E-02	340/1000	+ 0.24 kg/m^2^	+ 0.81 kg/m^2^	0.0256	0.0017	<2E-16	1.90E-03	66/1000
	Cases not on treatment	+ 0.45 kg/m^2^	+ 0.73 kg/m^2^	0.0245	0.0015	<2E-16	7.62E-02	NA	+ 0.08 kg/m^2^	+ 0.62 kg/m^2^	0.0257	0.0018	<2E-16	3.01E-03	NA
	Case on treatment	+ 1.43 kg/m^2^	+ 2.65 kg/m^2^	0.0308	0.0037	<2E-16	4.32E-03	NA	+ 1.63 kg/m^2^	+ 2.08 kg/m^2^	0.0243	0.0048	5.14E-07	3.54E-01	NA
Major depression (MD)	Cases not on treatment	Reference	Reference	0.0245	0.0015	<2E-16	Reference		Reference	Reference	0.0257	0.0018	<2E-16	Reference	
	Cases on treatment	+ 0.97 kg/m^2^	+ 1.91 kg/m^2^	0.0308	0.0037	<2E-16	7.34E-02	334/1000	+ 1.55 kg/m^2^	+ 1.46 kg/m^2^	0.0243	0.0048	5.14E-07	7.21E-01	700/1000
Depression symptoms (DS)	Controls	Reference	Reference	0.0233	0.0006	<2E-16	Reference		Reference	Reference	0.0217	0.0004	<2E-16	Reference	
	Cases	+ 0.86 kg/m^2^	+ 1.28 kg/m^2^	0.0269	0.0013	<2E-16	4.30E-03	576/1000	+ 0.55 kg/m^2^	+ 0.71 kg/m^2^	0.0234	0.0014	<2E-16	2.26E-01	856/1000
	Cases not on treatment	+ 0.67 kg/m^2^	+ 0.82 kg/m^2^	0.0250	0.0015	<2E-16	2.25E-01	NA	+ 0.38 kg/m^2^	+ 0.35 kg/m^2^	0.0223	0.0016	<2E-16	7.83E-01	NA
	Case on treatment	+ 1.47 kg/m^2^	+ 2.68 kg/m^2^	0.0330	0.0026	<2E-16	3.15E-05	NA	+ 1.25 kg/m^2^	+ 2.02 kg/m^2^	0.0276	0.0031	<2E-16	4.28E-02	NA
Depression symptoms (DS)	Cases not on treatment	Reference	Reference	0.0250	0.0015	<2E-16	Reference		Reference	Reference	0.0223	0.0016	<2E-16	Reference	
	Cases on treatment	+ 0.81 kg/m^2^	+ 1.87 kg/m^2^	0.0330	0.0026	<2E-16	5.41E-03	119/1000	+ 0.87 kg/m^2^	+ 1.67 kg/m^2^	0.0276	0.0031	<2E-16	1.41E-01	265/1000
Depression severity	Below median score (<3)	Reference	Reference	0.0225	0.0011	<2E-16	Reference		Reference	Reference	0.0194	0.0009	<2E-16	Reference	
	Above median score (≥3)	+ 0.68 kg/m^2^	+ 0.88 kg/m^2^	0.0230	0.0010	<2E-16	1.97E-01	NA	+ 0.30 kg/m^2^	+ 0.64 kg/m^2^	0.0232	0.0011	<2E-16	6.30E-05	NA

For differences in BMI, all values calculated from reference. For regression, ‘per allele beta’ represents BMI increase (inverse normalized scale) per allele within each group. BMI, body mass index; GRS, genetic risk score; SE, standard error.

### Antidepressant medication usage was associated with an accentuated risk of high BMI

We then tested whether antidepressant usage within depression cases accentuated an individual’s genetic risk of obesity when compared with cases not taking antidepressants. Within DS cases, antidepressant treatment accentuated an individual’s genetic risk of obesity (Table 2 and Figure 1, *P*_interaction_* *=* *0.0009). Similar effect sizes were noted for MD cases (Table 2) but confidence intervals crossed the null (*P*_interaction_* *=* *0.14). Depression cases on antidepressants had an ∼1.4-kg/m^2^ higher BMI than people who reported depression but were not treated with antidepressants using both depression definitions (Table 1).

In depressed cases not on treatment, we observed an attenuation of the interaction effect but some evidence of an interaction remained. DS cases (without antidepressant medication) were associated with a 0.49-kg/m^2^ higher BMI in people with the highest genetic risk (top decile) but a 0.42-kg/m^2^ higher BMI in people at lowest genetic risk (bottom decile) when compared with non-depressed individuals (*P*_interaction_* *=* *0.16). MD cases (without antidepressant medication) were associated with a 0.53-kg/m^2^ higher BMI in people with the highest genetic risk (top decile) but a 0.15-kg/m^2^ higher BMI in people at lowest genetic risk (bottom decile) when compared with non-depressed individuals (*P*_interaction_* *=* *0.001; Figure 1).

To test whether antidepressant use by BMI–GRS interaction is explained by depression severity (i.e. more severe cases are on treatment, less severe cases are not), we repeated our analyses adding a BMI–GRS by depression severity interaction term to the model. Adding this new interaction attenuated the BMI–GRS by treatment interaction to the null (beta interaction = 0.004, *P *=* *0.32 with severity interaction term vs 0.007, *P *=* *9.2E-04 without severity interaction term).

### Sensitivity analyses

Antidepressant usage interactions were generally consistent when using the Keller method (Supplementary Table S4, available as Supplementary data at *IJE* online). Sex-stratified analyses provided evidence of interactions when comparing females on antidepressants to controls but no evidence in males (Table 3). Interaction was observed in males only when comparing cases on antidepressant medication to controls (Table 3).

### Negative control experiments provide evidence that BMI distribution contributes to some of the observed interactions

**Figure 2:**
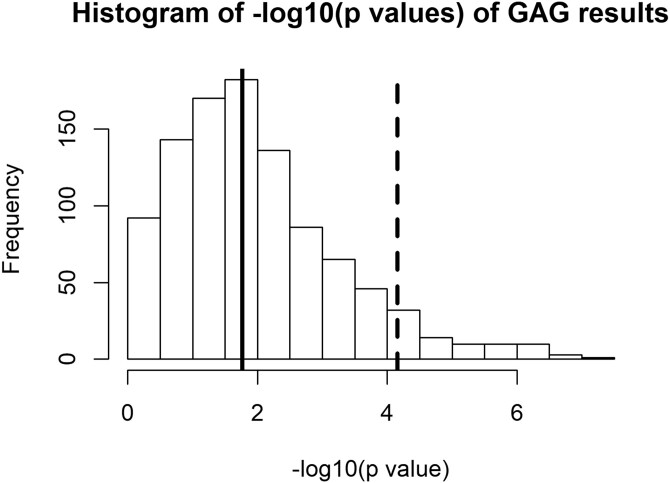
Histogram of the –log10 *P*-values obtained from 1000 negative-experiment major depression (MD) by BMI–GRS interaction analysis when we randomly created groups of individuals to have the same means and standard deviations of participants with and without depression. Dashed vertical lines represent the observed *P*-value in UK Biobank and the solid vertical line represents the median –log10p of the negative experiments. GAG, genetic algorithm for group selection.

We tested whether observed interactions were real and not a consequence of selecting groups of individuals with a higher mean and SD of BMI and comparing them to groups of individuals with a lower mean and SD of BMI by sampling individuals 1000 times to have identical BMI distributions (means and SD) to the depression cases and controls but randomized to depression status. For MD, 67/1000 analyses (Table 2, P = 0.067) demonstrated stronger interactions than observed with the real variable, providing inconclusive evidence for the validity of the observed interactions. The median interaction *P*-value obtained was 0.017, whilst the real interaction was 7* *×* *10^–5^ (Figure 2).

For DS, our algorithm was unable to recreate the distributions due to the large sample sizes in both groups and therefore we were unable to run the negative experiments.

Negative control experiments for the antidepressant interaction within depressed cases suggested that our observed interaction might be a consequence of the BMI distributions and/or confounded by unaccounted for variables. For DS, 162/1000 random samples demonstrated stronger interactions than observed with the real antidepressant usage variable, with a median *P*-value of 0.022 whilst the real interaction was 9* *×* *10^–4^ (Table 2 and Supplementary Figure S2, available as Supplementary data at *IJE* online). For MD, 510/1000 simulations demonstrated a stronger interaction than observed with the real variable, with a median *P*-value similar to that obtained with the real variable (0.1355 vs 0.1295; Table 2 and Supplementary Figure S3, available as Supplementary data at *IJE* online).

Negative experiments were also performed for sex-stratified analyses, providing inconclusive evidence about the interactions observed with MD in males only (Table 3, empirical-*P *=* *0.066) and suggested that statistical artefacts drive the interaction in females only (Table 3 and Supplementary Figures S4 and S5, available as Supplementary data at *IJE* online). For DS, the observed interactions in males and females may be due to statistical artefacts (Table 3 and Supplementary Figures S6 and S7, available as Supplementary data at *IJE* online) suggesting that if we were able to recreate the distribution in all individuals, our results would indicate that observed interactions may not be real. Negative experiments for the sex-stratified antidepressant medication analyses provided further evidence of the importance of the BMI distribution in these interaction analyses (Table 3 and Supplementary Figures S8–S11, available as Supplementary data at *IJE* online).

### Interactions with individual BMI variants

Several of the 73 individual BMI SNPs demonstrated nominal interaction effects with DS (*n* = 4) and MD (*n* = 8) at *P *<* *0.05 (Table 4, Supplementary Table S5 and Supplementary Figures S12 and S13, available as Supplementary data at *IJE* online). Only one variant, rs10182181 near the *ADCY3* gene, survived Bonferroni correction (*P_interaction_ = *3* *×* *10^–5^) using the MD definition. To check that SNP interactions were not driven by their strength of association with BMI, we plotted the main effect from Locke *et al.* (2014) and the interaction effect for each SNP (Supplementary Figure S14, available as Supplementary data at *IJE* online) and found weak correlations (DS Pearson *r* = 0.263, *P *=* *0.025; MD Pearson *r* = 0.365, *P *=* *0.015).

**Table 4:** Details of association between single-nucleotide polymorphism (SNP) and body mass index in cases vs controls for SNPs with a nominally significant SNP by depression interaction

Depression measure	Case/control	SNP	Beta (SE)	*P*-value	*P*-value interaction	LOCUS
Major depression (MD)	Controls	rs10182181	0.0270 (0.0043)	4.88E-10	2.85E-05	*ADCY3*
	Cases	rs10182181	0.0623 (0.0085)	5.64E-14		*ADCY3*
Major depression (MD)	Controls	rs9925964	0.0129 (0.0045)	4.51E-03	5.70E-03	*KAT8*
	Cases	rs9925964	0.0381 (0.0089)	1.99E-05		*KAT8*
Major depression (MD)	Controls	rs13021737	0.0454 (0.0057)	2.45E-15	8.93E-03	*TMEM18*
	Cases	rs13021737	0.0761 (0.0113)	1.72E-11		*TMEM18*
Major depression (MD)	Controls	rs17724992	0.0086 (0.0049)	7.90E-02	1.60E-02	*PGPEP1*
	Cases	rs17724992	0.0327 (0.0097)	7.30E-04		*PGPEP1*
Major depression (MD)	Controls	rs17094222	0.0068 (0.0053)	1.97E-01	2.62E-02	*HIF1AN*
	Cases	rs17094222	0.0312 (0.0104)	2.72E-03		*HIF1AN*
Major depression (MD)	Controls	rs3810291	0.0175 (0.0046)	1.64E-04	4.46E-02	*ZC3H4*
	Cases	rs3810291	0.0367 (0.0092)	6.24E-05		*ZC3H4*
Major depression (MD)	Controls	rs17024393	0.0562 (0.0138)	4.79E-05	4.53E-02	*GNAT2*
	Cases	rs17024393	0.0113 (0.0270)	2.95E-05		*GNAT2*
Major depression (MD)	Controls	rs17405819	0.0133 (0.0047)	4.79E-03	4.56E-02	*HNF4G*
	Cases	rs17405819	0.0338 (0.0094)	3.40E-04		*HNF4G*
Depression symptoms (DS)	Controls	rs6567160	0.0449 (0.0032)	2.04E-44	2.72E-03	*MC4R*
	Cases	rs6567160	0.0707 (0.0087)	5.29E-16		*MC4R*
Depression symptoms (DS)	Controls	rs2287019	0.0305 (0.0035)	6.74E-18	1.21E-02	*QPCTL*
	Cases	rs2287019	0.0548 (0.0096)	1.10E-08		*QPCTL*
Depression symptoms (DS)	Controls	rs1808579	0.0176 (0.0027)	9.76E-11	4.53E-02	*NPC1, RMC1*
	Cases	rs1808579	0.0318 (0.0073)	1.54E-05		*NPC1, RMC1*
Depression symptoms (DS)	Controls	rs10733682	0.0122 (0.0028)	9.95E-06	4.65E-02	*LMX1B*
	Cases	rs10733682	0.0260 (0.0075)	5.62E-04		*LMX1B*

BMI, body mass index; SE, standard error.

We did not find evidence that *FTO* variants interact with depression to accentuate obesity risk (Supplementary Table S5 and Supplementary Figures S15 and S16, available as Supplementary data at *IJE* online). For *FTO*, we ran negative experiments: with MD, 726/1000 analyses demonstrated stronger interactions than observed with the real variable (Supplementary Figure S15, available as Supplementary data at *IJE* online). We also tested the *FTO* interaction reported in Rivera *et al.*^6^ by creating random distributions using the number per group and means reported in the article. Here, 35 interactions were below the random-effect *P*-value in Rivera *et al.* (0.027) but none (Supplementary Figure S16, available as Supplementary data at *IJE* online) was below the Han/Eskin random-effect *P*-value (Han/Eskin *P *=* *7* *×* *10^–8^).^6^ For the latter negative experiments, the median interaction *P*-value obtained for these 1000 analyses was 0.475 (range 9.4E-05–0.997).

Individual BMI variants nominally (*P *<* *0.05) interacted with antidepressant usage (Supplementary Table S6 and S7, available as Supplementary data at *IJE* online) but no SNPs survived Bonferroni correction.

## Discussion

In UK Biobank, we provided tentative evidence that depression status accentuates an individual’s genetic susceptibility to higher BMI but this could be driven by differences in distributions, at least for some of the definitions of depression analysed here. Our results also suggest that current antidepressant usage in the depression cases might accentuate an individual’s genetic susceptibility to higher BMI but our negative experiments suggest that this interaction might be a statistical artefact. Our results highlight the crucial importance of negative control experiments when running gene-by-environment analyses, which are prone to confounding and various biases.^7^

Here, negative control experiments mimicked the BMI distribution of depression cases and controls, testing whether observed interactions were in part driven by statistical biases induced by the higher mean and SD of BMI in depression cases.^7^ These negative control experiments suggested that the MD by BMI–GRS interactions in all participants might not be solely driven by statistical biases arising from the different BMI distributions. Here, we observed a stronger interaction in 6.7% of our negative control experiments, which is more than the ideal <5% threshold. However, GAG can only approximate means and SD to one decimal point so a better approximation of the distributions might lower the number of negative control experiments demonstrating a stronger interaction than that achieved with the real variables. Because of this, we cannot conclude with confidence whether this observed interaction is real or the result of statistical artefacts. The sex-stratified negative experiments demonstrated similar results in males but suggested that the interaction observed in females may be driven by statistical artefacts due to the different BMI distributions. The GAG algorithm was unable to confirm the DS results in all participants and to recreate the distributions used in Mulugeta *et al.* due to the large number of individuals in the control group. Our DS results were in agreement with those from Mulugeta *et al*. using a similar definition of depression, but the lack of negative experiments in all individuals means that we are unable to fully exclude the role of BMI distributions in these interactions. However, in the sex-stratified negative experiments, our findings suggest that the BMI distributions might drive the observed interactions, casting doubt on the interactions observed in all individuals.

Our analyses suggested that being on antidepressant medication accentuates an individual’s genetic susceptibility to higher BMI, especially in females. However, these findings should be taken with great caution and need to be confirmed by further investigations because interaction results were not demonstrated using the MD definition and negative experiments suggest that we cannot exclude the role of differences in the BMI distribution or unmeasured confounders driving these antidepressant interactions. An antidepressant–obesity interaction, if real, would fit with previous studies highlighting weight gain during antidepressant treatment.^9^^,^^10^

Several BMI variants demonstrated a nominal interaction with MD, with all variants demonstrating a stronger effect on BMI in people with depression. The strongest interaction was for rs10182181 in *ADCY3*, which has previously been implicated in depression development in mice^18^ and is reported to affect the response to different diet regimes in humans.^19^

In UK Biobank we were unable to replicate the previously reported interaction between a SNP in the *FTO* gene and depression.^6^ The *FTO* SNP reported in Locke *et al*. is different from the variant used by Rivera *et al*. but, here, neither showed an interaction with depression. This may be explained by the different depression definitions, with a more severe clinical definition used in Rivera *et al*. Another possible explanation could be that the observed interaction with the *FTO* gene may have been driven by the differential BMI distributions in depression cases and controls. This is supported by our negative experiments, but not when using the distributions reported in Rivera *et al*.

Our analysis had several strengths. UK Biobank provides a single large study with a homogenous depression definition unlike meta-analysis studies that can be limited by non-homogeneous definitions. The size of UK Biobank facilitates the negative-controls experiments, allowing us to test the robustness of our results by determining whether statistical artefacts including heteroscedasticity drive the observed interaction, which has not been considered previously for the obesity gene–depression interactions. The negative experiments account for statistical artefacts that may bias gene–depression interactions and enable us to test the specificity of our interaction. For example, people may be overweight for many reasons other than depression (e.g. social deprivation) and if this were the case, we would expect to see similar interactions in any group who were of similar BMI.

This study has a number of limitations. First, our negative-experiment algorithm could only recreate the BMI distribution to one decimal point, meaning the distributions maintain a margin of error. Second, the heterogeneity of depression and data availability in UK Biobank means that our results might not be applicable to specific depression subtypes. Third, the MHQ used to create the MD definition was not performed at baseline and therefore there is a time gap between BMI measurements and the MD definitions (median time between baseline and MHQ = 7.6 years, range 5.9–10.8 years). It is not known whether BMI at the time of completing the MHQ was similar to the BMI at baseline, but BMI was measured twice in a subgroup of 34 168 participants and the average change was 0.01 (± 1.9) kg/m^2^. UK Biobank data are not population representative, with studies demonstrating volunteer bias^20^ and participation bias.^21^^,^^22^ Fourth, the MHQ was only completed in a subset of individuals, which may introduce further biases;^21^ however, we did observe similar effect estimates from a broader measure of depression from the baseline UK Biobank. Finally, there is the possibility that depression interaction analyses could be subject to confounding by unmeasured variables, as with other observational analyses. We included key covariates and adjusted our models for the gene-covariate and depression-covariate interaction terms, but we cannot rule out other confounders partially explaining the reported interaction.

In conclusion, we have demonstrated that, in UK Biobank, depression observationally accentuates an individual’s genetic risk of obesity but we showed that this appears to be driven by differences in BMI distributions between cases and controls. We have, in fact, highlighted the importance of testing putative gene-by-environment interactions as clearly demonstrated by the results of our negative experiments.

## Ethics approval

UK Biobank received ethical approval from the North West Multi-Centre Research Ethics Committee (REC reference: 11/NW/03820). All participants gave written informed consent in accordance with the principles of the Declaration of Helsinki.

## Data availability

All data from UK Biobank are publically available; the negative experiments algorithm can be found here https://github.com/drarwood/gags.

## Supplementary data

Supplementary data are available at *IJE* online.

## Author contributions

F.C. and J.T. designed the study and wrote the manuscript. F.C., J.O., C.L., T.M.F., A.R.W. and J.T. were involved in data processing, statistical analyses and interpretation. J.T. is the guarantor. All authors assisted in the writing, reviewing and approval of the manuscript.

## Funding

J.O., F.C. and J.T. are supported by an Academy of Medical Sciences (AMS) Springboard award, which is supported by the AMS, the Wellcome Trust, GCRF, the Government Department of Business, Energy and Industrial Strategy, the British Heart Foundation and Diabetes UK (SBF004\1079). A.R.W. and T.M.F. are supported by the European Research Council grant SZ-245 50371-GLUCOSEGENES-FP7-IDEAS-ERC. The funders had no role in the study design, analysis or interpretation. All authors confirm their independence from the funders and confirm that they had full access to all the data and can take responsibility for the integrity of the data and accuracy of the data analysis. C.M.L. is part-funded by the National Institute for Health Research (NIHR) Biomedical Research Centre at South London and Maudsley NHS Foundation Trust and King’s College London. The views expressed are those of the authors and not necessarily those of the NHS, the NIHR or the Department of Health and Social Care.

## Supplementary Material

dyac052_Supplementary_Data
