# Supplementary material for: Simulated distributions from negative experiments highlight the importance of the body mass index distribution in explaining depression–body mass index genetic risk score interactions
Source: Int J Epidemiol. 2022 Apr 7;51(5):1581–92. doi: 10.1093/ije/dyac052 (PMC9557895; doi:10.1093/ije/dyac052)
Supplement: dyac052_Supplementary_Data [file dyac052_supplementary_data.docx]

**Supplementary material captions.**

**Supplementary methods:** Depression phenotype definitions.

**Supplementary Table S1.** Antidepressant drug names, codes from UK Biobank, field 20003, http://biobank.ndph.ox.ac.uk/showcase/coding.cgi?id=4&nl=1 and antidepressant class.

**Supplementary Table S2**. Number of depression cases on treatment for each class of antidepressant

**Supplementary Table S3**. Summary of the genetic variants for BMI from Locke et al.

**Supplementary Table S4**: Results of the sensitivity analysis using the model adjustment suggested by Keller et al. (2014). Data are presented as in main results with adjustment for covariates by depression and covariates by genetic risk score interaction terms.

**Supplementary Table S5**. Results of association and interaction analyses between individual BMI SNPs and BMI split by depression cases and controls for all 73 SNPs investigated.

**Supplementary Table S6.** Details of association between SNP and BMI in cases on treatment vs cases not on treatment for SNPs with a nominally significant SNP by depression treatment interaction.

**Supplementary Table S7**. Results of association and interaction between SNP and BMI split by depression cases on treatment vs cases not on treatment (depression symptoms (DS) definition) for all 73 SNPs investigated.

**Supplementary figure S1**. Association between the BMI GRS (by decile) and BMI in participants above (black triangle and solid line) and below (circles and dashed line) the median value for severity of depression (median=3).

**Supplementary figure S2**. Histogram of the –log10 p values obtained from 1000 negative experiment treatment of depression by BMI GRS interaction analysis using depression symptoms definition (DS) when we randomly created groups of individuals to have the same means and standard deviations of participants with depression on treatment and not on treatment. Red vertical lines represent the observed P-value in UK Biobank and the black line represents the median -log10p of the negative experiments.

**Supplementary figure S3**. Histogram of the –log10 p values obtained from 1000 negative experiment treatment of depression by BMI GRS interaction analysis using major depression definition (MD) when we randomly created groups of individuals to have the same means and standard deviations of participants with depression on treatment and not on treatment. Red vertical lines represent the observed P-value in UK Biobank and the black line represents the median -log10p of the negative experiments.

**Supplementary figure S4**. Histogram of the –log10 p values obtained from 1000 negative experiment treatment of depression by BMI GRS interaction analysis using major depression definition (MD) in males only when we randomly created groups of individuals to have the same means and standard deviations of participants with and without depression. Red vertical lines represent the observed P-value in UK Biobank and the black line represents the median -log10p of the negative experiments.

**Supplementary figure S5**. Histogram of the –log10 p values obtained from 1000 negative experiment treatment of depression by BMI GRS interaction analysis using major depression definition (MD) in females only when we randomly created groups of individuals to have the same means and standard deviations of participants with and without depression. Red vertical lines represent the observed P-value in UK Biobank and the black line represents the median -log10p of the negative experiments.

**Supplementary figure S6**. Histogram of the –log10 p values obtained from 1000 negative experiment treatment of depression by BMI GRS interaction analysis using depression symptoms definition (DS) in males only when we randomly created groups of individuals to have the same means and standard deviations of participants with and without depression. Red vertical lines represent the observed P-value in UK Biobank and the black line represents the median -log10p of the negative experiments.

**Supplementary figure S7**. Histogram of the –log10 p values obtained from 1000 negative experiment treatment of depression by BMI GRS interaction analysis using depression symptoms definition (DS) in females only when we randomly created groups of individuals to have the same means and standard deviations of participants with and without depression. Red vertical lines represent the observed P-value in UK Biobank and the black line represents the median -log10p of the negative experiments.

**Supplementary figure S8**. Histogram of the –log10 p values obtained from 1000 negative experiment treatment of depression by BMI GRS interaction analysis using major depression definition (MD) in males when we randomly created groups of individuals to have the same means and standard deviations of participants with depression on treatment and not on treatment. Red vertical lines represent the observed P-value in UK Biobank and the black line represents the median -log10p of the negative experiments.

**Supplementary figure S9**. Histogram of the –log10 p values obtained from 1000 negative experiment treatment of depression by BMI GRS interaction analysis using major depression definition (MD) in females when we randomly created groups of individuals to have the same means and standard deviations of participants with depression on treatment and not on treatment. Red vertical lines represent the observed P-value in UK Biobank and the black line represents the median -log10p of the negative experiments.

**Supplementary figure S10**. Histogram of the –log10 p values obtained from 1000 negative experiment treatment of depression by BMI GRS interaction analysis using depression symptoms definition (DS) in males when we randomly created groups of individuals to have the same means and standard deviations of participants with depression on treatment and not on treatment. Red vertical lines represent the observed P-value in UK Biobank and the black line represents the median -log10p of the negative experiments.

**Supplementary figure S11**. Histogram of the –log10 p values obtained from 1000 negative experiment treatment of depression by BMI GRS interaction analysis using depression symptoms definition (DS) in females when we randomly created groups of individuals to have the same means and standard deviations of participants with depression on treatment and not on treatment. Red vertical lines represent the observed P-value in UK Biobank and the black line represents the median -log10p of the negative experiments.

**Supplementary figure S12.** Forest plot of top 9 SNPs with strongest major depression (MD) by BMI GRS interaction and FTO SNP (rs1558902) stratified by participants status (cases vs controls). SNPs ordered by p for interaction top to bottom and left panel to right panel. * near the locus name represent SNPs with significant interaction term. Points represent standard deviation change in BMI per unit increasing allele, whiskers represent 95% confidence intervals.

**Supplementary figure S13.** Forest plot of top 9 SNPs with strongest depression symptoms (DS) by BMI GRS interaction and FTO SNP (rs1558902) stratified by participants status (cases vs controls). SNPs ordered by p for interaction top to bottom and left panel to right panel. * near the locus name represent SNPs with significant interaction term. Points represent standard deviation change in BMI per unit increasing allele, whiskers represent 95% confidence intervals.

**Supplementary figure S14.** Scatter plot of BMI SNPs main effect from Locke et al. (x axis) against interaction effect from our analysis (y axis). Results from depression symptoms (DS) by BMI GRS interaction (panel A) and from major depression (MD) and by BMI GRS interaction (panel B).

**Supplementary figure S15**. Histogram of the –log10 p values obtained from 1000 negative experiment of depression by FTO SNP (rs1558902) interaction analysis using major depression definition (MD) when we randomly created groups of individuals to have the same means and standard deviations of participants with and without depression. Red vertical lines represent the observed P-value in UK Biobank and the black line represents the median -log10p of the negative experiments.

**Supplementary figure S16**. Histogram of the –log10 p values obtained from 1000 negative experiment treatment of depression by FTO SNP (rs1558902) interaction analysis using distributions from Rivera et al. when we randomly created groups of individuals to have the same means and standard deviations of participants with and without depression. Red vertical lines represent the observed P-value in UK Biobank and the black line represents the median -log10p of the negative experiments.

**Supplementary methods**

*Depression phenotype definitions:*

Briefly, for DS individuals were considered a case if they met one or more of the following criteria:

• self-reported seeing a GP for nerves/anxiety or depression AND reported at least a 2-week duration of depression or unenthusiasm;

• self-reported seeing a psychiatrist for nerves/anxiety or depression AND reported at least a 2-week duration of depression or unenthusiasm;

• had the following ICD-10 codes in the Hospital Episode Statistics: F33 representing recurrent major depressive disorder (MDD) or F32 representing single-episode MDD.

Using this definition, we defined 41,389 DS cases and 246,065 controls (who had no reported depressive symptoms).

In the subset of unrelated individuals (N = 123,923) with the MHQ data available we defined MD using the well-established and validated Composite International Diagnostic Interview Short Form (CIDI-SF), as previously described by Davis et al. Briefly, we created a severity of depression variable using 8 variables. In each case (unless specifically stated) the options were Yes (score of 1) or No (score of 0):

• Have you ever had a time in your life when you felt sad, blue, or depressed for two weeks or more in a row? (data field 20446)

• Have you ever had a time in your life lasting two weeks or more when you lost interest in most things like hobbies, work, or activities that usually give you pleasure? (data field 20441)

• Did you feel more tired out or low on energy than is usual for you? (data field 20449)

• Did you gain or lose weight without trying, or did you stay about the same weight? (data field 20536). Here any response other than stayed the same, resulted in adding one to the overall CIDI-SF response variable.

• Did your sleep change? (data field 20532)

• Was that: [re sleep change] Waking too early? (data field 20535)

• Did you have a lot more trouble concentrating than usual? (data field 20435)

• Did you think a lot about death - either your own, someone else's or death in general? (data field 20437)

This severity score was then utilised to create a binary MD variable, where cases were defined based on the following criteria:

• Responded yes to the question: "Have you ever had a time in your life when you felt sad, blue, or depressed for two weeks or more in a row?" (data field 20446) and responded most of the day or all day to data field 20436 (Fraction of the day affected during worst episode of depression) or almost every day or every day to data field 20439 (Frequency of depressed days during worst episode of depression) or 1 somewhat or a lot to data field 20440 (Impact on normal roles during worst period of depression).

• Scored >4 in our derived CIDI response variable.

MD controls were defined from the subset who had undertaken the MHQ and who:

• Responded no to the question: "Have you ever had a time in your life when you felt sad, blue, or depressed for two weeks or more in a row?"

AND

• No self-reported depression and anxiety

AND

• No record of depression in hospital episode data

AND

• No known use of antidepressants

Using the MHQ CIDI-SF definition we defined 29,488 cases of MD and 94,363 controls.

In the subset of individuals with MHQ data 56.9% of the individuals reporting DS were defined as having MD.

We also used the CIDI-SF derived severity of depression measure as another measure of depression status in the interaction models (Supplementary Fig 17). This measure was continuous and as such should help to limit spurious findings from the interaction analyses

**Supplementary Table S1:** Antidepressant drug names, codes from UK Biobank, field 20003, http://biobank.ndph.ox.ac.uk/showcase/coding.cgi?id=4&nl=1 and antidepressant class.

| **Antidepressant** | **UK Biobank code** | **Class** |
| --- | --- | --- |
| faverin | 1140867860 | SSRI |
| prozac | 1140867876 | SSRI |
| sertraline | 1140867878 | SSRI |
| lustral | 1140867884 | SSRI |
| paroxetine | 1140867888 | SSRI |
| fluoxetine | 1140879540 | SSRI |
| fluvoxamine | 1140879544 | SSRI |
| seroxat | 1140882236 | SSRI |
| citalopram | 1140921600 | SSRI |
| cipramil | 1141151946 | SSRI |
| escitalopram | 1141180212 | SSRI |
| cipralex | 1141190158 | SSRI |
| prothiaden | 1140867624 | TCA |
| doxepin | 1140867640 | TCA |
| tryptizol | 1140867668 | TCA |
| anafranil | 1140867690 | TCA |
| lofepramine | 1140867726 | TCA |
| trimipramine | 1140867756 | TCA |
| surmontil | 1140867758 | TCA |
| ludiomil | 1140867784 | TCA |
| norval | 1140867812 | TCA |
| nortriptyline | 1140867818 | TCA |
| triptafen | 1140867934 | TCA |
| amitriptyline + chlordiazepoxide | 1140867938 | TCA |
| fluphenazine hydrochloride+nortriptyline | 1140867940 | TCA |
| amitriptyline hydrochloride+perphenazine | 1140867948 | TCA |
| amitriptyline | 1140879616 | TCA |
| clomipramine | 1140879620 | TCA |
| dothiepin | 1140879628 | TCA |
| imipramine | 1140879630 | TCA |
| sinequan | 1140882312 | TCA |
| dosulepin | 1140909806 | TCA |
| allegron | 1140867820 | TCA |
| fluphenazine hcl+nortriptyline | 1140867942 | TCA |
| elavil | 1140867658 | TCA |
| maproptiline | 1140879552 | TCA |
| tofranil | 1140867712 | TCA |
| desipramine | 1140879624 | TCA |
| protriptyline | 1140879632 | TCA |
| butriptyline | 1140856074 | TCA |
| evadyne | 1140856076 | TCA |
| praminil | 1140856144 | TCA |
| lomont | 1141146062 | TCA |
| gamanil | 1140882310 | TCA |
| aventyl | 1140867824 | TCA |
| amoxapine | 1140867774 | TCA |
| iprindole | 1140867720 | TCA |
| prondol | 1140867722 | TCA |
| phenelzine | 1140867850 | MAOI |
| nardil | 1140867852 | MAOI |
| isocarboxazid | 1140867856 | MAOI |
| tranylcypromine | 1140867914 | MAOI |
| moclobemide | 1140867920 | MAOI |
| manerix | 1140867922 | MAOI |
| maoi - tranylcypromine | 1140910820 | MAOI |
| tranylcypromine+trifluoperazine | 1140867944 | MAOI |
| maoi - phenelzine | 1140910704 | MAOI |
| maoi - isocarboxazid | 1140910504 | MAOI |
| marplan | 1140867858 | MAOI |
| parnate | 1140867916 | MAOI |
| selegiline | 1140879668 | MAOI |
| eldepryl | 1140872348 | MAOI |
| zelapar | 1141169666 | MAOI |
| mianserin | 1140879556 | NASSA |
| mirtazapine | 1141152732 | NASSA |
| zispin | 1141152736 | NASSA |
| bolvidon | 1140867806 | NASSA |
| venlafaxine | 1140916282 | SNRI |
| duloxetine | 1141200564 | SNRI |
| yentreve | 1141200570 | SNRI |
| cymbalta | 1141201834 | SNRI |
| edroanx | 1141151982 | SNRI |
| reboxetine | 1141151978 | SNRI |
| efexor | 1140916288 | SNRI |
| trazodone | 1140879634 | SARI |
| molipaxin | 1140882244 | SARI |
| nefazodone | 1140917460 | SARI |
| dutonin | 1140917466 | SARI |
| Bupropion | 1141176854 | NDRI |
| Methylphenidate | 1140917132 | NDRI |
| ritalin | 1140917138 | NDRI |
| concerta | 1141179874 | NDRI |

SSRIs= selective serotonergic reuptake inhibitors, TCA= tricyclic antidepressants, SNRI= serotonin-norepinephrine reuptake inhibitors, NASSA= noradrenergic and specific serotonergic antidepressant, MOAI= monoamine oxidase inhibitors, SARI= serotonin antagonists and reuptake inhibitors and NDRI= norepinephrine-dopamine reuptake inhibitors.

**Supplementary Table S2.** Number of depression cases on treatment for each class of antidepressant used in this study.

|  | **DS** |  | **MD** |  |
| --- | --- | --- | --- | --- |
|  | **N cases on treatment class** | **N cases not on treatment class** | **N cases on treatment class** | **N cases not on treatment class** |
| SSRI | 6739 | 34650 | 2910 | 26578 |
| TCA | 2550 | 38839 | 1125 | 28363 |
| MAOI | 41 | 41348 | 18 | 29470 |
| NASSA | 554 | 40835 | 193 | 29295 |
| SNRI | 1072 | 40317 | 437 | 29051 |
| SARI | 200 | 41189 | 80 | 29408 |
| NDRI | 6 | 41383 | 6 | 29482 |

**Supplementary Table S3**: Summary of the genetic variants for BMI from Locke et al.

| **Trait** | **Genetic variant** | **Locus** | **Exclude from score** | **Reason for exclusion** | **Trait raising allele** | **Trait lowering allele** | **Beta representing SD change in BMI from the primary GWAS** | **BMI SNP classification as neuoronal or non-neuronal** |
| --- | --- | --- | --- | --- | --- | --- | --- | --- |
| BMI | rs1000940 | *RABEP1* | No | NA | G | A | 0.019 | Non-neuronal |
| BMI | rs10132280 | *STXBP6* | No | NA | C | A | 0.023 | Neuronal |
| BMI | rs1016287 | *FLJ30838* | No | NA | T | C | 0.023 | Non-neuronal |
| BMI | rs10182181 | *ADCY3* | No | NA | G | A | 0.031 | Neuronal |
| BMI | rs10733682 | *LMX1B* | No | NA | A | G | 0.017 | Neuronal |
| BMI | rs10938397 | *GNPDA2* | No | NA | G | A | 0.04 | Neuronal |
|  |  |  |  |  |  |  |  |  |
| BMI | rs10968576 | *LINGO2* | No | NA | G | A | 0.025 | Neuronal |
| BMI | rs11030104 | *BDNF* | Yes | BMI-raising allele also associated with regular smoking (which itself has a causal effect on BMI in opposite direction) | A | G | 0.041 | NA |
| BMI | rs11057405 | *CLIP1* | No | NA | G | A | 0.031 | Neuronal |
| BMI | rs11126666 | *KCNK3* | No | NA | A | G | 0.021 | Neuronal |
| BMI | rs11165643 | *PTBP2* | No | NA | T | C | 0.022 | Neuronal |
| BMI | rs11191560 | *NT5C2* | No | NA | C | T | 0.031 | Non-neuronal |
| BMI | rs11583200 | *ELAVL4* | No | NA | C | T | 0.018 | Neuronal |
| BMI | rs1167827 | *HIP1* | No | NA | G | A | 0.02 | Neuronal |
| BMI | rs11688816 | *EHBP1* | No | NA | G | A | 0.017 | Non-neuronal |
| BMI | rs11727676 | *HHIP* | No | NA | T | C | 0.036 | Non-neuronal |
| BMI | rs11847697 | *PRKD1* | No | NA | T | C | 0.049 | Non-neuronal |
| BMI | rs12286929 | *CADM1* | No | NA | G | A | 0.022 | Neuronal |
| BMI | rs12401738 | *FUBP1* | No | NA | A | G | 0.021 | Neuronal |
| BMI | rs12429545 | *OLFM4* | No | NA | A | G | 0.033 | Non-neuronal |
| BMI | rs12446632 | *GPRC5B* | No | NA | G | A | 0.04 | Neuronal |
| BMI | rs12566985 | *FPGT-TNNI3K* | No | NA | G | A | 0.024 | Non-neuronal |
| BMI | rs12885454 | *PRKD1* | No | NA | C | A | 0.021 | Neuronal |
| BMI | rs12940622 | *RPTOR* | No | NA | G | A | 0.018 | Non-neuronal |
| BMI | rs13021737 | *TMEM18* | No | NA | G | A | 0.06 | Neuronal |
| BMI | rs13078960 | *CADM2* | No | NA | G | T | 0.03 | Neuronal |
| BMI | rs13107325 | *SLC39A8* | Yes | Missense Ala/Thr polymorphism located in exon 7 of SLC39A8, which encodes a zinc transporter that also transports cadmium and manganese. It is also associated with BP and HDL levels, and presumably these and the BMI effect are secondary to the metal ion transport variation. | T | C | 0.048 | NA |
| BMI | rs13191362 | *PARK2* | No | NA | A | G | 0.028 | Neuronal |
| BMI | rs1516725 | *ETV5* | No | NA | C | T | 0.045 | Neuronal |
| BMI | rs1528435 | *UBE2E3* | No | NA | T | C | 0.018 | Non-neuronal |
| BMI | rs1558902 | *FTO* | No | NA | A | T | 0.082 | Neuronal |
| BMI | rs16851483 | *RASA2* | No | NA | T | G | 0.048 | Non-neuronal |
| BMI | rs16951275 | *MAP2K5* | No | NA | T | C | 0.031 | Neuronal |
| BMI | rs17001654 | *SCARB2* | No | NA | G | C | 0.031 | Non-neuronal |
| BMI | rs17024393 | *GNAT2* | No | NA | C | T | 0.066 | Non-neuronal |
| BMI | rs17094222 | *HIF1AN* | No | NA | C | T | 0.025 | Neuronal |
| BMI | rs17405819 | *HNF4G* | No | NA | T | C | 0.022 | Neuronal |
| BMI | rs17724992 | *PGPEP1* | No | NA | A | G | 0.019 | Non-neuronal |
| BMI | rs1808579 | *C18orf8* | No | NA | C | T | 0.017 | Neuronal |
| BMI | rs1928295 | *TLR4* | No | NA | T | C | 0.019 | Non-neuronal |
| BMI | rs2033529 | *TDRG1* | No | NA | G | A | 0.019 | Neuronal |
| BMI | rs2033732 | *RALYL* | No | NA | C | T | 0.019 | Non-neuronal |
| BMI | rs205262 | *C6orf106* | No | NA | G | A | 0.022 | Non-neuronal |
| BMI | rs2075650 | *TOMM40* | No | NA | A | G | 0.026 | Neuronal |
| BMI | rs2112347 | *POC5* | No | NA | T | G | 0.026 | Non-neuronal |
| BMI | rs2121279 | *LRP1B* | No | NA | T | C | 0.025 | Non-neuronal |
| BMI | rs2176598 | *HSD17B12* | No | NA | T | C | 0.02 | Non-neuronal |
| BMI | rs2207139 | *TFAP2B* | No | NA | G | A | 0.045 | Non-neuronal |
| BMI | rs2245368 | *PMS2L11* | No | NA | C | T | 0.032 | Non-neuronal |
| BMI | rs2287019 | *QPCTL* | No | NA | C | T | 0.036 | Neuronal |
| BMI | rs2365389 | *FHIT* | No | NA | C | T | 0.02 | Non-neuronal |
| BMI | rs2650492 | *SBK1* | No | NA | A | G | 0.021 | Non-neuronal |
| BMI | rs2820292 | *NAV1* | No | NA | C | A | 0.02 | Neuronal |
| BMI | rs29941 | *KCTD15* | No | NA | G | A | 0.018 | Neuronal |
| BMI | rs3101336 | *NEGR1* | No | NA | C | T | 0.033 | Neuronal |
| BMI | rs3736485 | *DMXL2* | No | NA | A | G | 0.018 | Neuronal |
| BMI | rs3810291 | *ZC3H4* | No | NA | A | G | 0.028 | Neuronal |
| BMI | rs3817334 | *MTCH2* | No | NA | T | C | 0.026 | Non-neuronal |
| BMI | rs3849570 | *GBE1* | No | NA | A | C | 0.019 | Neuronal |
| BMI | rs3888190 | *SH2B1* | Yes | Associated with lots of other traits and is a big haplotype | A | C | 0.031 | NA |
| BMI | rs4256980 | *TRIM66* | No | NA | G | C | 0.021 | Neuronal |
| BMI | rs4740619 | *C9orf93* | No | NA | T | C | 0.018 | Neuronal |
| BMI | rs543874 | *SEC16B* | No | NA | G | A | 0.048 | Non-neuronal |
| BMI | rs6477694 | *EPB41L4B* | No | NA | C | T | 0.017 | Non-neuronal |
| BMI | rs6567160 | *MC4R* | No | NA | C | T | 0.056 | Neuronal |
| BMI | rs657452 | *AGBL4* | No | NA | A | G | 0.023 | Non-neuronal |
| BMI | rs6804842 | *RARB* | No | NA | G | A | 0.019 | Neuronal |
| BMI | rs7138803 | *BCDIN3D* | No | NA | A | G | 0.032 | Neuronal |
| BMI | rs7141420 | *NRXN3* | No | NA | T | C | 0.024 | Neuronal |
| BMI | rs7243357 | *GRP* | No | NA | T | G | 0.022 | Neuronal |
| BMI | rs758747 | *NLRC3* | No | NA | T | C | 0.023 | Non-neuronal |
| BMI | rs7599312 | *ERBB4* | No | NA | G | A | 0.022 | Neuronal |
| BMI | rs7899106 | *GRID1* | No | NA | G | A | 0.04 | Neuronal |
| BMI | rs9400239 | *FOXO3* | No | NA | C | T | 0.019 | Neuronal |
| BMI | rs9581854 | *MTIF3* | No | NA | T | C | 0.03 | Non-neuronal |
| BMI | rs9925964 | *KAT8* | No | NA | A | G | 0.019 | Neuronal |

**Supplementary Table S4:** Results of the sensitivity analysis using the model adjustment suggested by Keller et al. (2014). Data are presented as in main results and with adjustment for covariates by depression and covariates by genetic risk score interaction terms.

| **Sensitivity analysis from Table 2.** | |  | |  |  |  |
| --- | --- | --- | --- | --- | --- | --- |
|  |  |  | |  |  |  |
| **Depression definition** | **Model** |  | | **interaction beta** | **interaction se** | **interaction p** |
| Major depression (MD) | With Keller et al adjustment | Cases vs controls | | 0.0035 | 0.0012 | 4.13E-03 |
| Major depression (MD) | Without Keller et al adjustment | Cases vs controls | | 0.0047 | 0.0012 | 6.98E-05 |
|  |  |  | |  |  |  |
| Major depression (MD) | With Keller et al adjustment | Cases not on treatment vs controls | | 0.0028 | 0.0013 | 3.15E-02 |
| Major depression (MD) | Without Keller et al adjustment | Cases not on treatment vs controls | | 0.0040 | 0.0013 | 1.32E-03 |
|  |  |  | |  |  |  |
| Major depression (MD) | With Keller et al adjustment | Cases on treatment vs controls | | 0.0072 | 0.0027 | 7.55E-03 |
| Major depression (MD) | Without Keller et al adjustment | Cases on treatment vs controls | | 0.0085 | 0.0027 | 1.36E-03 |
|  |  |  | |  |  |  |
| Major depression (MD) | With Keller et al adjustment | Cases on treatment vs cases not on treatment | | 0.0040 | 0.0031 | 1.91E-01 |
| Major depression (MD) | Without Keller et al adjustment | Cases on treatment vs cases not on treatment | | 0.0046 | 0.0031 | 1.35E-01 |
|  |  |  | |  |  |  |
| Depression symptoms (DS) | With Keller et al adjustment | Cases vs controls | | 0.0026 | 0.0010 | 9.93E-03 |
| Depression symptoms (DS) | Without Keller et al adjustment | Cases vs controls | | 0.0032 | 0.0010 | 7.27E-04 |
|  |  |  | |  |  |  |
| Depression symptoms (DS) | With Keller et al adjustment | Cases not on treatment vs controls | | 0.0009 | 0.0011 | 3.97E-01 |
| Depression symptoms (DS) | Without Keller et al adjustment | Cases not on treatment vs controls | | 0.0015 | 0.0011 | 1.58E-01 |
|  |  |  | |  |  |  |
| Depression symptoms (DS) | With Keller et al adjustment | Cases on treatment vs controls | | 0.0078 | 0.0018 | 1.53E-05 |
| Depression symptoms (DS) | Without Keller et al adjustment | Cases on treatment vs controls | | 0.0086 | 0.0018 | 1.40E-06 |
|  |  |  | |  |  |  |
| Depression symptoms (DS) | With Keller et al adjustment | Cases on treatment vs cases not on treatment | | 0.0056 | 0.0023 | 1.29E-02 |
| Depression symptoms (DS) | Without Keller et al adjustment | Cases on treatment vs cases not on treatment | | 0.0073 | 0.0022 | 9.24E-04 |
|  |  |  | |  |  |  |
| Depression severity | With Keller et al adjustment | Below vs above median score | | 0.0004 | 0.0002 | 3.01E-02 |
| Depression severity | Without Keller et al adjustment | Below vs above median score | | 0.0006 | 0.0002 | 4.18E-04 |
|  |  |  | |  |  |  |
| **Sensitivity analysis from Table 3.** | |  | |  |  |  |
|  |  |  | |  |  |  |
| **Depression definition** | **Model** |  | Sex strata | **interaction beta** | **interaction se** | **interaction p** |
| Major depression (MD) | With Keller et al adjustment | Cases vs controls | Females only | 0.0029 | 0.0016 | 7.40E-02 |
| Major depression (MD) | Without Keller et al adjustment | Cases vs controls | Females only | 0.0040 | 0.0016 | 1.20E-02 |
|  |  |  |  |  |  |  |
| Major depression (MD) | With Keller et al adjustment | Cases not on treatment vs controls | Females only | 0.0018 | 0.0017 | 2.93E-01 |
| Major depression (MD) | Without Keller et al adjustment | Cases not on treatment vs controls | Females only | 0.0030 | 0.0017 | 7.62E-02 |
|  |  |  |  |  |  |  |
| Major depression (MD) | With Keller et al adjustment | Cases on treatment vs controls | Females only | 0.0084 | 0.0034 | 1.40E-02 |
| Major depression (MD) | Without Keller et al adjustment | Cases on treatment vs controls | Females only | 0.0097 | 0.0034 | 4.32E-03 |
|  |  |  |  |  |  |  |
| Major depression (MD) | With Keller et al adjustment | Cases on treatment vs cases not on treatment | Females only | 0.0060 | 0.0038 | 1.18E-01 |
| Major depression (MD) | Without Keller et al adjustment | Cases on treatment vs cases not on treatment | Females only | 0.0068 | 0.0038 | 7.34E-02 |
|  |  |  |  |  |  |  |
| Depression symptoms (DS) | With Keller et al adjustment | Cases vs controls | Females only | 0.0034 | 0.0014 | 1.67E-02 |
| Depression symptoms (DS) | Without Keller et al adjustment | Cases vs controls | Females only | 0.0038 | 0.0013 | 4.30E-03 |
|  |  |  |  |  |  |  |
| Depression symptoms (DS) | With Keller et al adjustment | Cases not on treatment vs controls | Females only | 0.0014 | 0.0016 | 3.72E-01 |
| Depression symptoms (DS) | Without Keller et al adjustment | Cases not on treatment vs controls | Females only | 0.0018 | 0.0015 | 2.25E-01 |
|  |  |  |  |  |  |  |
| Depression symptoms (DS) | With Keller et al adjustment | Cases on treatment vs controls | Females only | 0.0091 | 0.0024 | 1.49E-04 |
| Depression symptoms (DS) | Without Keller et al adjustment | Cases on treatment vs controls | Females only | 0.0099 | 0.0024 | 3.15E-05 |
|  |  |  |  |  |  |  |
| Depression symptoms (DS) | With Keller et al adjustment | Cases on treatment vs cases not on treatment | Females only | 0.0060 | 0.0029 | 3.99E-02 |
| Depression symptoms (DS) | Without Keller et al adjustment | Cases on treatment vs cases not on treatment | Females only | 0.0080 | 0.0029 | 5.41E-03 |
|  |  |  |  |  |  |  |
| Depression severity | With Keller et al adjustment | Below vs above median score | Females only | 0.0001 | 0.0002 | 6.16E-01 |
| Depression severity | Without Keller et al adjustment | Below vs above median score | Females only | 0.0003 | 0.0002 | 1.97E-01 |
|  |  |  |  |  |  |  |
| Major depression (MD) | With Keller et al adjustment | Cases vs controls | Males only | 0.0049 | 0.0018 | 7.26E-03 |
| Major depression (MD) | Without Keller et al adjustment | Cases vs controls | Males only | 0.0056 | 0.0018 | 1.90E-03 |
|  |  |  |  |  |  |  |
| Major depression (MD) | With Keller et al adjustment | Cases not on treatment vs controls | Males only | 0.0049 | 0.0019 | 1.05E-02 |
| Major depression (MD) | Without Keller et al adjustment | Cases not on treatment vs controls | Males only | 0.0056 | 0.0019 | 3.01E-03 |
|  |  |  |  |  |  |  |
| Major depression (MD) | With Keller et al adjustment | Cases on treatment vs controls | Males only | 0.0037 | 0.0046 | 4.20E-01 |
| Major depression (MD) | Without Keller et al adjustment | Cases on treatment vs controls | Males only | 0.0042 | 0.0045 | 3.54E-01 |
|  |  |  |  |  |  |  |
| Major depression (MD) | With Keller et al adjustment | Cases on treatment vs cases not on treatment | Males only | 0.0049 | 0.0019 | 1.05E-02 |
| Major depression (MD) | Without Keller et al adjustment | Cases on treatment vs cases not on treatment | Males only | -0.0018 | 0.0051 | 7.21E-01 |
|  |  |  |  |  |  |  |
| Depression symptoms (DS) | With Keller et al adjustment | Cases vs controls | Males only | 0.0013 | 0.0014 | 3.54E-01 |
| Depression symptoms (DS) | Without Keller et al adjustment | Cases vs controls | Males only | 0.0017 | 0.0014 | 2.26E-01 |
|  |  |  |  |  |  |  |
| Depression symptoms (DS) | With Keller et al adjustment | Cases not on treatment vs controls | Males only | 0.0003 | 0.0016 | 8.46E-01 |
| Depression symptoms (DS) | Without Keller et al adjustment | Cases not on treatment vs controls | Males only | 0.0004 | 0.0015 | 7.83E-01 |
|  |  |  |  |  |  |  |
| Depression symptoms (DS) | With Keller et al adjustment | Cases on treatment vs controls | Males only | 0.0048 | 0.0028 | 9.03E-02 |
| Depression symptoms (DS) | Without Keller et al adjustment | Cases on treatment vs controls | Males only | 0.0057 | 0.0028 | 4.28E-02 |
|  |  |  |  |  |  |  |
| Depression symptoms (DS) | With Keller et al adjustment | Cases on treatment vs cases not on treatment | Males only | 0.0048 | 0.0028 | 9.03E-02 |
| Depression symptoms (DS) | Without Keller et al adjustment | Cases on treatment vs cases not on treatment | Males only | 0.0050 | 0.0034 | 1.41E-01 |
|  |  |  |  |  |  |  |
| Depression severity | With Keller et al adjustment | Below vs above median score | Males only | 0.0008 | 0.0002 | 8.86E-04 |
| Depression severity | Without Keller et al adjustment | Below vs above median score | Males only | 0.0010 | 0.0002 | 6.30E-05 |
|  |  |  |  |  |  |  |
| **Sensitivity analysis from Table 4.** | |  | |  |  |  |
|  |  |  | |  |  |  |
| **Depression definition** | **Model** |  | | **interaction beta** | **interaction se** | **interaction p** |
| Major depression (MD) | With Keller et al adjustment | rs10182181 by MD cases vs controls | | 0.0320 | 0.0093 | 5.68E-04 |
| Major depression (MD) | Without Keller et al adjustment | rs10182181 by MD cases vs controls | | 0.0380 | 0.0091 | 2.85E-05 |
|  |  |  | |  |  |  |
| Major depression (MD) | With Keller et al adjustment | rs9925964 by MD cases vs controls | | 0.0207 | 0.0097 | 3.25E-02 |
| Major depression (MD) | Without Keller et al adjustment | rs9925964 by MD cases vs controls | | 0.0262 | 0.0095 | 5.70E-03 |
|  |  |  | |  |  |  |
| Major depression (MD) | With Keller et al adjustment | rs13021737 by MD cases vs controls | | 0.0264 | 0.0123 | 3.14E-02 |
| Major depression (MD) | Without Keller et al adjustment | rs13021737 by MD cases vs controls | | 0.0315 | 0.0120 | 8.93E-03 |
|  |  |  | |  |  |  |
| Major depression (MD) | With Keller et al adjustment | rs17724992 by MD cases vs controls | | 0.0245 | 0.0105 | 1.99E-02 |
| Major depression (MD) | Without Keller et al adjustment | rs17724992 by MD cases vs controls | | 0.0248 | 0.0103 | 1.60E-02 |
|  |  |  | |  |  |  |
| Major depression (MD) | With Keller et al adjustment | rs17094222 by MD cases vs controls | | 0.0271 | 0.0113 | 1.66E-02 |
| Major depression (MD) | Without Keller et al adjustment | rs17094222 by MD cases vs controls | | 0.0246 | 0.0111 | 2.62E-02 |
|  |  |  | |  |  |  |
| Major depression (MD) | With Keller et al adjustment | rs3810291 by MD cases vs controls | | 0.0200 | 0.0100 | 4.49E-02 |
| Major depression (MD) | Without Keller et al adjustment | rs3810291 by MD cases vs controls | | 0.0196 | 0.0097 | 4.46E-02 |
|  |  |  | |  |  |  |
| Major depression (MD) | With Keller et al adjustment | rs17024393 by MD cases vs controls | | 0.0362 | 0.0295 | 2.20E-01 |
| Major depression (MD) | Without Keller et al adjustment | rs17024393 by MD cases vs controls | | 0.0576 | 0.0288 | 4.53E-02 |
|  |  |  | |  |  |  |
| Major depression (MD) | With Keller et al adjustment | rs17405819 by MD cases vs controls | | 0.0205 | 0.0102 | 4.42E-02 |
| Major depression (MD) | Without Keller et al adjustment | rs17405819 by MD cases vs controls | | 0.0200 | 0.0100 | 4.56E-02 |
|  |  |  | |  |  |  |
| Depression symptoms (DS) | With Keller et al adjustment | rs6567160 by DS cases vs controls | | 0.0236 | 0.0091 | 9.13E-03 |
| Depression symptoms (DS) | Without Keller et al adjustment | rs6567160 by DS cases vs controls | | 0.0259 | 0.0086 | 2.72E-03 |
|  |  |  | |  |  |  |
| Depression symptoms (DS) | With Keller et al adjustment | rs2287019 by DS cases vs controls | | 0.0239 | 0.0099 | 1.65E-02 |
| Depression symptoms (DS) | Without Keller et al adjustment | rs2287019 by DS cases vs controls | | 0.0238 | 0.0095 | 1.21E-02 |
|  |  |  | |  |  |  |
| Depression symptoms (DS) | With Keller et al adjustment | rs1808579 by DS cases vs controls | | 0.0159 | 0.0076 | 3.78E-02 |
| Depression symptoms (DS) | Without Keller et al adjustment | rs1808579 by DS cases vs controls | | 0.0146 | 0.0073 | 4.53E-02 |
|  |  |  | |  |  |  |
| Depression symptoms (DS) | With Keller et al adjustment | rs10733682 by DS cases vs controls | | 0.0157 | 0.0078 | 4.38E-02 |
| Depression symptoms (DS) | Without Keller et al adjustment | rs10733682 by DS cases vs controls | | 0.0148 | 0.0074 | 4.65E-02 |
|  |  |  | |  |  |  |

**Supplementary Table S5.** Results of association and interaction analyses between individual BMI SNPs and BMI split by depression cases and controls for all 73 SNPs investigated.

| **SNP** | **Depression definition** | **Beta association with BMI** | **SE** | **P association** | **P interaction** |
| --- | --- | --- | --- | --- | --- |
| rs1000940 | Depression symptoms (DS) controls | 0.0181 | 0.0030 | 1.13E-09 | 3.86E-01 |
| rs1000940 | Depression symptoms (DS) cases | 0.0256 | 0.0081 | 1.50E-03 |  |
| rs10132280 | Depression symptoms (DS) controls | 0.0206 | 0.0030 | 4.79E-12 | 8.22E-01 |
| rs10132280 | Depression symptoms (DS) cases | 0.0219 | 0.0081 | 7.03E-03 |  |
| rs1016287 | Depression symptoms (DS) controls | 0.0224 | 0.0030 | 5.42E-14 | 3.73E-01 |
| rs1016287 | Depression symptoms (DS) cases | 0.0148 | 0.0080 | 6.43E-02 |  |
| rs10182181 | Depression symptoms (DS) controls | 0.0324 | 0.0027 | 1.02E-32 | 6.79E-01 |
| rs10182181 | Depression symptoms (DS) cases | 0.0355 | 0.0074 | 1.60E-06 |  |
| rs10733682 | Depression symptoms (DS) controls | 0.0122 | 0.0028 | 9.95E-06 | 4.65E-02 |
| rs10733682 | Depression symptoms (DS) cases | 0.0260 | 0.0075 | 5.62E-04 |  |
| rs10938397 | Depression symptoms (DS) controls | 0.0314 | 0.0028 | 4.26E-30 | 5.68E-01 |
| rs10938397 | Depression symptoms (DS) cases | 0.0281 | 0.0074 | 1.59E-04 |  |
| rs10968576 | Depression symptoms (DS) controls | 0.0284 | 0.0029 | 1.52E-22 | 5.86E-01 |
| rs10968576 | Depression symptoms (DS) cases | 0.0240 | 0.0079 | 2.25E-03 |  |
| rs11057405 | Depression symptoms (DS) controls | 0.0271 | 0.0044 | 8.85E-10 | 6.07E-01 |
| rs11057405 | Depression symptoms (DS) cases | 0.0329 | 0.0119 | 5.91E-03 |  |
| rs11126666 | Depression symptoms (DS) controls | 0.0032 | 0.0031 | 3.00E-01 | 9.75E-01 |
| rs11126666 | Depression symptoms (DS) cases | 0.0033 | 0.0084 | 6.94E-01 |  |
| rs11165643 | Depression symptoms (DS) controls | 0.0184 | 0.0028 | 2.61E-11 | 3.74E-01 |
| rs11165643 | Depression symptoms (DS) cases | 0.0252 | 0.0075 | 7.76E-04 |  |
| rs11191560 | Depression symptoms (DS) controls | 0.0216 | 0.0051 | 1.91E-05 | 4.99E-01 |
| rs11191560 | Depression symptoms (DS) cases | 0.0310 | 0.0139 | 2.61E-02 |  |
| rs11583200 | Depression symptoms (DS) controls | 0.0164 | 0.0028 | 4.85E-09 | 2.51E-01 |
| rs11583200 | Depression symptoms (DS) cases | 0.0082 | 0.0076 | 2.80E-01 |  |
| rs1167827 | Depression symptoms (DS) controls | 0.0195 | 0.0027 | 1.24E-12 | 1.06E-01 |
| rs1167827 | Depression symptoms (DS) cases | 0.0319 | 0.0075 | 1.90E-05 |  |
| rs11688816 | Depression symptoms (DS) controls | 0.0091 | 0.0027 | 8.35E-04 | 5.95E-01 |
| rs11688816 | Depression symptoms (DS) cases | 0.0133 | 0.0074 | 7.22E-02 |  |
| rs11727676 | Depression symptoms (DS) controls | 0.0100 | 0.0046 | 2.99E-02 | 2.92E-01 |
| rs11727676 | Depression symptoms (DS) cases | -0.0027 | 0.0125 | 8.30E-01 |  |
| rs11847697 | Depression symptoms (DS) controls | 0.0205 | 0.0067 | 2.18E-03 | 2.81E-01 |
| rs11847697 | Depression symptoms (DS) cases | 0.0378 | 0.0179 | 3.43E-02 |  |
| rs12286929 | Depression symptoms (DS) controls | 0.0146 | 0.0027 | 8.92E-08 | 5.44E-01 |
| rs12286929 | Depression symptoms (DS) cases | 0.0100 | 0.0074 | 1.79E-01 |  |
| rs12401738 | Depression symptoms (DS) controls | 0.0164 | 0.0028 | 5.22E-09 | 2.24E-01 |
| rs12401738 | Depression symptoms (DS) cases | 0.0075 | 0.0076 | 3.23E-01 |  |
| rs12429545 | Depression symptoms (DS) controls | 0.0229 | 0.0041 | 2.22E-08 | 3.55E-01 |
| rs12429545 | Depression symptoms (DS) cases | 0.0327 | 0.0109 | 2.83E-03 |  |
| rs12446632 | Depression symptoms (DS) controls | 0.0257 | 0.0039 | 3.69E-11 | 5.79E-01 |
| rs12446632 | Depression symptoms (DS) cases | 0.0321 | 0.0105 | 2.36E-03 |  |
| rs12566985 | Depression symptoms (DS) controls | 0.0155 | 0.0027 | 1.73E-08 | 5.98E-01 |
| rs12566985 | Depression symptoms (DS) cases | 0.0108 | 0.0074 | 1.46E-01 |  |
| rs12885454 | Depression symptoms (DS) controls | 0.0132 | 0.0028 | 3.51E-06 | 1.29E-01 |
| rs12885454 | Depression symptoms (DS) cases | 0.0248 | 0.0077 | 1.30E-03 |  |
| rs12940622 | Depression symptoms (DS) controls | 0.0190 | 0.0027 | 4.15E-12 | 7.63E-01 |
| rs12940622 | Depression symptoms (DS) cases | 0.0171 | 0.0074 | 2.10E-02 |  |
| rs13021737 | Depression symptoms (DS) controls | 0.0545 | 0.0036 | 1.22E-51 | 7.58E-01 |
| rs13021737 | Depression symptoms (DS) cases | 0.0510 | 0.0098 | 1.87E-07 |  |
| rs13078960 | Depression symptoms (DS) controls | 0.0192 | 0.0034 | 1.57E-08 | 8.56E-01 |
| rs13078960 | Depression symptoms (DS) cases | 0.0204 | 0.0093 | 2.81E-02 |  |
| rs13191362 | Depression symptoms (DS) controls | 0.0224 | 0.0041 | 6.25E-08 | 1.58E-01 |
| rs13191362 | Depression symptoms (DS) cases | 0.0074 | 0.0112 | 5.09E-01 |  |
| rs1516725 | Depression symptoms (DS) controls | 0.0268 | 0.0040 | 1.27E-11 | 1.30E-01 |
| rs1516725 | Depression symptoms (DS) cases | 0.0414 | 0.0108 | 1.30E-04 |  |
| rs1528435 | Depression symptoms (DS) controls | 0.0109 | 0.0028 | 1.05E-04 | 5.06E-01 |
| rs1528435 | Depression symptoms (DS) cases | 0.0152 | 0.0076 | 4.64E-02 |  |
| rs1558902 | Depression symptoms (DS) controls | 0.0711 | 0.0028 | 6.37E-145 | 5.20E-01 |
| rs1558902 | Depression symptoms (DS) cases | 0.0768 | 0.0075 | 1.41E-24 |  |
| rs16851483 | Depression symptoms (DS) controls | 0.0324 | 0.0055 | 3.65E-09 | 7.24E-01 |
| rs16851483 | Depression symptoms (DS) cases | 0.0374 | 0.0148 | 1.18E-02 |  |
| rs16951275 | Depression symptoms (DS) controls | 0.0289 | 0.0032 | 5.63E-19 | 3.25E-01 |
| rs16951275 | Depression symptoms (DS) cases | 0.0369 | 0.0088 | 2.63E-05 |  |
| rs17001654 | Depression symptoms (DS) controls | 0.0084 | 0.0039 | 2.96E-02 | 4.54E-01 |
| rs17001654 | Depression symptoms (DS) cases | 0.0163 | 0.0105 | 1.22E-01 |  |
| rs17024393 | Depression symptoms (DS) controls | 0.0595 | 0.0086 | 4.40E-12 | 4.01E-01 |
| rs17024393 | Depression symptoms (DS) cases | 0.0793 | 0.0231 | 5.88E-04 |  |
| rs17094222 | Depression symptoms (DS) controls | 0.0125 | 0.0033 | 1.79E-04 | 4.65E-01 |
| rs17094222 | Depression symptoms (DS) cases | 0.0192 | 0.0090 | 3.31E-02 |  |
| rs17405819 | Depression symptoms (DS) controls | 0.0234 | 0.0030 | 3.59E-15 | 8.09E-01 |
| rs17405819 | Depression symptoms (DS) cases | 0.0221 | 0.0081 | 6.25E-03 |  |
| rs17724992 | Depression symptoms (DS) controls | 0.0158 | 0.0031 | 2.97E-07 | 1.88E-01 |
| rs17724992 | Depression symptoms (DS) cases | 0.0259 | 0.0084 | 1.97E-03 |  |
| rs1808579 | Depression symptoms (DS) controls | 0.0176 | 0.0027 | 9.76E-11 | 4.53E-02 |
| rs1808579 | Depression symptoms (DS) cases | 0.0318 | 0.0073 | 1.54E-05 |  |
| rs1928295 | Depression symptoms (DS) controls | 0.0099 | 0.0027 | 2.90E-04 | 4.09E-01 |
| rs1928295 | Depression symptoms (DS) cases | 0.0158 | 0.0074 | 3.32E-02 |  |
| rs2033529 | Depression symptoms (DS) controls | 0.0195 | 0.0030 | 9.39E-11 | 8.33E-01 |
| rs2033529 | Depression symptoms (DS) cases | 0.0207 | 0.0082 | 1.14E-02 |  |
| rs2033732 | Depression symptoms (DS) controls | 0.0075 | 0.0031 | 1.66E-02 | 1.23E-01 |
| rs2033732 | Depression symptoms (DS) cases | 0.0216 | 0.0085 | 1.08E-02 |  |
| rs205262 | Depression symptoms (DS) controls | 0.0266 | 0.0031 | 5.03E-18 | 6.54E-01 |
| rs205262 | Depression symptoms (DS) cases | 0.0299 | 0.0083 | 3.27E-04 |  |
| rs2075650 | Depression symptoms (DS) controls | 0.0244 | 0.0039 | 2.42E-10 | 5.75E-01 |
| rs2075650 | Depression symptoms (DS) cases | 0.0194 | 0.0106 | 6.63E-02 |  |
| rs2112347 | Depression symptoms (DS) controls | 0.0294 | 0.0028 | 3.50E-25 | 4.31E-01 |
| rs2112347 | Depression symptoms (DS) cases | 0.0231 | 0.0078 | 2.89E-03 |  |
| rs2121279 | Depression symptoms (DS) controls | 0.0092 | 0.0041 | 2.48E-02 | 2.36E-01 |
| rs2121279 | Depression symptoms (DS) cases | 0.0216 | 0.0112 | 5.39E-02 |  |
| rs2176598 | Depression symptoms (DS) controls | 0.0192 | 0.0032 | 1.13E-09 | 7.59E-01 |
| rs2176598 | Depression symptoms (DS) cases | 0.0207 | 0.0086 | 1.54E-02 |  |
| rs2207139 | Depression symptoms (DS) controls | 0.0432 | 0.0036 | 9.14E-33 | 6.14E-01 |
| rs2207139 | Depression symptoms (DS) cases | 0.0472 | 0.0098 | 1.37E-06 |  |
| rs2245368 | Depression symptoms (DS) controls | 0.0244 | 0.0036 | 1.91E-11 | 7.72E-01 |
| rs2245368 | Depression symptoms (DS) cases | 0.0219 | 0.0099 | 2.60E-02 |  |
| rs2287019 | Depression symptoms (DS) controls | 0.0305 | 0.0035 | 6.74E-18 | 1.21E-02 |
| rs2287019 | Depression symptoms (DS) cases | 0.0548 | 0.0096 | 1.10E-08 |  |
| rs2365389 | Depression symptoms (DS) controls | 0.0145 | 0.0028 | 1.70E-07 | 5.89E-02 |
| rs2365389 | Depression symptoms (DS) cases | 0.0276 | 0.0075 | 2.42E-04 |  |
| rs2650492 | Depression symptoms (DS) controls | 0.0165 | 0.0030 | 3.33E-08 | 2.45E-01 |
| rs2650492 | Depression symptoms (DS) cases | 0.0260 | 0.0081 | 1.28E-03 |  |
| rs2820292 | Depression symptoms (DS) controls | 0.0197 | 0.0027 | 7.00E-13 | 2.25E-01 |
| rs2820292 | Depression symptoms (DS) cases | 0.0287 | 0.0074 | 1.19E-04 |  |
| rs29941 | Depression symptoms (DS) controls | 0.0119 | 0.0029 | 4.17E-05 | 2.07E-01 |
| rs29941 | Depression symptoms (DS) cases | 0.0213 | 0.0079 | 7.20E-03 |  |
| rs3101336 | Depression symptoms (DS) controls | 0.0244 | 0.0028 | 1.60E-18 | 5.93E-01 |
| rs3101336 | Depression symptoms (DS) cases | 0.0282 | 0.0075 | 1.80E-04 |  |
| rs3736485 | Depression symptoms (DS) controls | 0.0126 | 0.0027 | 4.16E-06 | 5.48E-01 |
| rs3736485 | Depression symptoms (DS) cases | 0.0081 | 0.0074 | 2.77E-01 |  |
| rs3810291 | Depression symptoms (DS) controls | 0.0275 | 0.0029 | 2.92E-21 | 7.62E-02 |
| rs3810291 | Depression symptoms (DS) cases | 0.0403 | 0.0079 | 3.72E-07 |  |
| rs3817334 | Depression symptoms (DS) controls | 0.0283 | 0.0028 | 1.36E-24 | 1.99E-01 |
| rs3817334 | Depression symptoms (DS) cases | 0.0185 | 0.0075 | 1.32E-02 |  |
| rs3849570 | Depression symptoms (DS) controls | 0.0107 | 0.0029 | 1.92E-04 | 2.94E-01 |
| rs3849570 | Depression symptoms (DS) cases | 0.0191 | 0.0077 | 1.36E-02 |  |
| rs4256980 | Depression symptoms (DS) controls | 0.0169 | 0.0029 | 3.82E-09 | 5.44E-01 |
| rs4256980 | Depression symptoms (DS) cases | 0.0128 | 0.0078 | 1.01E-01 |  |
| rs4740619 | Depression symptoms (DS) controls | 0.0174 | 0.0027 | 1.87E-10 | 1.14E-01 |
| rs4740619 | Depression symptoms (DS) cases | 0.0288 | 0.0074 | 1.03E-04 |  |
| rs543874 | Depression symptoms (DS) controls | 0.0446 | 0.0034 | 3.26E-40 | 1.03E-01 |
| rs543874 | Depression symptoms (DS) cases | 0.0593 | 0.0091 | 8.21E-11 |  |
| rs6477694 | Depression symptoms (DS) controls | 0.0132 | 0.0029 | 3.46E-06 | 5.68E-01 |
| rs6477694 | Depression symptoms (DS) cases | 0.0082 | 0.0078 | 2.88E-01 |  |
| rs6567160 | Depression symptoms (DS) controls | 0.0449 | 0.0032 | 2.04E-44 | 2.72E-03 |
| rs6567160 | Depression symptoms (DS) cases | 0.0707 | 0.0087 | 5.29E-16 |  |
| rs657452 | Depression symptoms (DS) controls | 0.0157 | 0.0028 | 1.86E-08 | 6.47E-01 |
| rs657452 | Depression symptoms (DS) cases | 0.0129 | 0.0076 | 8.81E-02 |  |
| rs6804842 | Depression symptoms (DS) controls | 0.0141 | 0.0028 | 2.87E-07 | 3.56E-01 |
| rs6804842 | Depression symptoms (DS) cases | 0.0068 | 0.0075 | 3.67E-01 |  |
| rs7138803 | Depression symptoms (DS) controls | 0.0263 | 0.0028 | 1.32E-20 | 4.77E-01 |
| rs7138803 | Depression symptoms (DS) cases | 0.0319 | 0.0076 | 2.82E-05 |  |
| rs7141420 | Depression symptoms (DS) controls | 0.0242 | 0.0027 | 1.08E-18 | 8.01E-01 |
| rs7141420 | Depression symptoms (DS) cases | 0.0227 | 0.0074 | 2.24E-03 |  |
| rs7243357 | Depression symptoms (DS) controls | 0.0209 | 0.0036 | 5.60E-09 | 1.51E-01 |
| rs7243357 | Depression symptoms (DS) cases | 0.0066 | 0.0097 | 4.95E-01 |  |
| rs758747 | Depression symptoms (DS) controls | 0.0125 | 0.0031 | 4.16E-05 | 6.12E-01 |
| rs758747 | Depression symptoms (DS) cases | 0.0092 | 0.0083 | 2.70E-01 |  |
| rs7599312 | Depression symptoms (DS) controls | 0.0175 | 0.0031 | 1.60E-08 | 7.23E-01 |
| rs7599312 | Depression symptoms (DS) cases | 0.0151 | 0.0084 | 7.43E-02 |  |
| rs7899106 | Depression symptoms (DS) controls | 0.0237 | 0.0063 | 1.57E-04 | 8.62E-01 |
| rs7899106 | Depression symptoms (DS) cases | 0.0211 | 0.0169 | 2.12E-01 |  |
| rs9400239 | Depression symptoms (DS) controls | 0.0158 | 0.0030 | 1.31E-07 | 6.45E-01 |
| rs9400239 | Depression symptoms (DS) cases | 0.0112 | 0.0081 | 1.65E-01 |  |
| rs9581854 | Depression symptoms (DS) controls | 0.0125 | 0.0035 | 3.98E-04 | 5.22E-01 |
| rs9581854 | Depression symptoms (DS) cases | 0.0191 | 0.0096 | 4.62E-02 |  |
| rs9925964 | Depression symptoms (DS) controls | 0.0229 | 0.0028 | 8.16E-16 | 8.26E-01 |
| rs9925964 | Depression symptoms (DS) cases | 0.0211 | 0.0077 | 6.10E-03 |  |
| rs1000940 | Major depression (MD) controls | 0.0171 | 0.0047 | 3.06E-04 | 7.22E-01 |
| rs1000940 | Major depression (MD) cases | 0.0210 | 0.0094 | 2.50E-02 |  |
| rs10132280 | Major depression (MD) controls | 0.0233 | 0.0047 | 8.96E-07 | 9.87E-01 |
| rs10132280 | Major depression (MD) cases | 0.0233 | 0.0094 | 1.35E-02 |  |
| rs1016287 | Major depression (MD) controls | 0.0216 | 0.0047 | 4.98E-06 | 4.09E-01 |
| rs1016287 | Major depression (MD) cases | 0.0132 | 0.0094 | 1.58E-01 |  |
| rs10182181 | Major depression (MD) controls | 0.0270 | 0.0043 | 4.88E-10 | 2.85E-05 |
| rs10182181 | Major depression (MD) cases | 0.0642 | 0.0085 | 5.64E-14 |  |
| rs10733682 | Major depression (MD) controls | 0.0091 | 0.0044 | 3.85E-02 | 8.51E-01 |
| rs10733682 | Major depression (MD) cases | 0.0081 | 0.0087 | 3.50E-01 |  |
| rs10938397 | Major depression (MD) controls | 0.0303 | 0.0044 | 5.06E-12 | 8.66E-01 |
| rs10938397 | Major depression (MD) cases | 0.0321 | 0.0086 | 2.00E-04 |  |
| rs10968576 | Major depression (MD) controls | 0.0225 | 0.0046 | 1.15E-06 | 1.62E-01 |
| rs10968576 | Major depression (MD) cases | 0.0351 | 0.0092 | 1.29E-04 |  |
| rs11057405 | Major depression (MD) controls | 0.0344 | 0.0071 | 1.11E-06 | 1.11E-01 |
| rs11057405 | Major depression (MD) cases | 0.0120 | 0.0141 | 3.94E-01 |  |
| rs11126666 | Major depression (MD) controls | -0.0002 | 0.0050 | 9.68E-01 | 5.66E-01 |
| rs11126666 | Major depression (MD) cases | 0.0056 | 0.0098 | 5.69E-01 |  |
| rs11165643 | Major depression (MD) controls | 0.0135 | 0.0044 | 2.20E-03 | 1.58E-01 |
| rs11165643 | Major depression (MD) cases | 0.0266 | 0.0087 | 2.23E-03 |  |
| rs11191560 | Major depression (MD) controls | 0.0361 | 0.0080 | 6.73E-06 | 9.41E-02 |
| rs11191560 | Major depression (MD) cases | 0.0074 | 0.0163 | 6.50E-01 |  |
| rs11583200 | Major depression (MD) controls | 0.0167 | 0.0045 | 1.71E-04 | 6.65E-01 |
| rs11583200 | Major depression (MD) cases | 0.0199 | 0.0088 | 2.44E-02 |  |
| rs1167827 | Major depression (MD) controls | 0.0181 | 0.0044 | 3.39E-05 | 1.87E-01 |
| rs1167827 | Major depression (MD) cases | 0.0301 | 0.0086 | 5.03E-04 |  |
| rs11688816 | Major depression (MD) controls | 0.0076 | 0.0044 | 8.01E-02 | 6.32E-01 |
| rs11688816 | Major depression (MD) cases | 0.0031 | 0.0086 | 7.21E-01 |  |
| rs11727676 | Major depression (MD) controls | 0.0057 | 0.0073 | 4.33E-01 | 9.55E-01 |
| rs11727676 | Major depression (MD) cases | 0.0059 | 0.0146 | 6.85E-01 |  |
| rs11847697 | Major depression (MD) controls | 0.0129 | 0.0106 | 2.27E-01 | 2.41E-01 |
| rs11847697 | Major depression (MD) cases | 0.0391 | 0.0210 | 6.30E-02 |  |
| rs12286929 | Major depression (MD) controls | 0.0179 | 0.0043 | 3.72E-05 | 9.99E-01 |
| rs12286929 | Major depression (MD) cases | 0.0175 | 0.0086 | 4.17E-02 |  |
| rs12401738 | Major depression (MD) controls | 0.0084 | 0.0045 | 6.06E-02 | 1.12E-01 |
| rs12401738 | Major depression (MD) cases | 0.0220 | 0.0088 | 1.29E-02 |  |
| rs12429545 | Major depression (MD) controls | 0.0207 | 0.0065 | 1.47E-03 | 2.89E-01 |
| rs12429545 | Major depression (MD) cases | 0.0353 | 0.0129 | 6.19E-03 |  |
| rs12446632 | Major depression (MD) controls | 0.0278 | 0.0062 | 7.10E-06 | 5.22E-01 |
| rs12446632 | Major depression (MD) cases | 0.0359 | 0.0121 | 3.08E-03 |  |
| rs12566985 | Major depression (MD) controls | 0.0113 | 0.0044 | 9.31E-03 | 8.58E-02 |
| rs12566985 | Major depression (MD) cases | 0.0266 | 0.0087 | 2.17E-03 |  |
| rs12885454 | Major depression (MD) controls | 0.0158 | 0.0045 | 4.95E-04 | 8.75E-01 |
| rs12885454 | Major depression (MD) cases | 0.0175 | 0.0090 | 5.10E-02 |  |
| rs12940622 | Major depression (MD) controls | 0.0156 | 0.0044 | 3.30E-04 | 7.27E-01 |
| rs12940622 | Major depression (MD) cases | 0.0183 | 0.0086 | 3.39E-02 |  |
| rs13021737 | Major depression (MD) controls | 0.0454 | 0.0057 | 2.45E-15 | 8.93E-03 |
| rs13021737 | Major depression (MD) cases | 0.0761 | 0.0113 | 1.72E-11 |  |
| rs13078960 | Major depression (MD) controls | 0.0112 | 0.0054 | 3.85E-02 | 1.46E-01 |
| rs13078960 | Major depression (MD) cases | 0.0279 | 0.0108 | 9.52E-03 |  |
| rs13191362 | Major depression (MD) controls | 0.0180 | 0.0066 | 6.18E-03 | 7.07E-01 |
| rs13191362 | Major depression (MD) cases | 0.0125 | 0.0131 | 3.40E-01 |  |
| rs1516725 | Major depression (MD) controls | 0.0220 | 0.0063 | 4.69E-04 | 1.08E-01 |
| rs1516725 | Major depression (MD) cases | 0.0441 | 0.0125 | 4.46E-04 |  |
| rs1528435 | Major depression (MD) controls | 0.0194 | 0.0045 | 1.38E-05 | 9.87E-01 |
| rs1528435 | Major depression (MD) cases | 0.0200 | 0.0088 | 2.30E-02 |  |
| rs1558902 | Major depression (MD) controls | 0.0691 | 0.0044 | 3.16E-55 | 5.52E-01 |
| rs1558902 | Major depression (MD) cases | 0.0745 | 0.0087 | 1.28E-17 |  |
| rs16851483 | Major depression (MD) controls | 0.0136 | 0.0088 | 1.22E-01 | 5.98E-02 |
| rs16851483 | Major depression (MD) cases | 0.0475 | 0.0172 | 5.81E-03 |  |
| rs16951275 | Major depression (MD) controls | 0.0333 | 0.0052 | 1.18E-10 | 1.73E-01 |
| rs16951275 | Major depression (MD) cases | 0.0179 | 0.0103 | 8.21E-02 |  |
| rs17001654 | Major depression (MD) controls | 0.0090 | 0.0062 | 1.45E-01 | 7.70E-01 |
| rs17001654 | Major depression (MD) cases | 0.0060 | 0.0121 | 6.18E-01 |  |
| rs17024393 | Major depression (MD) controls | 0.0562 | 0.0138 | 4.79E-05 | 4.53E-02 |
| rs17024393 | Major depression (MD) cases | 0.1127 | 0.0270 | 2.95E-05 |  |
| rs17094222 | Major depression (MD) controls | 0.0068 | 0.0053 | 1.97E-01 | 2.62E-02 |
| rs17094222 | Major depression (MD) cases | 0.0312 | 0.0104 | 2.72E-03 |  |
| rs17405819 | Major depression (MD) controls | 0.0133 | 0.0047 | 4.79E-03 | 4.56E-02 |
| rs17405819 | Major depression (MD) cases | 0.0338 | 0.0094 | 3.40E-04 |  |
| rs17724992 | Major depression (MD) controls | 0.0086 | 0.0049 | 7.90E-02 | 1.60E-02 |
| rs17724992 | Major depression (MD) cases | 0.0327 | 0.0097 | 7.30E-04 |  |
| rs1808579 | Major depression (MD) controls | 0.0178 | 0.0043 | 4.26E-05 | 4.80E-01 |
| rs1808579 | Major depression (MD) cases | 0.0111 | 0.0086 | 1.94E-01 |  |
| rs1928295 | Major depression (MD) controls | 0.0173 | 0.0044 | 7.33E-05 | 9.63E-01 |
| rs1928295 | Major depression (MD) cases | 0.0168 | 0.0087 | 5.32E-02 |  |
| rs2033529 | Major depression (MD) controls | 0.0130 | 0.0048 | 6.87E-03 | 1.13E-01 |
| rs2033529 | Major depression (MD) cases | 0.0290 | 0.0095 | 2.25E-03 |  |
| rs2033732 | Major depression (MD) controls | 0.0109 | 0.0050 | 2.81E-02 | 6.87E-01 |
| rs2033732 | Major depression (MD) cases | 0.0163 | 0.0098 | 9.57E-02 |  |
| rs205262 | Major depression (MD) controls | 0.0246 | 0.0049 | 4.86E-07 | 1.08E-01 |
| rs205262 | Major depression (MD) cases | 0.0080 | 0.0097 | 4.08E-01 |  |
| rs2075650 | Major depression (MD) controls | 0.0195 | 0.0062 | 1.72E-03 | 6.30E-01 |
| rs2075650 | Major depression (MD) cases | 0.0265 | 0.0123 | 3.13E-02 |  |
| rs2112347 | Major depression (MD) controls | 0.0318 | 0.0045 | 1.82E-12 | 1.20E-01 |
| rs2112347 | Major depression (MD) cases | 0.0156 | 0.0089 | 8.08E-02 |  |
| rs2121279 | Major depression (MD) controls | 0.0088 | 0.0065 | 1.76E-01 | 2.95E-01 |
| rs2121279 | Major depression (MD) cases | 0.0239 | 0.0131 | 6.77E-02 |  |
| rs2176598 | Major depression (MD) controls | 0.0164 | 0.0050 | 1.12E-03 | 5.53E-01 |
| rs2176598 | Major depression (MD) cases | 0.0214 | 0.0100 | 3.19E-02 |  |
| rs2207139 | Major depression (MD) controls | 0.0401 | 0.0058 | 4.62E-12 | 7.27E-01 |
| rs2207139 | Major depression (MD) cases | 0.0365 | 0.0113 | 1.31E-03 |  |
| rs2245368 | Major depression (MD) controls | 0.0298 | 0.0058 | 2.81E-07 | 2.82E-01 |
| rs2245368 | Major depression (MD) cases | 0.0169 | 0.0115 | 1.40E-01 |  |
| rs2287019 | Major depression (MD) controls | 0.0339 | 0.0056 | 1.86E-09 | 4.97E-01 |
| rs2287019 | Major depression (MD) cases | 0.0416 | 0.0112 | 2.09E-04 |  |
| rs2365389 | Major depression (MD) controls | 0.0176 | 0.0044 | 6.58E-05 | 7.89E-01 |
| rs2365389 | Major depression (MD) cases | 0.0149 | 0.0087 | 8.71E-02 |  |
| rs2650492 | Major depression (MD) controls | 0.0208 | 0.0048 | 1.24E-05 | 8.28E-01 |
| rs2650492 | Major depression (MD) cases | 0.0236 | 0.0094 | 1.20E-02 |  |
| rs2820292 | Major depression (MD) controls | 0.0161 | 0.0044 | 2.22E-04 | 4.39E-01 |
| rs2820292 | Major depression (MD) cases | 0.0222 | 0.0087 | 1.03E-02 |  |
| rs29941 | Major depression (MD) controls | 0.0098 | 0.0046 | 3.40E-02 | 7.17E-01 |
| rs29941 | Major depression (MD) cases | 0.0132 | 0.0091 | 1.50E-01 |  |
| rs3101336 | Major depression (MD) controls | 0.0223 | 0.0044 | 4.37E-07 | 5.90E-01 |
| rs3101336 | Major depression (MD) cases | 0.0178 | 0.0088 | 4.22E-02 |  |
| rs3736485 | Major depression (MD) controls | 0.0080 | 0.0044 | 6.83E-02 | 6.52E-01 |
| rs3736485 | Major depression (MD) cases | 0.0034 | 0.0086 | 6.93E-01 |  |
| rs3810291 | Major depression (MD) controls | 0.0175 | 0.0046 | 1.64E-04 | 4.46E-02 |
| rs3810291 | Major depression (MD) cases | 0.0367 | 0.0092 | 6.24E-05 |  |
| rs3817334 | Major depression (MD) controls | 0.0220 | 0.0044 | 6.44E-07 | 5.49E-01 |
| rs3817334 | Major depression (MD) cases | 0.0161 | 0.0087 | 6.45E-02 |  |
| rs3849570 | Major depression (MD) controls | 0.0184 | 0.0046 | 5.11E-05 | 4.94E-01 |
| rs3849570 | Major depression (MD) cases | 0.0120 | 0.0090 | 1.82E-01 |  |
| rs4256980 | Major depression (MD) controls | 0.0185 | 0.0046 | 4.88E-05 | 2.42E-01 |
| rs4256980 | Major depression (MD) cases | 0.0067 | 0.0090 | 4.60E-01 |  |
| rs4740619 | Major depression (MD) controls | 0.0250 | 0.0044 | 9.35E-09 | 7.25E-01 |
| rs4740619 | Major depression (MD) cases | 0.0276 | 0.0086 | 1.38E-03 |  |
| rs543874 | Major depression (MD) controls | 0.0443 | 0.0054 | 1.58E-16 | 1.22E-01 |
| rs543874 | Major depression (MD) cases | 0.0610 | 0.0106 | 7.80E-09 |  |
| rs6477694 | Major depression (MD) controls | 0.0090 | 0.0045 | 4.72E-02 | 4.73E-01 |
| rs6477694 | Major depression (MD) cases | 0.0022 | 0.0090 | 8.04E-01 |  |
| rs6567160 | Major depression (MD) controls | 0.0529 | 0.0051 | 6.03E-25 | 2.41E-01 |
| rs6567160 | Major depression (MD) cases | 0.0664 | 0.0101 | 4.96E-11 |  |
| rs657452 | Major depression (MD) controls | 0.0149 | 0.0044 | 7.92E-04 | 2.51E-01 |
| rs657452 | Major depression (MD) cases | 0.0253 | 0.0088 | 4.22E-03 |  |
| rs6804842 | Major depression (MD) controls | 0.0156 | 0.0044 | 3.84E-04 | 9.16E-01 |
| rs6804842 | Major depression (MD) cases | 0.0157 | 0.0087 | 7.20E-02 |  |
| rs7138803 | Major depression (MD) controls | 0.0220 | 0.0045 | 9.22E-07 | 8.77E-01 |
| rs7138803 | Major depression (MD) cases | 0.0234 | 0.0089 | 8.27E-03 |  |
| rs7141420 | Major depression (MD) controls | 0.0160 | 0.0044 | 2.49E-04 | 3.57E-01 |
| rs7141420 | Major depression (MD) cases | 0.0082 | 0.0086 | 3.41E-01 |  |
| rs7243357 | Major depression (MD) controls | 0.0168 | 0.0057 | 3.21E-03 | 8.38E-01 |
| rs7243357 | Major depression (MD) cases | 0.0192 | 0.0112 | 8.77E-02 |  |
| rs758747 | Major depression (MD) controls | 0.0048 | 0.0049 | 3.29E-01 | 3.85E-01 |
| rs758747 | Major depression (MD) cases | 0.0130 | 0.0098 | 1.82E-01 |  |
| rs7599312 | Major depression (MD) controls | 0.0129 | 0.0049 | 9.13E-03 | 9.47E-01 |
| rs7599312 | Major depression (MD) cases | 0.0132 | 0.0098 | 1.76E-01 |  |
| rs7899106 | Major depression (MD) controls | 0.0318 | 0.0099 | 1.37E-03 | 5.91E-01 |
| rs7899106 | Major depression (MD) cases | 0.0425 | 0.0199 | 3.28E-02 |  |
| rs9400239 | Major depression (MD) controls | 0.0063 | 0.0048 | 1.84E-01 | 7.11E-01 |
| rs9400239 | Major depression (MD) cases | 0.0094 | 0.0094 | 3.18E-01 |  |
| rs9581854 | Major depression (MD) controls | 0.0127 | 0.0056 | 2.29E-02 | 1.42E-01 |
| rs9581854 | Major depression (MD) cases | -0.0045 | 0.0111 | 6.85E-01 |  |
| rs9925964 | Major depression (MD) controls | 0.0129 | 0.0045 | 4.51E-03 | 5.70E-03 |
| rs9925964 | Major depression (MD) cases | 0.0381 | 0.0089 | 1.99E-05 |  |

**Supplementary Table S6.** Details of association between SNP and BMI in cases on treatment vs cases not on treatment for SNPs with a nominally significant SNP by depression treatment interaction.

| **Depression measure** | **Case/Control** | **SNP** | **Beta (SE)** | **P-value** | **P-value interaction** | **LOCUS** |
| --- | --- | --- | --- | --- | --- | --- |
| Major depression (MD) | Not on treatment | rs2207139 | 0.0172 (0.0122) | 1.60E-01 | 1.24E-03 | *RPS17P5* |
|  | On treatment | rs2207139 | 0.1157 (0.0299) | 1.08E-04 |  | *RPS17P5* |
| Major depression (MD) | Not on treatment | rs10968576 | 0.0262 (0.0098) | 7.36E-03 | 1.14E-02 | *LINGO2* |
|  | On treatment | rs10968576 | 0.0894 (0.0250) | 3.44E-04 |  | *LINGO2* |
| Major depression (MD) | Not on treatment | rs2287019 | 0.0528 (0.0120) | 1.07E-05 | 1.75E-02 | *QPCTL* |
|  | On treatment | rs2287019 | -0.0205 (0.0304) | 5.00E-01 |  | *QPCTL* |
| Major depression (MD) | Not on treatment | rs10182181 | 0.0560 (0.0092) | 9.54E-10 | 2.61E-02 | *ADCY3* |
|  | On treatment | rs10182181 | 0.1082 (0.0230) | 2.53E-06 |  | *ADCY3* |
| Major depression (MD) | Not on treatment | rs11057405 | -0.0024 (0.0151) | 8.74E-01 | 2.81E-02 | *CLIP1* |
|  | On treatment | rs11057405 | 0.0820 (0.0383) | 3.24E-02 |  | *CLIP1* |
| Major depression (MD) | Not on treatment | rs10733682 | 0.0004 (0.0093) | 9.69E-01 | 3.65E-02 | *LMX1B* |
|  | On treatment | rs10733682 | 0.0507 (0.0234) | 3.02E-02 |  | *LMX1B* |
| Major depression (MD) | Not on treatment | rs17405819 | 0.0249 (0.0101) | 1.36E-02 | 3.95E-02 | *HNF4G* |
|  | On treatment | rs17405819 | 0.0798 (0.0255) | 1.74E-03 |  | *HNF4G* |
|  |  |  |  |  |  |  |
| Depression symptoms (DS) | Not on treatment | rs17405819 | 0.0100 (0.0091) | 2.75E-01 | 9.50E-03 | *HNF4G* |
|  | On treatment | rs17405819 | 0.0588 (0.0169) | 5.02E-04 |  | *HNF4G* |
| Depression symptoms (DS) | Not on treatment | rs2033732 | 0.0115 (0.0095) | 2.29E-01 | 2.82E-02 | *RALYL* |
|  | On treatment | rs2033732 | 0.0547 (0.0176) | 1.88E-03 |  | *RALYL* |
| Depression symptoms (DS) | Not on treatment | rs543874 | 0.0478 (0.0103) | 3.40E-06 | 3.20E-02 | *SEC16B* |
|  | On treatment | rs543874 | 0.0926 (0.0191) | 1.28E-06 |  | *SEC16B* |
| Depression symptoms (DS) | Not on treatment | rs10968576 | 0.0149 (0.0089) | 9.39E-02 | 4.63E-02 | *LINGO2* |
|  | On treatment | rs10968576 | 0.0516 (0.0164) | 1.73E-03 |  | *LINGO2* |
| Depression symptoms (DS) | Not on treatment | rs12286929 | 0.0171 (0.0084) | 4.14E-02 | 4.90E-02 | *CADM1* |
|  | On treatment | rs12286929 | -0.0158 (0.0155) | 3.08E-01 |  | *CADM1* |
|  |  |  |  |  |  |  |

BMI= body mass index, SE= standard error, SNP= single nucleotide polymorphism

**Supplementary Table S7.** Results of association and interaction between SNP and BMI split by depression cases on treatment vs cases not on treatment (depression symptoms (DS) definition) for all 73 SNPs investigated.

| **SNP** | **Depression definition and antidepressant treatment status** | **Beta association** | **SE** | **P association** | **P interaction** |
| --- | --- | --- | --- | --- | --- |
| rs1000940 | Depression symptoms (DS) no treatment | 0.0317 | 0.0091 | 4.78E-04 | 2.51E-01 |
| rs1000940 | Depression symptoms (DS) on antidepressant treatment | 0.0095 | 0.0169 | 5.74E-01 |  |
| rs10132280 | Depression symptoms (DS) no treatment | 0.0164 | 0.0092 | 7.41E-02 | 1.97E-01 |
| rs10132280 | Depression symptoms (DS) on antidepressant treatment | 0.0383 | 0.0169 | 2.38E-02 |  |
| rs1016287 | Depression symptoms (DS) no treatment | 0.0184 | 0.0091 | 4.19E-02 | 4.26E-01 |
| rs1016287 | Depression symptoms (DS) on antidepressant treatment | 0.0031 | 0.0166 | 8.53E-01 |  |
| rs10182181 | Depression symptoms (DS) no treatment | 0.0380 | 0.0083 | 5.04E-06 | 6.58E-01 |
| rs10182181 | Depression symptoms (DS) on antidepressant treatment | 0.0301 | 0.0155 | 5.24E-02 |  |
| rs10733682 | Depression symptoms (DS) no treatment | 0.0309 | 0.0085 | 2.61E-04 | 2.08E-01 |
| rs10733682 | Depression symptoms (DS) on antidepressant treatment | 0.0093 | 0.0158 | 5.56E-01 |  |
| rs10938397 | Depression symptoms (DS) no treatment | 0.0241 | 0.0084 | 4.10E-03 | 4.70E-01 |
| rs10938397 | Depression symptoms (DS) on antidepressant treatment | 0.0403 | 0.0156 | 9.84E-03 |  |
| rs10968576 | Depression symptoms (DS) no treatment | 0.0149 | 0.0089 | 9.39E-02 | 4.63E-02 |
| rs10968576 | Depression symptoms (DS) on antidepressant treatment | 0.0516 | 0.0164 | 1.73E-03 |  |
| rs11057405 | Depression symptoms (DS) no treatment | 0.0201 | 0.0134 | 1.35E-01 | 1.05E-01 |
| rs11057405 | Depression symptoms (DS) on antidepressant treatment | 0.0638 | 0.0253 | 1.18E-02 |  |
| rs11126666 | Depression symptoms (DS) no treatment | 0.0096 | 0.0095 | 3.11E-01 | 1.85E-01 |
| rs11126666 | Depression symptoms (DS) on antidepressant treatment | -0.0151 | 0.0174 | 3.85E-01 |  |
| rs11165643 | Depression symptoms (DS) no treatment | 0.0294 | 0.0085 | 5.10E-04 | 2.12E-01 |
| rs11165643 | Depression symptoms (DS) on antidepressant treatment | 0.0093 | 0.0156 | 5.50E-01 |  |
| rs11191560 | Depression symptoms (DS) no treatment | 0.0366 | 0.0157 | 1.98E-02 | 5.34E-01 |
| rs11191560 | Depression symptoms (DS) on antidepressant treatment | 0.0181 | 0.0290 | 5.33E-01 |  |
| rs11583200 | Depression symptoms (DS) no treatment | 0.0091 | 0.0085 | 2.84E-01 | 9.75E-01 |
| rs11583200 | Depression symptoms (DS) on antidepressant treatment | 0.0071 | 0.0158 | 6.52E-01 |  |
| rs1167827 | Depression symptoms (DS) no treatment | 0.0254 | 0.0084 | 2.50E-03 | 2.51E-01 |
| rs1167827 | Depression symptoms (DS) on antidepressant treatment | 0.0443 | 0.0156 | 4.60E-03 |  |
| rs11688816 | Depression symptoms (DS) no treatment | 0.0133 | 0.0084 | 1.14E-01 | 8.53E-01 |
| rs11688816 | Depression symptoms (DS) on antidepressant treatment | 0.0166 | 0.0155 | 2.85E-01 |  |
| rs11727676 | Depression symptoms (DS) no treatment | -0.0027 | 0.0141 | 8.47E-01 | 9.53E-01 |
| rs11727676 | Depression symptoms (DS) on antidepressant treatment | 0.0001 | 0.0263 | 9.97E-01 |  |
| rs11847697 | Depression symptoms (DS) no treatment | 0.0219 | 0.0201 | 2.77E-01 | 7.56E-02 |
| rs11847697 | Depression symptoms (DS) on antidepressant treatment | 0.0925 | 0.0376 | 1.39E-02 |  |
| rs12286929 | Depression symptoms (DS) no treatment | 0.0171 | 0.0084 | 4.14E-02 | 4.90E-02 |
| rs12286929 | Depression symptoms (DS) on antidepressant treatment | -0.0158 | 0.0155 | 3.08E-01 |  |
| rs12401738 | Depression symptoms (DS) no treatment | 0.0131 | 0.0086 | 1.25E-01 | 3.31E-01 |
| rs12401738 | Depression symptoms (DS) on antidepressant treatment | -0.0033 | 0.0160 | 8.37E-01 |  |
| rs12429545 | Depression symptoms (DS) no treatment | 0.0406 | 0.0123 | 9.73E-04 | 3.18E-01 |
| rs12429545 | Depression symptoms (DS) on antidepressant treatment | 0.0133 | 0.0231 | 5.65E-01 |  |
| rs12446632 | Depression symptoms (DS) no treatment | 0.0253 | 0.0119 | 3.42E-02 | 2.01E-01 |
| rs12446632 | Depression symptoms (DS) on antidepressant treatment | 0.0556 | 0.0218 | 1.08E-02 |  |
| rs12566985 | Depression symptoms (DS) no treatment | 0.0165 | 0.0084 | 4.90E-02 | 2.56E-01 |
| rs12566985 | Depression symptoms (DS) on antidepressant treatment | -0.0043 | 0.0156 | 7.83E-01 |  |
| rs12885454 | Depression symptoms (DS) no treatment | 0.0229 | 0.0087 | 8.47E-03 | 4.40E-01 |
| rs12885454 | Depression symptoms (DS) on antidepressant treatment | 0.0360 | 0.0161 | 2.52E-02 |  |
| rs12940622 | Depression symptoms (DS) no treatment | 0.0209 | 0.0084 | 1.25E-02 | 3.88E-01 |
| rs12940622 | Depression symptoms (DS) on antidepressant treatment | 0.0069 | 0.0155 | 6.57E-01 |  |
| rs13021737 | Depression symptoms (DS) no treatment | 0.0411 | 0.0110 | 2.00E-04 | 9.32E-02 |
| rs13021737 | Depression symptoms (DS) on antidepressant treatment | 0.0799 | 0.0204 | 9.36E-05 |  |
| rs13078960 | Depression symptoms (DS) no treatment | 0.0136 | 0.0105 | 1.93E-01 | 1.42E-01 |
| rs13078960 | Depression symptoms (DS) on antidepressant treatment | 0.0441 | 0.0195 | 2.36E-02 |  |
| rs13191362 | Depression symptoms (DS) no treatment | 0.0142 | 0.0127 | 2.63E-01 | 2.83E-01 |
| rs13191362 | Depression symptoms (DS) on antidepressant treatment | -0.0103 | 0.0234 | 6.60E-01 |  |
| rs1516725 | Depression symptoms (DS) no treatment | 0.0296 | 0.0122 | 1.53E-02 | 5.34E-02 |
| rs1516725 | Depression symptoms (DS) on antidepressant treatment | 0.0809 | 0.0228 | 3.88E-04 |  |
| rs1528435 | Depression symptoms (DS) no treatment | 0.0180 | 0.0086 | 3.59E-02 | 5.28E-01 |
| rs1528435 | Depression symptoms (DS) on antidepressant treatment | 0.0082 | 0.0160 | 6.07E-01 |  |
| rs1558902 | Depression symptoms (DS) no treatment | 0.0712 | 0.0084 | 3.18E-17 | 2.01E-01 |
| rs1558902 | Depression symptoms (DS) on antidepressant treatment | 0.0940 | 0.0158 | 3.05E-09 |  |
| rs16851483 | Depression symptoms (DS) no treatment | 0.0428 | 0.0167 | 1.06E-02 | 5.78E-01 |
| rs16851483 | Depression symptoms (DS) on antidepressant treatment | 0.0205 | 0.0310 | 5.08E-01 |  |
| rs16951275 | Depression symptoms (DS) no treatment | 0.0370 | 0.0099 | 1.94E-04 | 5.59E-01 |
| rs16951275 | Depression symptoms (DS) on antidepressant treatment | 0.0496 | 0.0182 | 6.44E-03 |  |
| rs17001654 | Depression symptoms (DS) no treatment | 0.0130 | 0.0118 | 2.72E-01 | 5.22E-01 |
| rs17001654 | Depression symptoms (DS) on antidepressant treatment | 0.0266 | 0.0220 | 2.26E-01 |  |
| rs17024393 | Depression symptoms (DS) no treatment | 0.0848 | 0.0260 | 1.10E-03 | 7.22E-01 |
| rs17024393 | Depression symptoms (DS) on antidepressant treatment | 0.0648 | 0.0485 | 1.81E-01 |  |
| rs17094222 | Depression symptoms (DS) no treatment | 0.0252 | 0.0102 | 1.33E-02 | 1.86E-01 |
| rs17094222 | Depression symptoms (DS) on antidepressant treatment | -0.0018 | 0.0188 | 9.24E-01 |  |
| rs17405819 | Depression symptoms (DS) no treatment | 0.0100 | 0.0091 | 2.75E-01 | 9.50E-03 |
| rs17405819 | Depression symptoms (DS) on antidepressant treatment | 0.0588 | 0.0169 | 5.02E-04 |  |
| rs17724992 | Depression symptoms (DS) no treatment | 0.0224 | 0.0094 | 1.73E-02 | 4.03E-01 |
| rs17724992 | Depression symptoms (DS) on antidepressant treatment | 0.0386 | 0.0176 | 2.87E-02 |  |
| rs1808579 | Depression symptoms (DS) no treatment | 0.0265 | 0.0083 | 1.35E-03 | 1.39E-01 |
| rs1808579 | Depression symptoms (DS) on antidepressant treatment | 0.0513 | 0.0155 | 9.08E-04 |  |
| rs1928295 | Depression symptoms (DS) no treatment | 0.0154 | 0.0084 | 6.69E-02 | 8.98E-01 |
| rs1928295 | Depression symptoms (DS) on antidepressant treatment | 0.0173 | 0.0157 | 2.68E-01 |  |
| rs2033529 | Depression symptoms (DS) no treatment | 0.0176 | 0.0092 | 5.64E-02 | 6.47E-01 |
| rs2033529 | Depression symptoms (DS) on antidepressant treatment | 0.0255 | 0.0172 | 1.37E-01 |  |
| rs2033732 | Depression symptoms (DS) no treatment | 0.0115 | 0.0095 | 2.29E-01 | 2.82E-02 |
| rs2033732 | Depression symptoms (DS) on antidepressant treatment | 0.0547 | 0.0176 | 1.88E-03 |  |
| rs205262 | Depression symptoms (DS) no treatment | 0.0274 | 0.0094 | 3.57E-03 | 7.61E-01 |
| rs205262 | Depression symptoms (DS) on antidepressant treatment | 0.0329 | 0.0173 | 5.78E-02 |  |
| rs2075650 | Depression symptoms (DS) no treatment | 0.0132 | 0.0119 | 2.70E-01 | 3.05E-01 |
| rs2075650 | Depression symptoms (DS) on antidepressant treatment | 0.0376 | 0.0219 | 8.56E-02 |  |
| rs2112347 | Depression symptoms (DS) no treatment | 0.0243 | 0.0088 | 5.49E-03 | 8.22E-01 |
| rs2112347 | Depression symptoms (DS) on antidepressant treatment | 0.0202 | 0.0162 | 2.12E-01 |  |
| rs2121279 | Depression symptoms (DS) no treatment | 0.0226 | 0.0126 | 7.19E-02 | 8.41E-01 |
| rs2121279 | Depression symptoms (DS) on antidepressant treatment | 0.0149 | 0.0238 | 5.31E-01 |  |
| rs2176598 | Depression symptoms (DS) no treatment | 0.0216 | 0.0096 | 2.52E-02 | 9.23E-01 |
| rs2176598 | Depression symptoms (DS) on antidepressant treatment | 0.0197 | 0.0180 | 2.73E-01 |  |
| rs2207139 | Depression symptoms (DS) no treatment | 0.0401 | 0.0111 | 2.87E-04 | 3.64E-01 |
| rs2207139 | Depression symptoms (DS) on antidepressant treatment | 0.0589 | 0.0203 | 3.78E-03 |  |
| rs2245368 | Depression symptoms (DS) no treatment | 0.0151 | 0.0111 | 1.74E-01 | 2.75E-01 |
| rs2245368 | Depression symptoms (DS) on antidepressant treatment | 0.0402 | 0.0207 | 5.23E-02 |  |
| rs2287019 | Depression symptoms (DS) no treatment | 0.0620 | 0.0108 | 1.04E-08 | 1.97E-01 |
| rs2287019 | Depression symptoms (DS) on antidepressant treatment | 0.0360 | 0.0199 | 7.15E-02 |  |
| rs2365389 | Depression symptoms (DS) no treatment | 0.0222 | 0.0085 | 8.65E-03 | 1.60E-01 |
| rs2365389 | Depression symptoms (DS) on antidepressant treatment | 0.0472 | 0.0158 | 2.90E-03 |  |
| rs2650492 | Depression symptoms (DS) no treatment | 0.0280 | 0.0091 | 2.10E-03 | 7.22E-01 |
| rs2650492 | Depression symptoms (DS) on antidepressant treatment | 0.0229 | 0.0168 | 1.74E-01 |  |
| rs2820292 | Depression symptoms (DS) no treatment | 0.0295 | 0.0084 | 4.48E-04 | 9.26E-01 |
| rs2820292 | Depression symptoms (DS) on antidepressant treatment | 0.0263 | 0.0156 | 9.21E-02 |  |
| rs29941 | Depression symptoms (DS) no treatment | 0.0144 | 0.0089 | 1.06E-01 | 9.29E-02 |
| rs29941 | Depression symptoms (DS) on antidepressant treatment | 0.0439 | 0.0166 | 8.36E-03 |  |
| rs3101336 | Depression symptoms (DS) no treatment | 0.0291 | 0.0085 | 6.19E-04 | 8.53E-01 |
| rs3101336 | Depression symptoms (DS) on antidepressant treatment | 0.0249 | 0.0158 | 1.16E-01 |  |
| rs3736485 | Depression symptoms (DS) no treatment | 0.0104 | 0.0084 | 2.14E-01 | 7.02E-01 |
| rs3736485 | Depression symptoms (DS) on antidepressant treatment | 0.0040 | 0.0156 | 7.96E-01 |  |
| rs3810291 | Depression symptoms (DS) no treatment | 0.0348 | 0.0089 | 9.65E-05 | 2.37E-01 |
| rs3810291 | Depression symptoms (DS) on antidepressant treatment | 0.0565 | 0.0168 | 7.73E-04 |  |
| rs3817334 | Depression symptoms (DS) no treatment | 0.0218 | 0.0084 | 9.61E-03 | 4.30E-01 |
| rs3817334 | Depression symptoms (DS) on antidepressant treatment | 0.0087 | 0.0156 | 5.77E-01 |  |
| rs3849570 | Depression symptoms (DS) no treatment | 0.0219 | 0.0087 | 1.20E-02 | 5.48E-01 |
| rs3849570 | Depression symptoms (DS) on antidepressant treatment | 0.0128 | 0.0163 | 4.31E-01 |  |
| rs4256980 | Depression symptoms (DS) no treatment | 0.0121 | 0.0087 | 1.67E-01 | 9.64E-01 |
| rs4256980 | Depression symptoms (DS) on antidepressant treatment | 0.0129 | 0.0165 | 4.35E-01 |  |
| rs4740619 | Depression symptoms (DS) no treatment | 0.0312 | 0.0084 | 1.92E-04 | 6.84E-01 |
| rs4740619 | Depression symptoms (DS) on antidepressant treatment | 0.0251 | 0.0156 | 1.07E-01 |  |
| rs543874 | Depression symptoms (DS) no treatment | 0.0478 | 0.0103 | 3.40E-06 | 3.20E-02 |
| rs543874 | Depression symptoms (DS) on antidepressant treatment | 0.0926 | 0.0191 | 1.28E-06 |  |
| rs6477694 | Depression symptoms (DS) no treatment | 0.0102 | 0.0088 | 2.46E-01 | 5.10E-01 |
| rs6477694 | Depression symptoms (DS) on antidepressant treatment | -0.0018 | 0.0161 | 9.11E-01 |  |
| rs6567160 | Depression symptoms (DS) no treatment | 0.0632 | 0.0098 | 1.28E-10 | 1.28E-01 |
| rs6567160 | Depression symptoms (DS) on antidepressant treatment | 0.0932 | 0.0184 | 3.86E-07 |  |
| rs657452 | Depression symptoms (DS) no treatment | 0.0109 | 0.0085 | 2.04E-01 | 7.22E-01 |
| rs657452 | Depression symptoms (DS) on antidepressant treatment | 0.0172 | 0.0158 | 2.76E-01 |  |
| rs6804842 | Depression symptoms (DS) no treatment | 0.0076 | 0.0084 | 3.65E-01 | 9.72E-01 |
| rs6804842 | Depression symptoms (DS) on antidepressant treatment | 0.0079 | 0.0158 | 6.14E-01 |  |
| rs7138803 | Depression symptoms (DS) no treatment | 0.0251 | 0.0086 | 3.45E-03 | 1.14E-01 |
| rs7138803 | Depression symptoms (DS) on antidepressant treatment | 0.0532 | 0.0159 | 8.08E-04 |  |
| rs7141420 | Depression symptoms (DS) no treatment | 0.0260 | 0.0084 | 1.89E-03 | 3.24E-01 |
| rs7141420 | Depression symptoms (DS) on antidepressant treatment | 0.0103 | 0.0155 | 5.06E-01 |  |
| rs7243357 | Depression symptoms (DS) no treatment | 0.0034 | 0.0110 | 7.56E-01 | 5.88E-01 |
| rs7243357 | Depression symptoms (DS) on antidepressant treatment | 0.0187 | 0.0204 | 3.59E-01 |  |
| rs758747 | Depression symptoms (DS) no treatment | 0.0049 | 0.0094 | 6.03E-01 | 3.12E-01 |
| rs758747 | Depression symptoms (DS) on antidepressant treatment | 0.0241 | 0.0174 | 1.66E-01 |  |
| rs7599312 | Depression symptoms (DS) no treatment | 0.0122 | 0.0095 | 1.99E-01 | 7.04E-01 |
| rs7599312 | Depression symptoms (DS) on antidepressant treatment | 0.0212 | 0.0178 | 2.34E-01 |  |
| rs7899106 | Depression symptoms (DS) no treatment | 0.0172 | 0.0190 | 3.65E-01 | 5.34E-01 |
| rs7899106 | Depression symptoms (DS) on antidepressant treatment | 0.0393 | 0.0358 | 2.73E-01 |  |
| rs9400239 | Depression symptoms (DS) no treatment | 0.0078 | 0.0091 | 3.89E-01 | 4.94E-01 |
| rs9400239 | Depression symptoms (DS) on antidepressant treatment | 0.0193 | 0.0169 | 2.55E-01 |  |
| rs9581854 | Depression symptoms (DS) no treatment | 0.0162 | 0.0108 | 1.34E-01 | 5.18E-01 |
| rs9581854 | Depression symptoms (DS) on antidepressant treatment | 0.0291 | 0.0200 | 1.46E-01 |  |
| rs9925964 | Depression symptoms (DS) no treatment | 0.0211 | 0.0087 | 1.50E-02 | 9.41E-01 |
| rs9925964 | Depression symptoms (DS) on antidepressant treatment | 0.0217 | 0.0161 | 1.79E-01 |  |
| rs1000940 | Major depression (MD) no treatment | 0.0274 | 0.0100 | 6.23E-03 | 2.00E-01 |
| rs1000940 | Major depression (MD) on antidepressant treatment | -0.0069 | 0.0252 | 7.85E-01 |  |
| rs10132280 | Major depression (MD) no treatment | 0.0249 | 0.0101 | 1.38E-02 | 5.48E-01 |
| rs10132280 | Major depression (MD) on antidepressant treatment | 0.0052 | 0.0256 | 8.38E-01 |  |
| rs1016287 | Major depression (MD) no treatment | 0.0172 | 0.0101 | 8.73E-02 | 3.89E-01 |
| rs1016287 | Major depression (MD) on antidepressant treatment | -0.0054 | 0.0248 | 8.27E-01 |  |
| rs10182181 | Major depression (MD) no treatment | 0.0560 | 0.0092 | 9.54E-10 | 2.61E-02 |
| rs10182181 | Major depression (MD) on antidepressant treatment | 0.1082 | 0.0230 | 2.53E-06 |  |
| rs10733682 | Major depression (MD) no treatment | 0.0004 | 0.0093 | 9.69E-01 | 3.65E-02 |
| rs10733682 | Major depression (MD) on antidepressant treatment | 0.0507 | 0.0234 | 3.02E-02 |  |
| rs10938397 | Major depression (MD) no treatment | 0.0308 | 0.0092 | 8.78E-04 | 6.25E-01 |
| rs10938397 | Major depression (MD) on antidepressant treatment | 0.0426 | 0.0233 | 6.72E-02 |  |
| rs10968576 | Major depression (MD) no treatment | 0.0262 | 0.0098 | 7.36E-03 | 1.14E-02 |
| rs10968576 | Major depression (MD) on antidepressant treatment | 0.0894 | 0.0250 | 3.44E-04 |  |
| rs11057405 | Major depression (MD) no treatment | -0.0024 | 0.0151 | 8.74E-01 | 2.81E-02 |
| rs11057405 | Major depression (MD) on antidepressant treatment | 0.0820 | 0.0383 | 3.24E-02 |  |
| rs11126666 | Major depression (MD) no treatment | 0.0056 | 0.0105 | 5.96E-01 | 9.32E-01 |
| rs11126666 | Major depression (MD) on antidepressant treatment | 0.0038 | 0.0260 | 8.83E-01 |  |
| rs11165643 | Major depression (MD) no treatment | 0.0266 | 0.0093 | 4.20E-03 | 5.91E-01 |
| rs11165643 | Major depression (MD) on antidepressant treatment | 0.0122 | 0.0235 | 6.04E-01 |  |
| rs11191560 | Major depression (MD) no treatment | 0.0073 | 0.0174 | 6.77E-01 | 8.85E-01 |
| rs11191560 | Major depression (MD) on antidepressant treatment | 0.0156 | 0.0439 | 7.23E-01 |  |
| rs11583200 | Major depression (MD) no treatment | 0.0199 | 0.0095 | 3.58E-02 | 8.27E-01 |
| rs11583200 | Major depression (MD) on antidepressant treatment | 0.0265 | 0.0236 | 2.62E-01 |  |
| rs1167827 | Major depression (MD) no treatment | 0.0334 | 0.0093 | 3.08E-04 | 3.42E-01 |
| rs1167827 | Major depression (MD) on antidepressant treatment | 0.0104 | 0.0233 | 6.54E-01 |  |
| rs11688816 | Major depression (MD) no treatment | 0.0004 | 0.0093 | 9.69E-01 | 2.54E-01 |
| rs11688816 | Major depression (MD) on antidepressant treatment | 0.0278 | 0.0231 | 2.29E-01 |  |
| rs11727676 | Major depression (MD) no treatment | 0.0050 | 0.0158 | 7.52E-01 | 5.29E-01 |
| rs11727676 | Major depression (MD) on antidepressant treatment | 0.0280 | 0.0382 | 4.63E-01 |  |
| rs11847697 | Major depression (MD) no treatment | 0.0351 | 0.0224 | 1.17E-01 | 4.92E-01 |
| rs11847697 | Major depression (MD) on antidepressant treatment | 0.0761 | 0.0581 | 1.90E-01 |  |
| rs12286929 | Major depression (MD) no treatment | 0.0152 | 0.0092 | 9.86E-02 | 5.68E-01 |
| rs12286929 | Major depression (MD) on antidepressant treatment | 0.0280 | 0.0229 | 2.22E-01 |  |
| rs12401738 | Major depression (MD) no treatment | 0.0197 | 0.0095 | 3.76E-02 | 4.27E-01 |
| rs12401738 | Major depression (MD) on antidepressant treatment | 0.0395 | 0.0238 | 9.77E-02 |  |
| rs12429545 | Major depression (MD) no treatment | 0.0273 | 0.0138 | 4.80E-02 | 1.47E-01 |
| rs12429545 | Major depression (MD) on antidepressant treatment | 0.0839 | 0.0347 | 1.55E-02 |  |
| rs12446632 | Major depression (MD) no treatment | 0.0266 | 0.0130 | 4.12E-02 | 7.80E-02 |
| rs12446632 | Major depression (MD) on antidepressant treatment | 0.0823 | 0.0327 | 1.19E-02 |  |
| rs12566985 | Major depression (MD) no treatment | 0.0336 | 0.0093 | 3.05E-04 | 1.21E-01 |
| rs12566985 | Major depression (MD) on antidepressant treatment | -0.0009 | 0.0233 | 9.68E-01 |  |
| rs12885454 | Major depression (MD) no treatment | 0.0172 | 0.0096 | 7.38E-02 | 7.31E-01 |
| rs12885454 | Major depression (MD) on antidepressant treatment | 0.0244 | 0.0240 | 3.10E-01 |  |
| rs12940622 | Major depression (MD) no treatment | 0.0153 | 0.0092 | 9.82E-02 | 2.95E-01 |
| rs12940622 | Major depression (MD) on antidepressant treatment | 0.0356 | 0.0232 | 1.24E-01 |  |
| rs13021737 | Major depression (MD) no treatment | 0.0815 | 0.0121 | 1.99E-11 | 2.53E-01 |
| rs13021737 | Major depression (MD) on antidepressant treatment | 0.0515 | 0.0301 | 8.72E-02 |  |
| rs13078960 | Major depression (MD) no treatment | 0.0282 | 0.0116 | 1.49E-02 | 9.04E-01 |
| rs13078960 | Major depression (MD) on antidepressant treatment | 0.0236 | 0.0284 | 4.07E-01 |  |
| rs13191362 | Major depression (MD) no treatment | 0.0073 | 0.0141 | 6.03E-01 | 4.35E-01 |
| rs13191362 | Major depression (MD) on antidepressant treatment | 0.0331 | 0.0353 | 3.49E-01 |  |
| rs1516725 | Major depression (MD) no treatment | 0.0424 | 0.0134 | 1.56E-03 | 9.56E-01 |
| rs1516725 | Major depression (MD) on antidepressant treatment | 0.0412 | 0.0343 | 2.30E-01 |  |
| rs1528435 | Major depression (MD) no treatment | 0.0203 | 0.0095 | 3.18E-02 | 7.11E-01 |
| rs1528435 | Major depression (MD) on antidepressant treatment | 0.0314 | 0.0235 | 1.81E-01 |  |
| rs1558902 | Major depression (MD) no treatment | 0.0740 | 0.0093 | 1.96E-15 | 1.00E+00 |
| rs1558902 | Major depression (MD) on antidepressant treatment | 0.0744 | 0.0238 | 1.78E-03 |  |
| rs16851483 | Major depression (MD) no treatment | 0.0417 | 0.0184 | 2.35E-02 | 3.02E-01 |
| rs16851483 | Major depression (MD) on antidepressant treatment | 0.0847 | 0.0469 | 7.14E-02 |  |
| rs16951275 | Major depression (MD) no treatment | 0.0257 | 0.0111 | 2.00E-02 | 1.34E-01 |
| rs16951275 | Major depression (MD) on antidepressant treatment | -0.0152 | 0.0274 | 5.80E-01 |  |
| rs17001654 | Major depression (MD) no treatment | 0.0021 | 0.0129 | 8.71E-01 | 4.87E-01 |
| rs17001654 | Major depression (MD) on antidepressant treatment | 0.0263 | 0.0325 | 4.19E-01 |  |
| rs17024393 | Major depression (MD) no treatment | 0.1074 | 0.0290 | 2.17E-04 | 7.32E-01 |
| rs17024393 | Major depression (MD) on antidepressant treatment | 0.1349 | 0.0708 | 5.68E-02 |  |
| rs17094222 | Major depression (MD) no treatment | 0.0295 | 0.0112 | 8.11E-03 | 6.49E-01 |
| rs17094222 | Major depression (MD) on antidepressant treatment | 0.0407 | 0.0279 | 1.44E-01 |  |
| rs17405819 | Major depression (MD) no treatment | 0.0249 | 0.0101 | 1.36E-02 | 3.95E-02 |
| rs17405819 | Major depression (MD) on antidepressant treatment | 0.0798 | 0.0255 | 1.74E-03 |  |
| rs17724992 | Major depression (MD) no treatment | 0.0293 | 0.0104 | 4.71E-03 | 3.09E-01 |
| rs17724992 | Major depression (MD) on antidepressant treatment | 0.0544 | 0.0263 | 3.84E-02 |  |
| rs1808579 | Major depression (MD) no treatment | 0.0073 | 0.0092 | 4.23E-01 | 2.79E-01 |
| rs1808579 | Major depression (MD) on antidepressant treatment | 0.0347 | 0.0231 | 1.32E-01 |  |
| rs1928295 | Major depression (MD) no treatment | 0.0086 | 0.0093 | 3.53E-01 | 5.78E-02 |
| rs1928295 | Major depression (MD) on antidepressant treatment | 0.0535 | 0.0235 | 2.28E-02 |  |
| rs2033529 | Major depression (MD) no treatment | 0.0338 | 0.0102 | 8.82E-04 | 2.02E-01 |
| rs2033529 | Major depression (MD) on antidepressant treatment | 0.0012 | 0.0255 | 9.62E-01 |  |
| rs2033732 | Major depression (MD) no treatment | 0.0107 | 0.0105 | 3.12E-01 | 1.16E-01 |
| rs2033732 | Major depression (MD) on antidepressant treatment | 0.0555 | 0.0259 | 3.22E-02 |  |
| rs205262 | Major depression (MD) no treatment | 0.0126 | 0.0104 | 2.23E-01 | 3.09E-01 |
| rs205262 | Major depression (MD) on antidepressant treatment | -0.0183 | 0.0259 | 4.80E-01 |  |
| rs2075650 | Major depression (MD) no treatment | 0.0344 | 0.0131 | 8.85E-03 | 7.21E-02 |
| rs2075650 | Major depression (MD) on antidepressant treatment | -0.0216 | 0.0334 | 5.18E-01 |  |
| rs2112347 | Major depression (MD) no treatment | 0.0227 | 0.0096 | 1.77E-02 | 7.89E-02 |
| rs2112347 | Major depression (MD) on antidepressant treatment | -0.0205 | 0.0243 | 3.98E-01 |  |
| rs2121279 | Major depression (MD) no treatment | 0.0242 | 0.0140 | 8.38E-02 | 8.82E-01 |
| rs2121279 | Major depression (MD) on antidepressant treatment | 0.0322 | 0.0359 | 3.70E-01 |  |
| rs2176598 | Major depression (MD) no treatment | 0.0204 | 0.0107 | 5.59E-02 | 8.87E-01 |
| rs2176598 | Major depression (MD) on antidepressant treatment | 0.0290 | 0.0268 | 2.79E-01 |  |
| rs2207139 | Major depression (MD) no treatment | 0.0172 | 0.0122 | 1.60E-01 | 1.24E-03 |
| rs2207139 | Major depression (MD) on antidepressant treatment | 0.1157 | 0.0299 | 1.08E-04 |  |
| rs2245368 | Major depression (MD) no treatment | 0.0166 | 0.0123 | 1.77E-01 | 9.83E-01 |
| rs2245368 | Major depression (MD) on antidepressant treatment | 0.0205 | 0.0305 | 5.02E-01 |  |
| rs2287019 | Major depression (MD) no treatment | 0.0528 | 0.0120 | 1.07E-05 | 1.75E-02 |
| rs2287019 | Major depression (MD) on antidepressant treatment | -0.0205 | 0.0304 | 5.00E-01 |  |
| rs2365389 | Major depression (MD) no treatment | 0.0150 | 0.0093 | 1.08E-01 | 9.48E-01 |
| rs2365389 | Major depression (MD) on antidepressant treatment | 0.0156 | 0.0236 | 5.08E-01 |  |
| rs2650492 | Major depression (MD) no treatment | 0.0254 | 0.0101 | 1.17E-02 | 5.41E-01 |
| rs2650492 | Major depression (MD) on antidepressant treatment | 0.0156 | 0.0253 | 5.37E-01 |  |
| rs2820292 | Major depression (MD) no treatment | 0.0215 | 0.0093 | 2.02E-02 | 7.93E-01 |
| rs2820292 | Major depression (MD) on antidepressant treatment | 0.0267 | 0.0234 | 2.54E-01 |  |
| rs29941 | Major depression (MD) no treatment | 0.0079 | 0.0098 | 4.18E-01 | 1.43E-01 |
| rs29941 | Major depression (MD) on antidepressant treatment | 0.0415 | 0.0246 | 9.12E-02 |  |
| rs3101336 | Major depression (MD) no treatment | 0.0240 | 0.0094 | 1.08E-02 | 1.39E-01 |
| rs3101336 | Major depression (MD) on antidepressant treatment | -0.0124 | 0.0234 | 5.95E-01 |  |
| rs3736485 | Major depression (MD) no treatment | -0.0039 | 0.0092 | 6.70E-01 | 8.35E-02 |
| rs3736485 | Major depression (MD) on antidepressant treatment | 0.0369 | 0.0233 | 1.14E-01 |  |
| rs3810291 | Major depression (MD) no treatment | 0.0329 | 0.0098 | 8.05E-04 | 3.21E-01 |
| rs3810291 | Major depression (MD) on antidepressant treatment | 0.0547 | 0.0247 | 2.69E-02 |  |
| rs3817334 | Major depression (MD) no treatment | 0.0223 | 0.0094 | 1.75E-02 | 8.43E-02 |
| rs3817334 | Major depression (MD) on antidepressant treatment | -0.0184 | 0.0231 | 4.26E-01 |  |
| rs3849570 | Major depression (MD) no treatment | 0.0129 | 0.0096 | 1.81E-01 | 8.65E-01 |
| rs3849570 | Major depression (MD) on antidepressant treatment | 0.0103 | 0.0244 | 6.72E-01 |  |
| rs4256980 | Major depression (MD) no treatment | 0.0118 | 0.0096 | 2.23E-01 | 1.62E-01 |
| rs4256980 | Major depression (MD) on antidepressant treatment | -0.0272 | 0.0245 | 2.66E-01 |  |
| rs4740619 | Major depression (MD) no treatment | 0.0328 | 0.0093 | 3.91E-04 | 8.02E-02 |
| rs4740619 | Major depression (MD) on antidepressant treatment | -0.0087 | 0.0233 | 7.08E-01 |  |
| rs543874 | Major depression (MD) no treatment | 0.0582 | 0.0113 | 2.77E-07 | 4.34E-01 |
| rs543874 | Major depression (MD) on antidepressant treatment | 0.0761 | 0.0283 | 7.16E-03 |  |
| rs6477694 | Major depression (MD) no treatment | -0.0029 | 0.0096 | 7.62E-01 | 1.64E-01 |
| rs6477694 | Major depression (MD) on antidepressant treatment | 0.0316 | 0.0244 | 1.96E-01 |  |
| rs6567160 | Major depression (MD) no treatment | 0.0659 | 0.0108 | 1.13E-09 | 9.27E-01 |
| rs6567160 | Major depression (MD) on antidepressant treatment | 0.0664 | 0.0273 | 1.52E-02 |  |
| rs657452 | Major depression (MD) no treatment | 0.0265 | 0.0095 | 5.22E-03 | 7.60E-01 |
| rs657452 | Major depression (MD) on antidepressant treatment | 0.0187 | 0.0235 | 4.25E-01 |  |
| rs6804842 | Major depression (MD) no treatment | 0.0130 | 0.0093 | 1.65E-01 | 2.80E-01 |
| rs6804842 | Major depression (MD) on antidepressant treatment | 0.0384 | 0.0232 | 9.78E-02 |  |
| rs7138803 | Major depression (MD) no treatment | 0.0167 | 0.0095 | 7.92E-02 | 5.85E-02 |
| rs7138803 | Major depression (MD) on antidepressant treatment | 0.0594 | 0.0238 | 1.26E-02 |  |
| rs7141420 | Major depression (MD) no treatment | 0.0086 | 0.0093 | 3.51E-01 | 7.95E-01 |
| rs7141420 | Major depression (MD) on antidepressant treatment | 0.0034 | 0.0230 | 8.82E-01 |  |
| rs7243357 | Major depression (MD) no treatment | 0.0212 | 0.0120 | 7.85E-02 | 9.56E-01 |
| rs7243357 | Major depression (MD) on antidepressant treatment | 0.0165 | 0.0300 | 5.82E-01 |  |
| rs758747 | Major depression (MD) no treatment | 0.0142 | 0.0105 | 1.75E-01 | 6.87E-01 |
| rs758747 | Major depression (MD) on antidepressant treatment | 0.0023 | 0.0261 | 9.30E-01 |  |
| rs7599312 | Major depression (MD) no treatment | 0.0191 | 0.0104 | 6.68E-02 | 1.05E-01 |
| rs7599312 | Major depression (MD) on antidepressant treatment | -0.0256 | 0.0269 | 3.41E-01 |  |
| rs7899106 | Major depression (MD) no treatment | 0.0445 | 0.0215 | 3.80E-02 | 7.16E-01 |
| rs7899106 | Major depression (MD) on antidepressant treatment | 0.0245 | 0.0522 | 6.38E-01 |  |
| rs9400239 | Major depression (MD) no treatment | 0.0088 | 0.0101 | 3.81E-01 | 9.89E-01 |
| rs9400239 | Major depression (MD) on antidepressant treatment | 0.0042 | 0.0254 | 8.69E-01 |  |
| rs9581854 | Major depression (MD) no treatment | -0.0042 | 0.0119 | 7.25E-01 | 9.82E-01 |
| rs9581854 | Major depression (MD) on antidepressant treatment | -0.0037 | 0.0295 | 8.99E-01 |  |
| rs9925964 | Major depression (MD) no treatment | 0.0442 | 0.0096 | 4.06E-06 | 1.52E-01 |
| rs9925964 | Major depression (MD) on antidepressant treatment | 0.0100 | 0.0237 | 6.73E-01 |  |

**Supplementary figure S1**

**
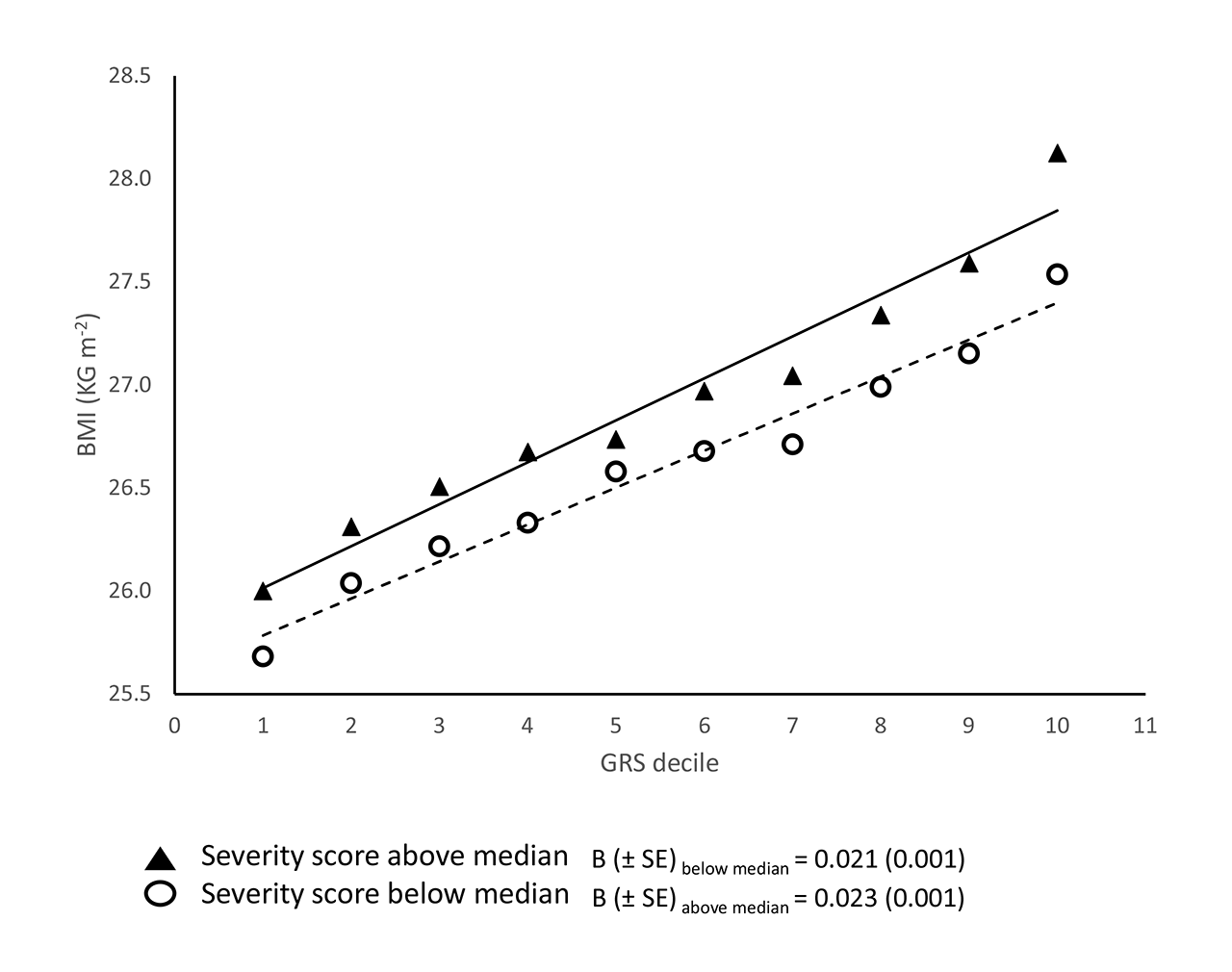
**

**Supplementary figure S2**

**
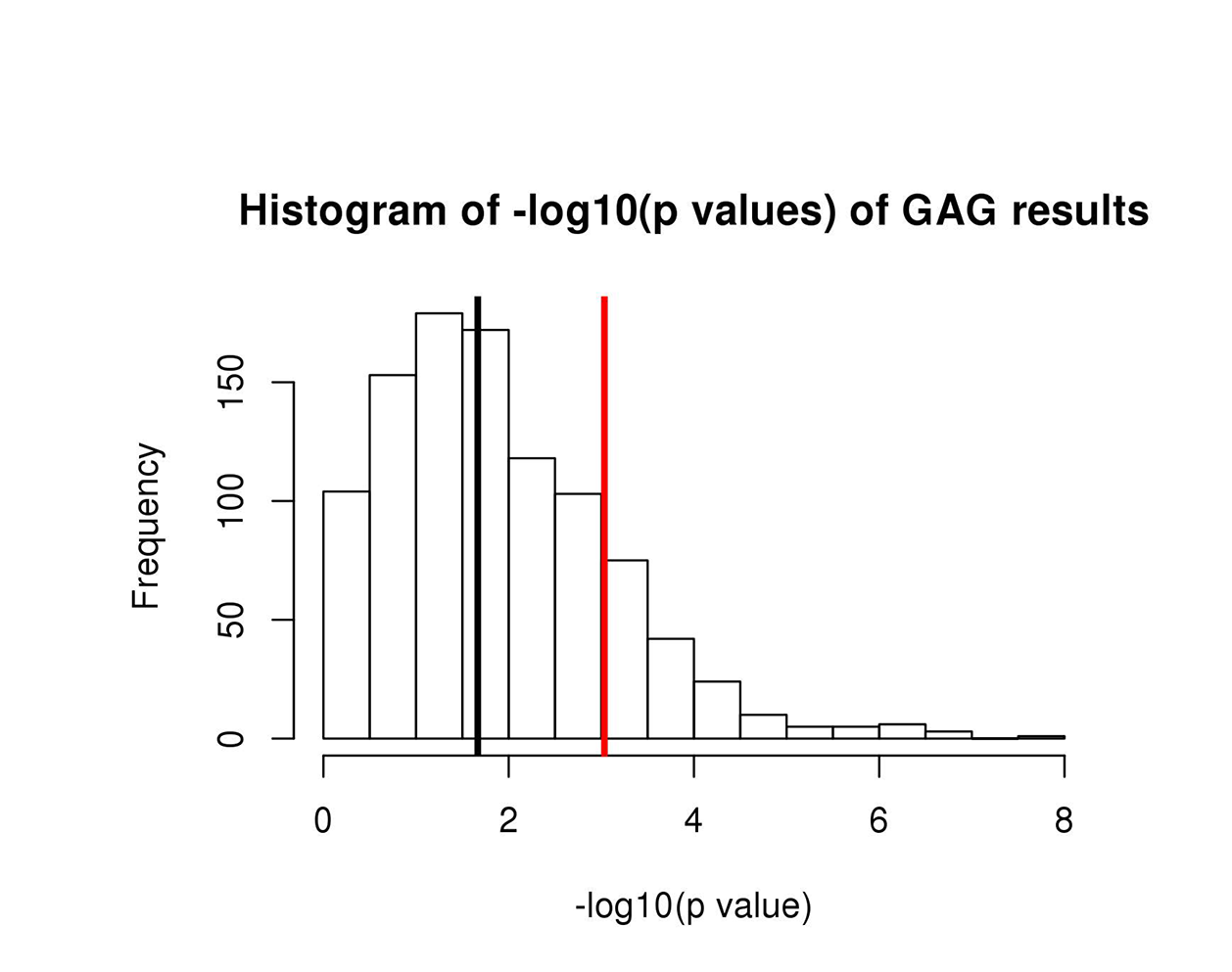
**

**Supplementary figure S3**

**
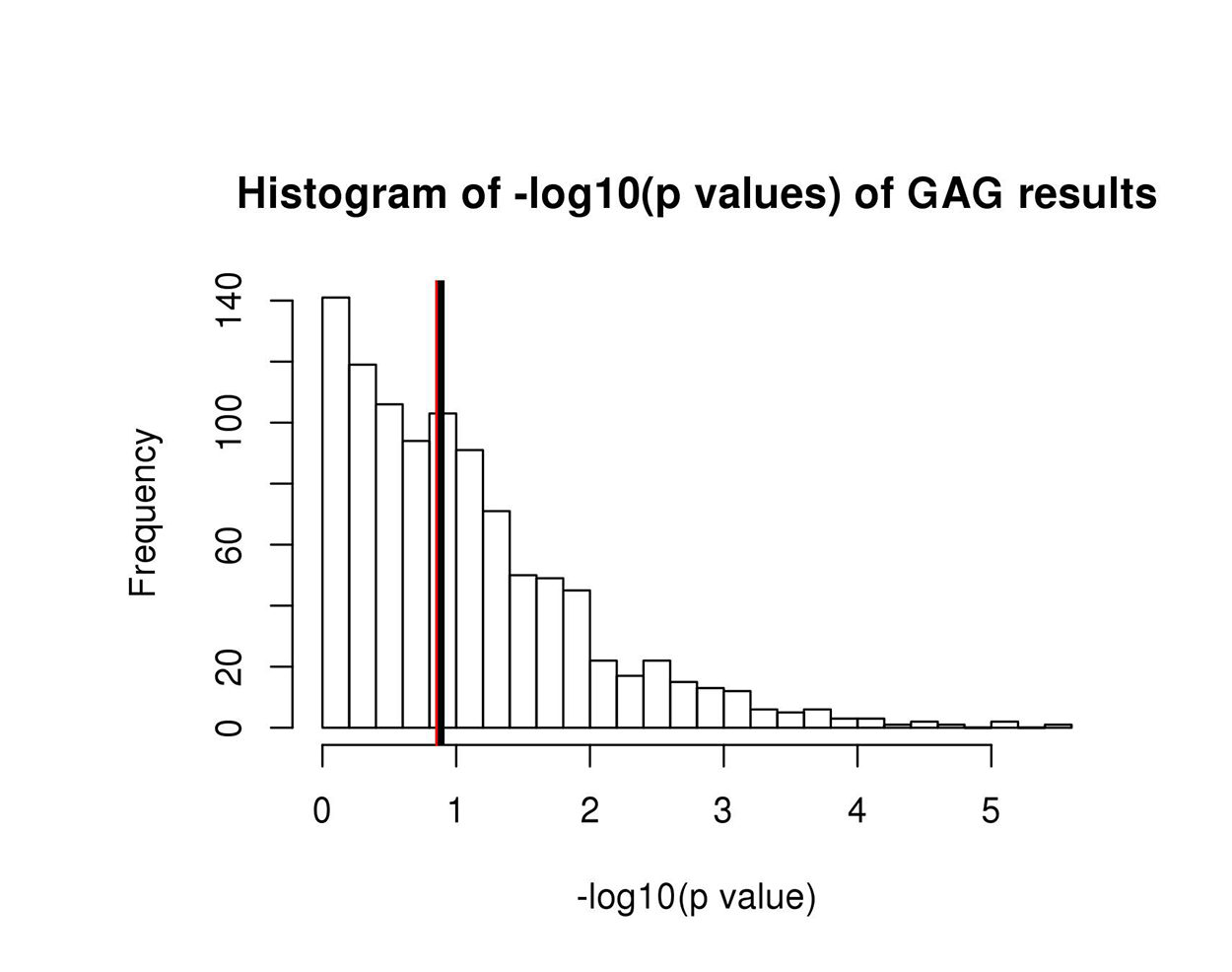
**

**Supplementary figure S4**


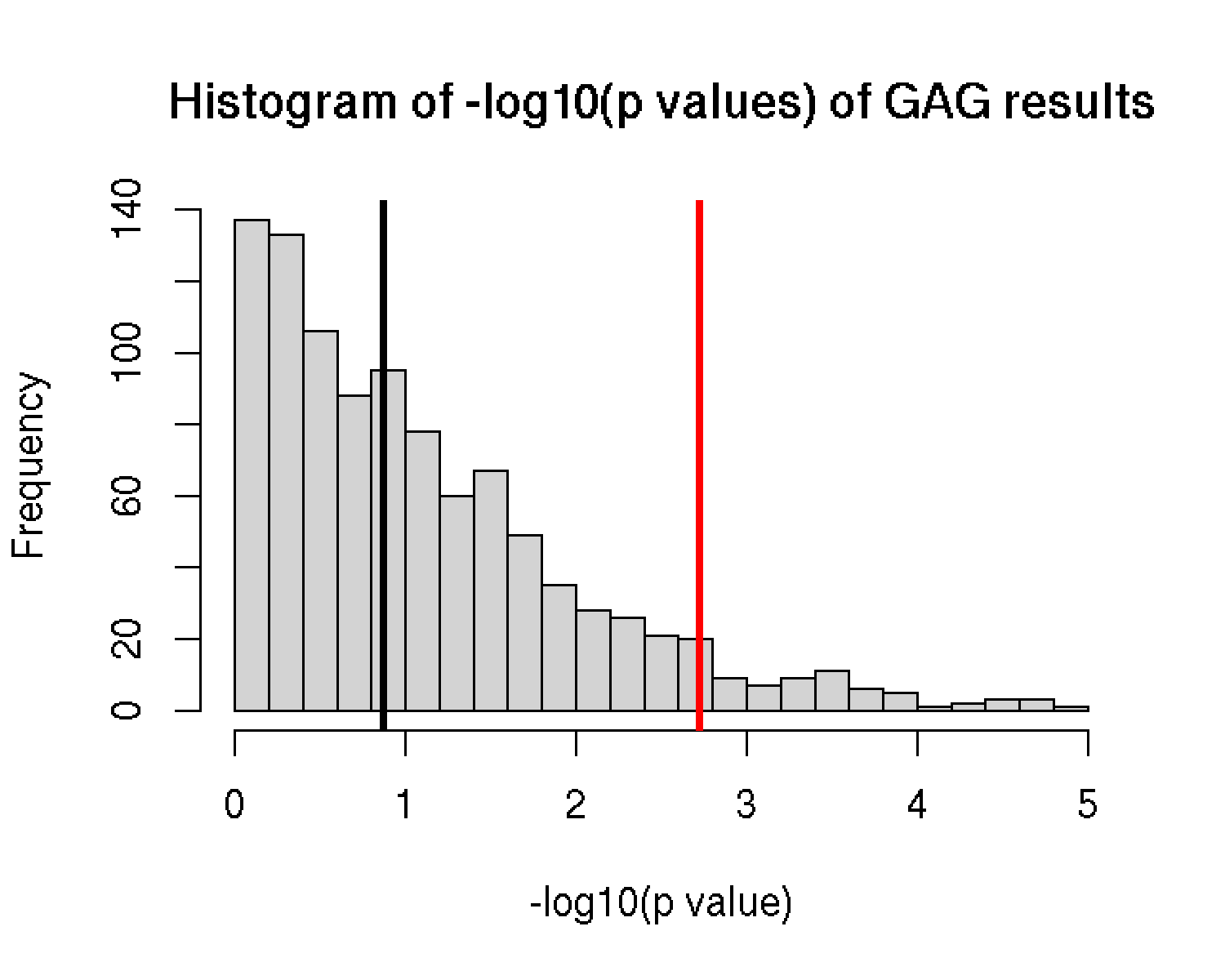


**Supplementary figure S5**


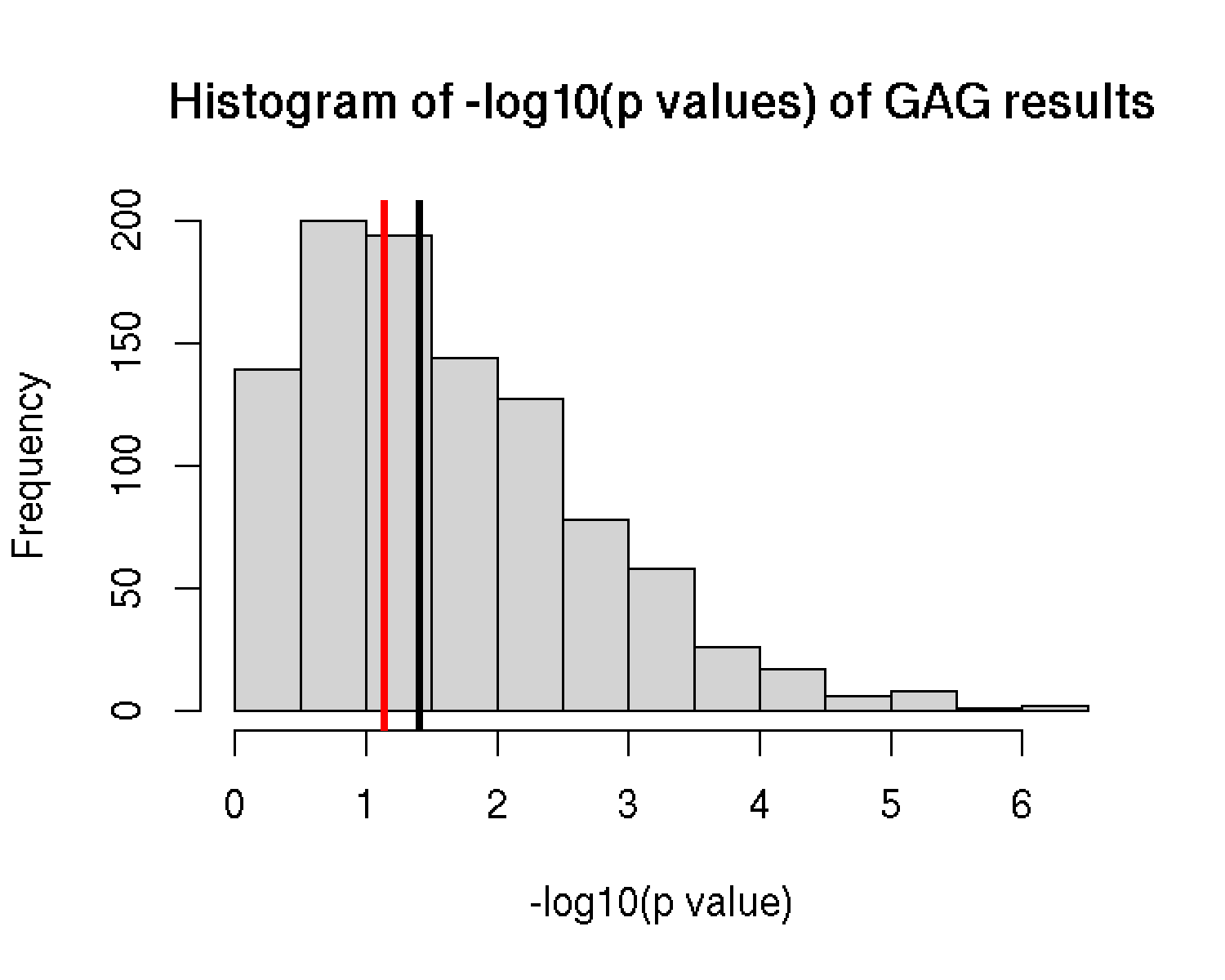


**Supplementary figure S6**


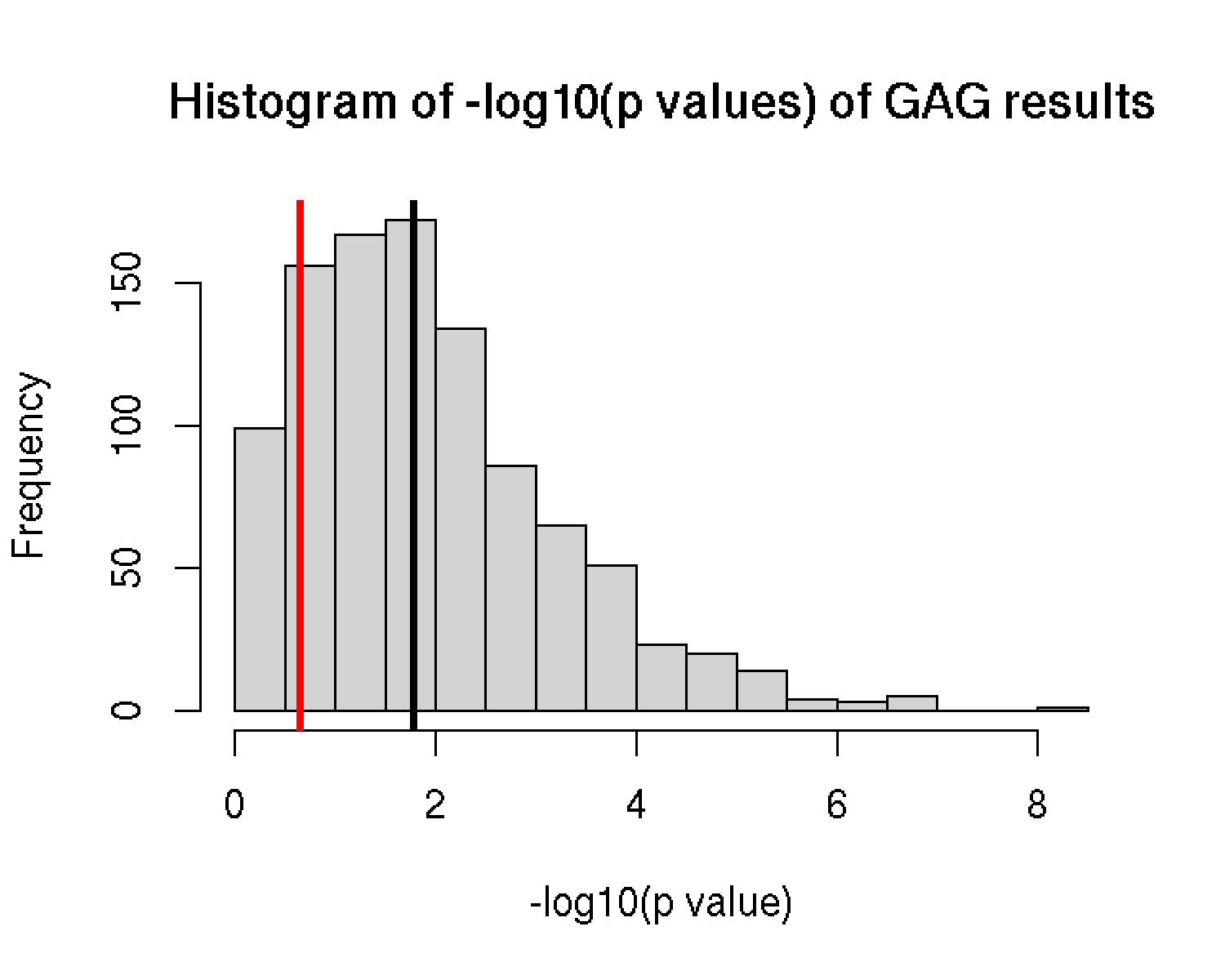


**Supplementary figure S7**


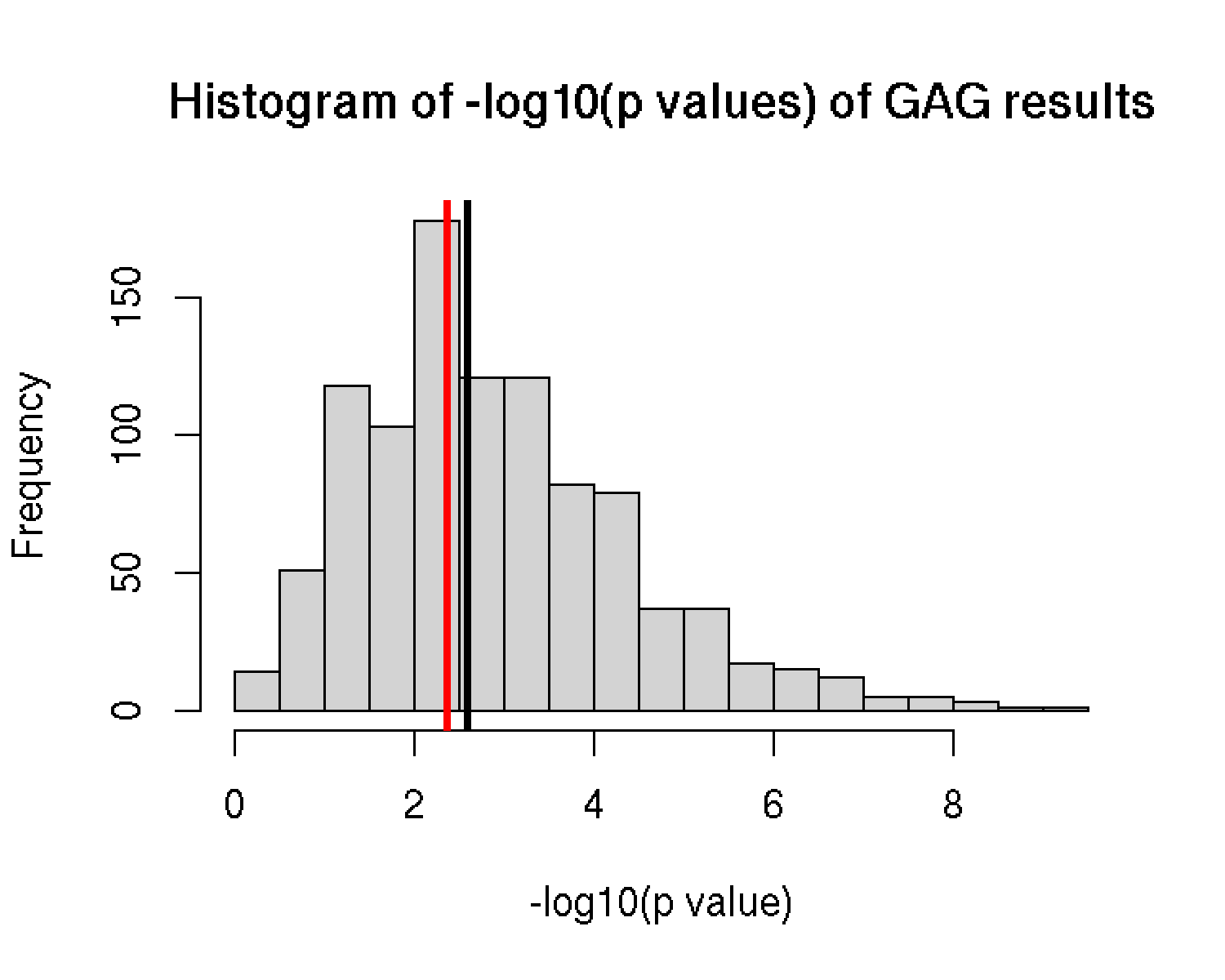


**Supplementary figure S8.**


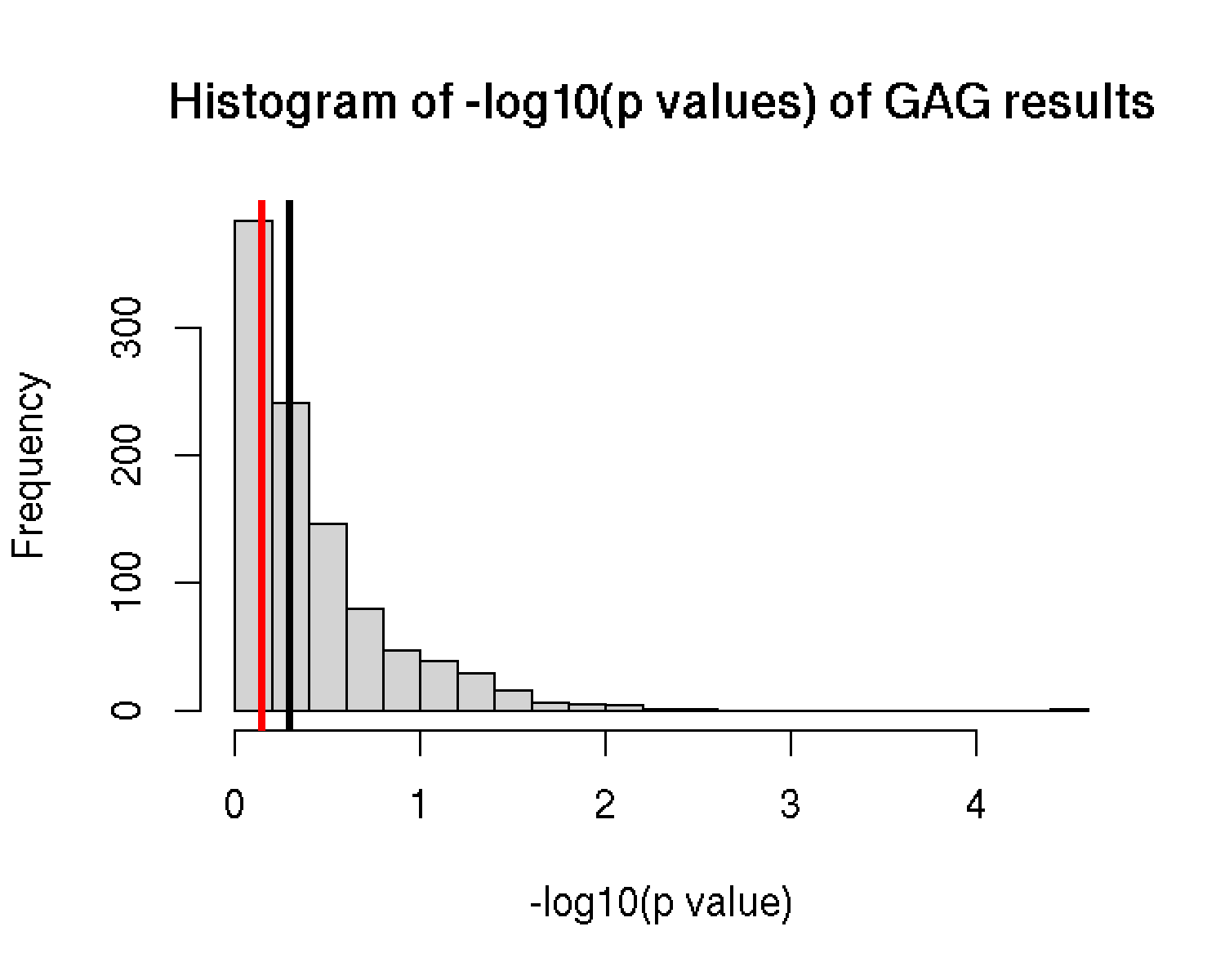


**Supplementary figure S9.**


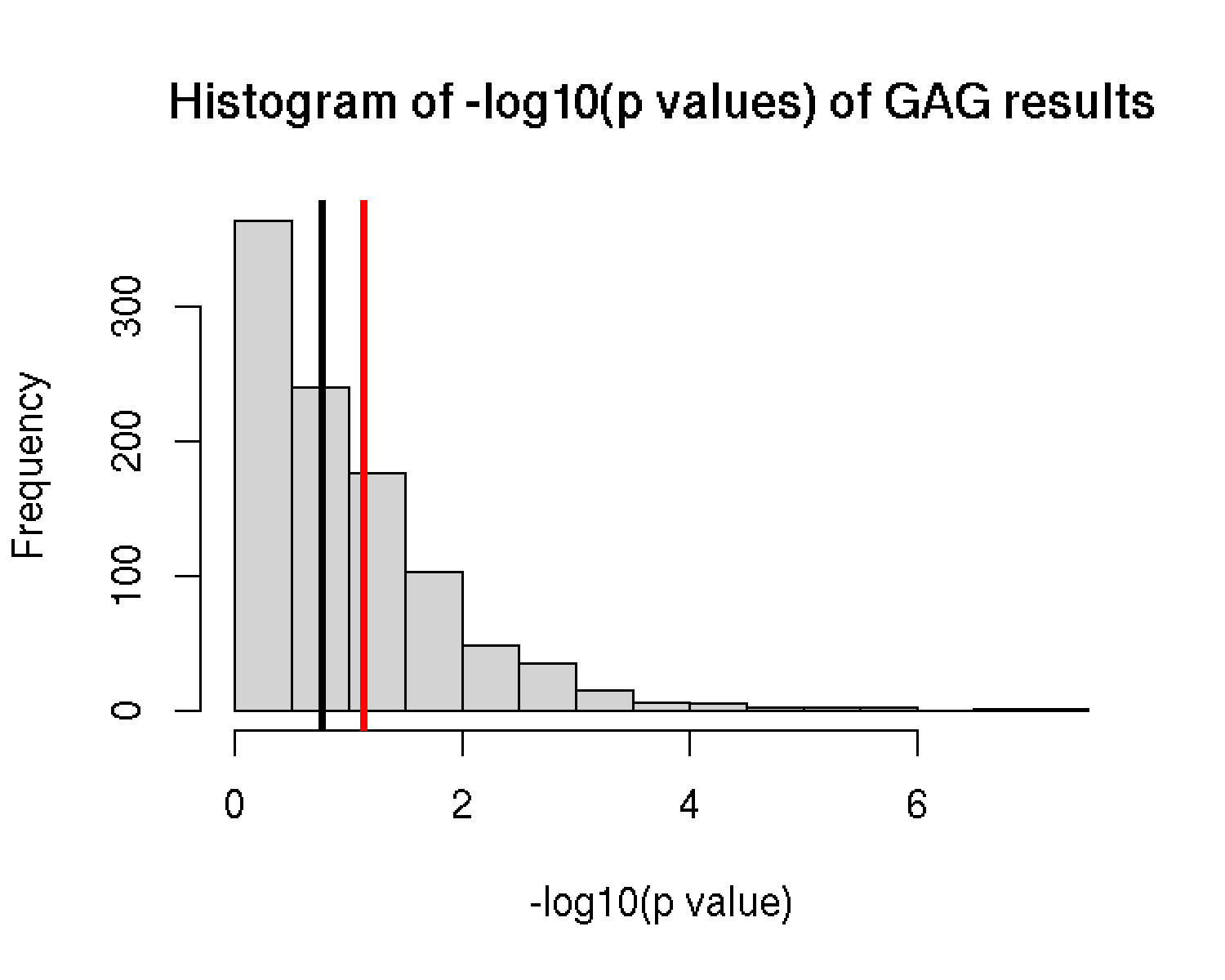


**Supplementary figure S10**


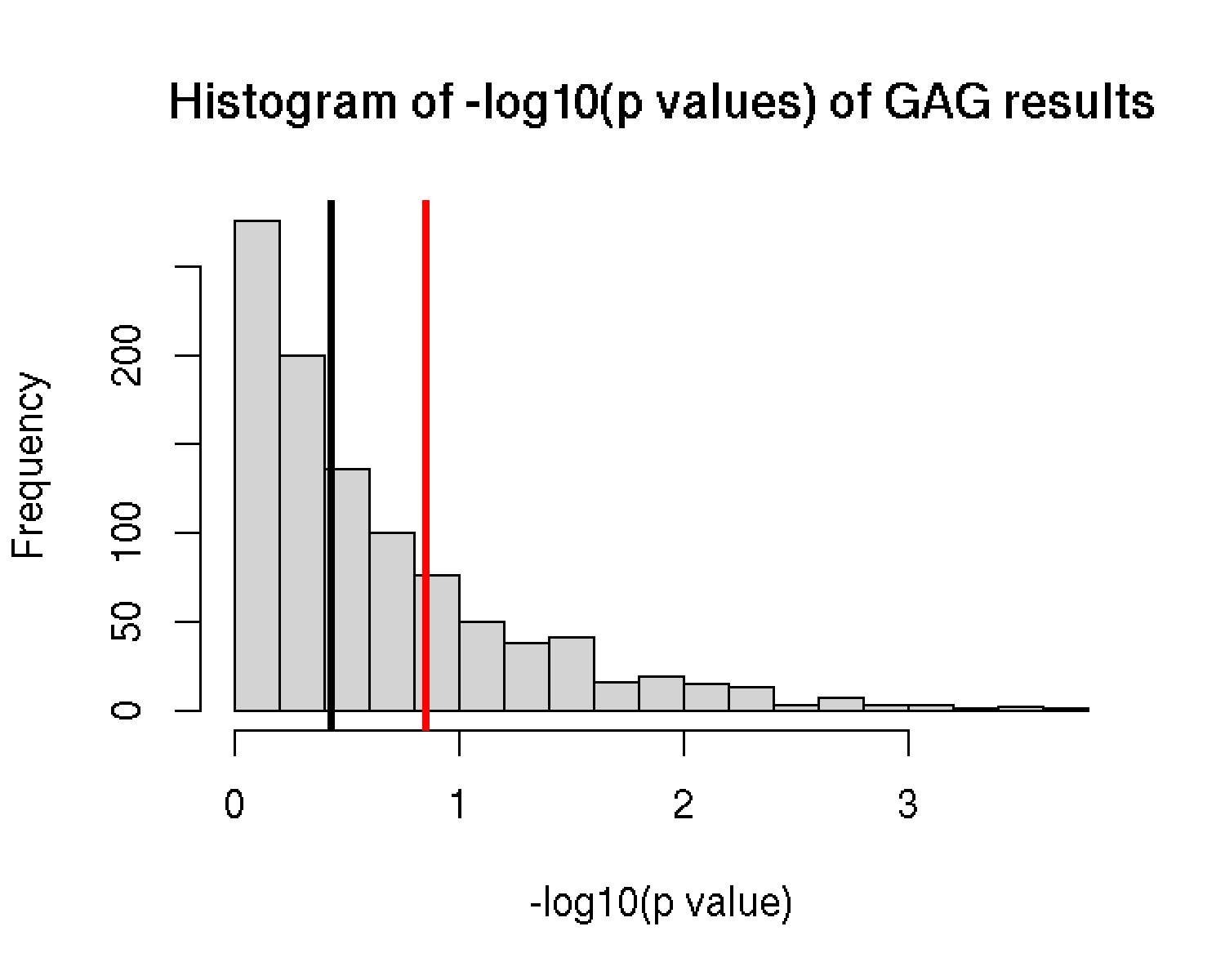


**Supplementary figure S11**


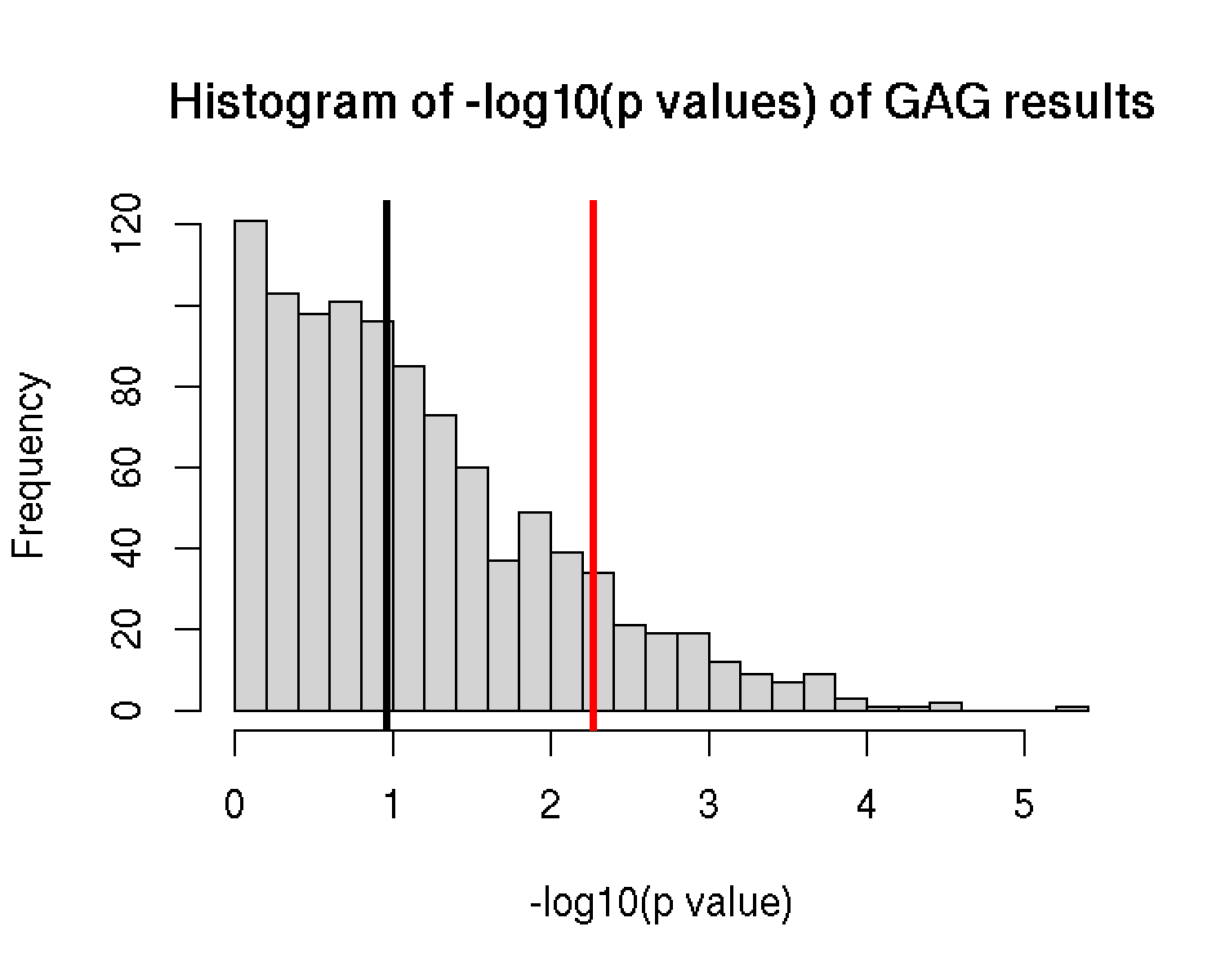


**Supplementary figure S12**

**
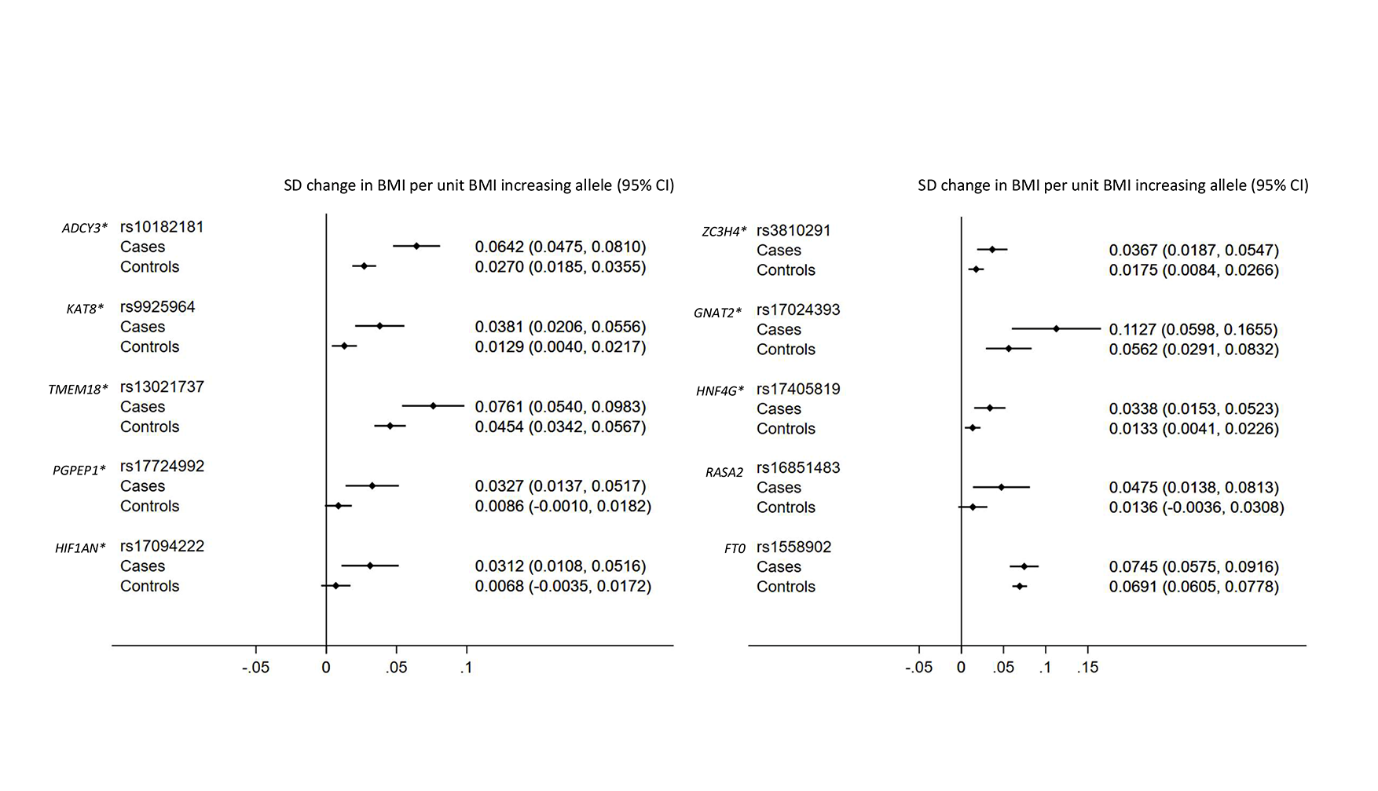
**

**Supplementary figure S13
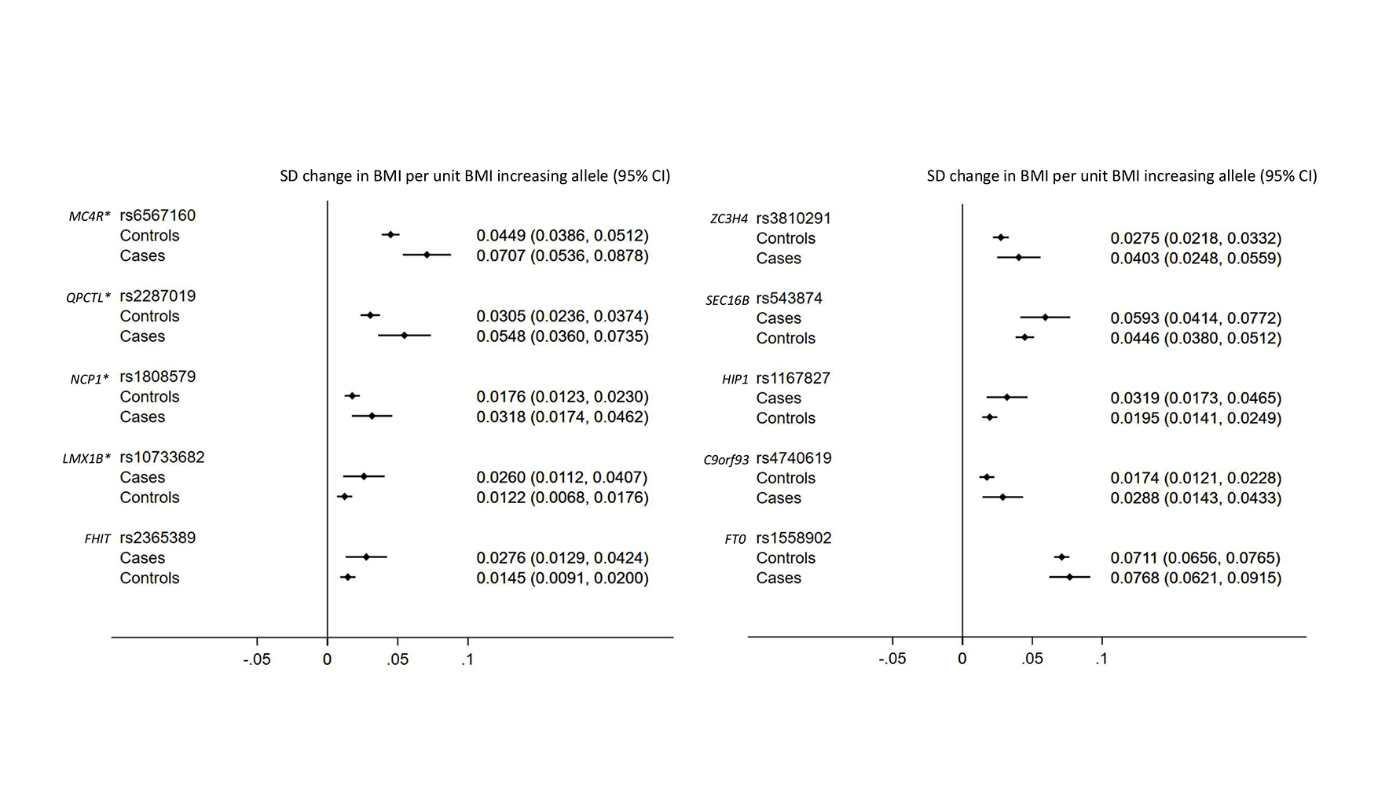
**

**Supplementary figure S14**

**
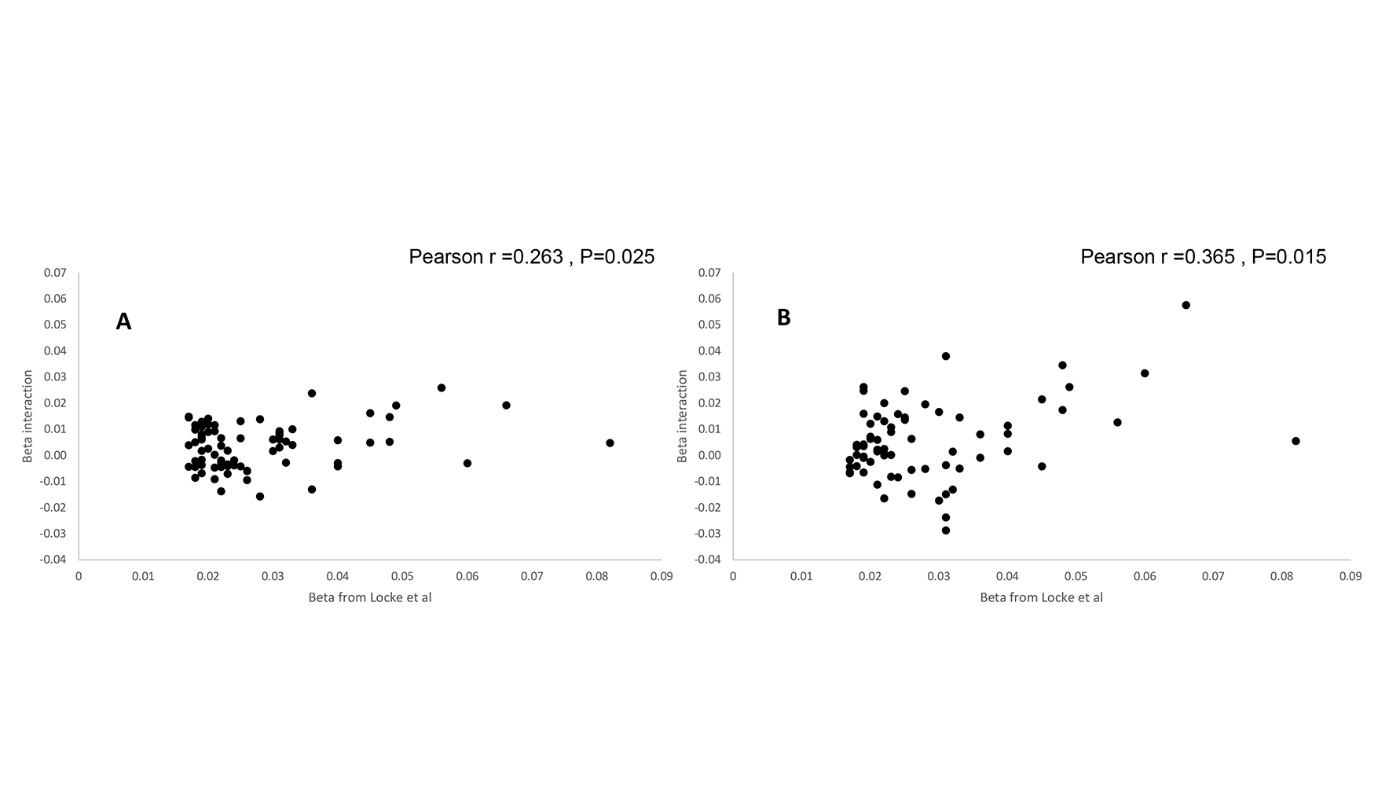
**

**Supplementary figure S15**

**
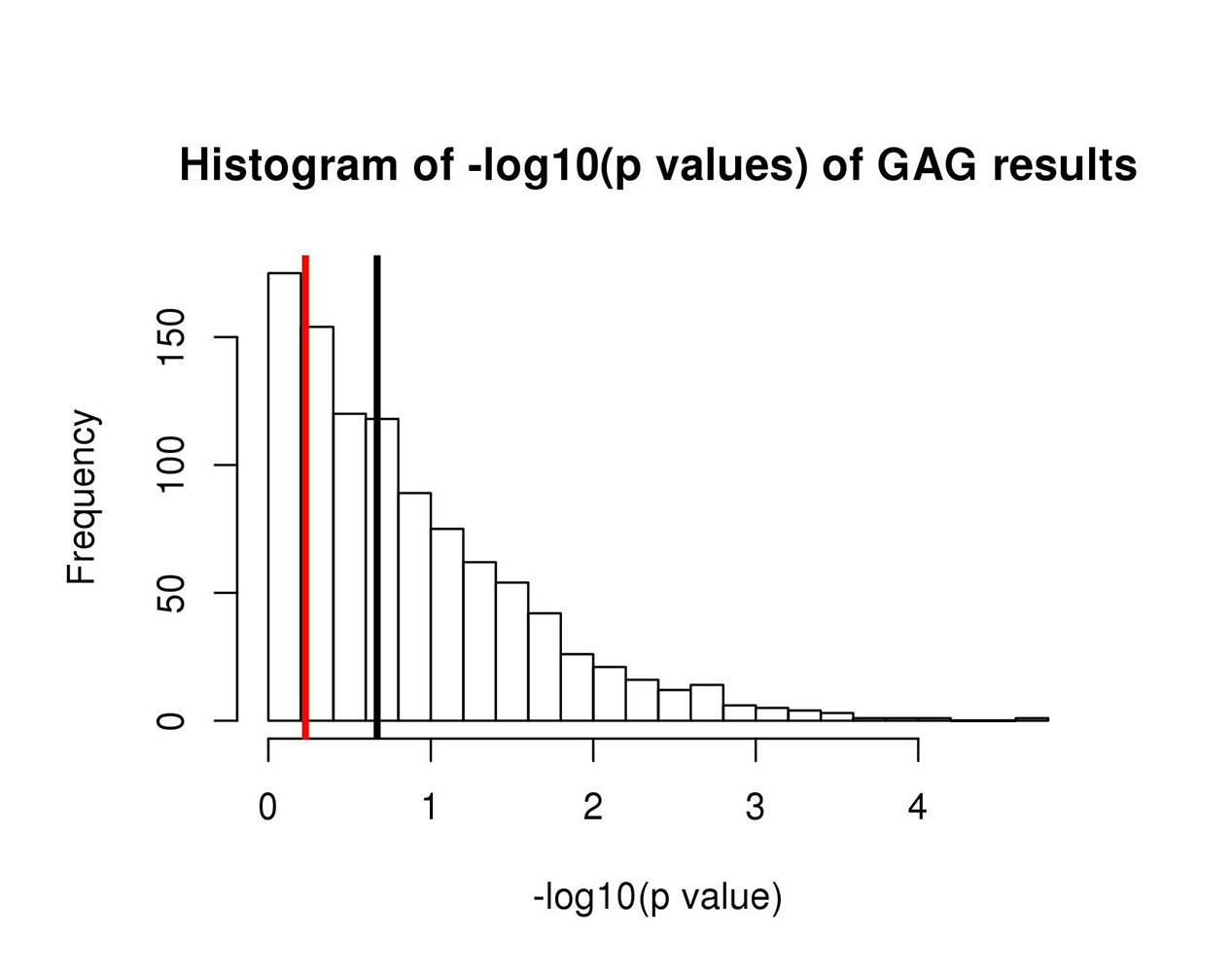
**

**Supplementary figure S16**

**
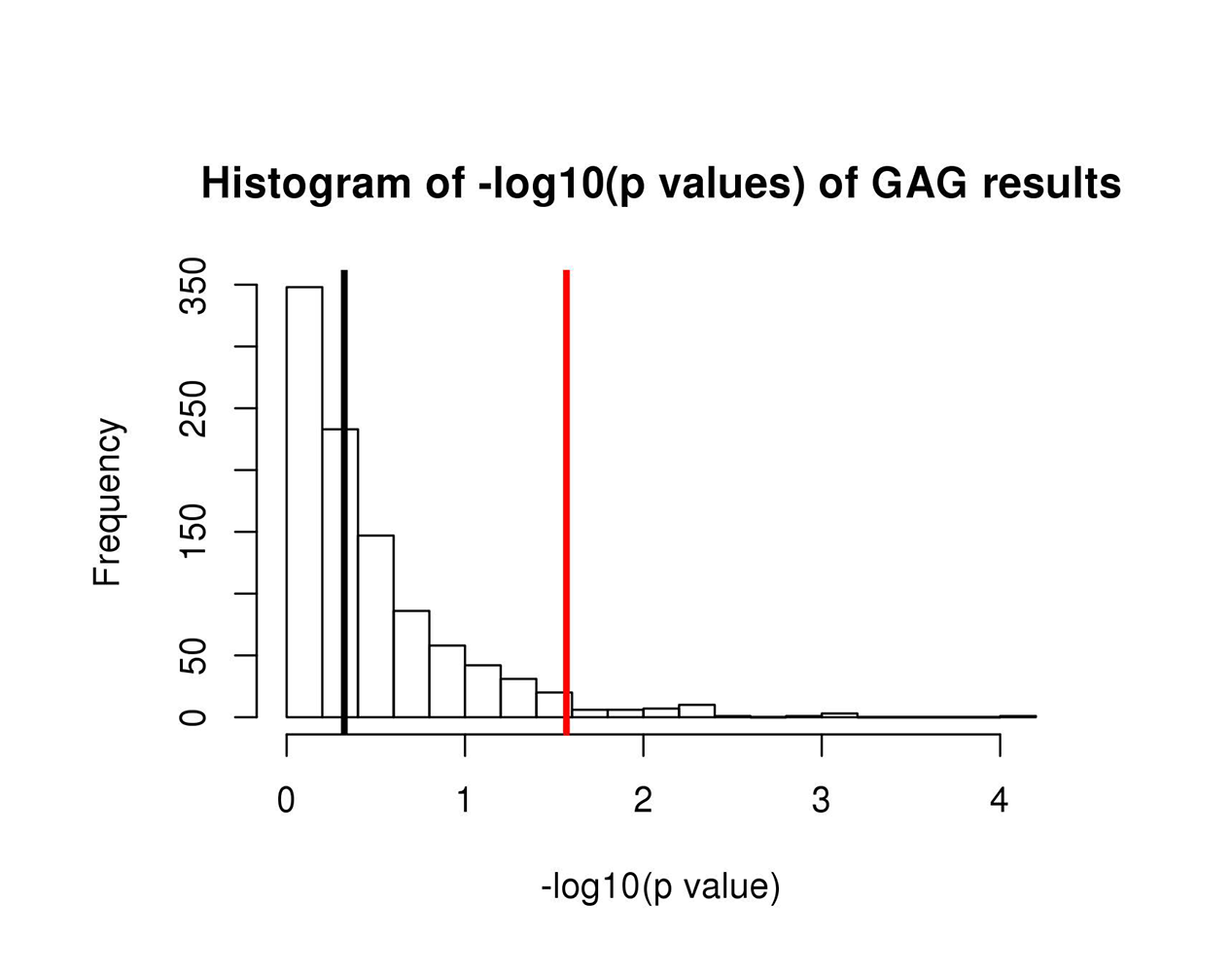
**
